# Sequential versus Standard Triple Therapy for First-Line *Helicobacter pylori* Eradication: An Update

**DOI:** 10.3390/antibiotics13020136

**Published:** 2024-01-30

**Authors:** Olga P. Nyssen, Belén Martínez, Francis Mégraud, Vincenzo Savarino, Carlo A. Fallone, Franco Bazzoli, Javier P. Gisbert

**Affiliations:** 1 Gastroenterology Unit, Hospital Universitario de La Princesa, Instituto de Investigación Sanitaria Princesa (IIS-Princesa), Universidad Autónoma de Madrid (UAM), and Centro de Investigación Biomédica en Red de Enfermedades Hepáticas y Digestivas (CIBERehd), 28006 Madrid, Spain; bemartbe@hotmail.com; 2 INSERM U1312 BRIC, Université de Bordeaux, 33000 Bordeaux, France; francis.megraud@u-bordeaux.fr; 3 Dipartimento di Medicina Interna e Specialita Mediche, Universita di Genova, 16132 Genova, Italy; vsavarin@unige.it; 4 Division of Gastroenterology and Hepatology, McGill University Health Centre, Montreal, QC H4A 3J1, Canada; carlo.fallone.med@ssss.gouv.qc.ca; 5 Dipartimento di Scienze Mediche e Chirurgiche, Università degli Studi di Bologna, 40138 Bologna, Italy; franco.bazzoli@unibo.it

**Keywords:** *H. pylori*, sequential therapy, standard triple therapy, eradication therapy, first-line therapy, antibiotic resistance, clarithromycin, systematic review, meta-analysis

## Abstract

Background: non-bismuth sequential therapy (SEQ) was suggested as a first-line anti-*Helicobacter pylori* treatment alternative to standard triple therapy (STT). Methods: We conducted a systematic review with a meta-analysis of randomized controlled trials (RCTs) comparing the efficacy of 10-day SEQ vs. STT (of at least 7 days) using bibliographical searches up to July 2021, including treatment-naïve adult or children. The intention-to-treat (ITT) eradication rate and the risk difference (RD) were calculated. Results: Overall, 69 RCTs were evaluated, including 19,657 patients (9486 in SEQ; 10,171 in STT). Overall, SEQ was significantly more effective than STT (82% vs. 75%; RD 0.08; *p* < 0.001). The results were highly heterogeneous (*I*^2^ = 68%), and 38 studies did not demonstrate differences between therapies. Subgroup analyses suggested that patients with clarithromycin resistance only and all geographical areas but South America could benefit more from SEQ. Both therapies have evolved over the years, showing similar results when STT lasted 14 days; however, a tendency toward lower SEQ efficacy was noted from 2010 onwards. Conclusions: Prior to 2010, SEQ was significantly more effective than STT, notably when 7-day STT was prescribed. A tendency toward lower differences between SEQ and STT has been noted, especially when using 10-day STT. None of the therapies achieved an optimal efficacy and therefore cannot be recommended as a valid first-line *H. pylori* treatment.

## 1. Introduction

*Helicobacter pylori* (*H. pylori*) infects over 50% of the adult population globally [1] and is known to be associated with a wide range of upper gastrointestinal diseases including gastritis, peptic ulcer disease (PUD) and gastric cancer. At present, the Maastricht IV Consensus [2] and the Kyoto consensus [3] recommend *H. pylori* eradication for all individuals infected with *H. pylori*.

Since 1997, a global panel of experts who convened in consensus conferences has consistently advocated for a primary approach to *Helicobacter pylori* eradication known as triple therapy. This approach prescribes the simultaneous use of a proton pump inhibitor (PPI) alongside two antibiotics, administered twice daily [4,5,6,7,8]. Typically, this regimen combines clarithromycin with either amoxicillin or nitroimidazole (metronidazole or tinidazole) [5,9]. However, a recent alternative has emerged in the form of sequential therapy (SEQ), challenging the traditional standard triple therapy (STT). SEQ involves a unique strategy, where a PPI and amoxicillin are administered twice daily for the initial five days, followed by a combination of a PPI, clarithromycin, and nitroimidazole twice daily for the subsequent five days [10,11].

The efficacy of STT is inversely related to the bacterial load, with higher eradication rates achieved in those with a low bacterial density in the stomach [12,13]. Consequently, it has been proposed that the brief initial dual therapy employed in SEQ, involving amoxicillin, serves to decrease the gastric bacterial load. This reduction is believed to enhance the efficacy of the subsequent short course of triple therapy [14,15]. The first five days of amoxicillin and the PPI result in a marked reduction in *H. pylori* [14]. The subsequent stage of the regimen, which includes clarithromycin and a nitroimidazole, is intended to eliminate any remaining viable organisms. Moreover, it has been found that regimens containing amoxicillin reduce the development of secondary clarithromycin resistance [16].

SEQ may additionally contribute to improved eradication by potentially weakening cell walls during amoxicillin treatment, which could allow for higher concentrations of clarithromycin to be used. However, it is crucial to note that this potential effect has not been conclusively demonstrated. While the sequential administration of antibiotics is generally discouraged due to concerns about promoting drug resistance [17], SEQ uses amoxicillin, which rarely results in resistance [18].

While STT remains a widely employed treatment in clinical practice [19,20], there has been a notable decline in its efficacy for *H. pylori* eradication [19,20]. Since 2007, the eradication rates with STT have consistently fallen below 80%, a level deemed disappointing for addressing antimicrobial infections [21].

Also, in a previous prospective study [22] performed in the European context to assess *H. pylori* resistance to antibiotics and its association with antibiotic consumption, substantial levels of clarithromycin resistance were uncovered. As a result, the study strongly discouraged the empirical use of STT. These conclusions have been recently validated by the same researchers in a subsequent and updated analysis [23]. Furthermore, a recent publication [24] evaluating the antibiotic resistance prevalence and trends in patients infected with *H. pylori* in the period 2013–2020 as part of the European Registry on *H. pylori* management (Hp-EuReg) showed that bacterial resistance may notably vary among countries and within geographical regions in the same country [24].

In addition, a five-year analysis of the Hp-EuReg among 21,500 patients [19] demonstrated that STT is suboptimal in certain specific European settings. Hence, the ethical implications of persisting with STT have been a subject of scrutiny in the past, prompting questions about its ongoing use. Consequently, alternative therapies have been both previously and presently recommended as substitutes for STT [20,25].

The SEQ regimen is an alternative therapeutic approach; however, its efficacy in eradicating *H. pylori* infections needs confirmation, especially with the increasing prevalence of clarithromycin resistance [22,26]. Notably, a significant number of studies on SEQ conducted between 2008 and 2010 reported eradication rates below 90%, with some even dipping to 80% or less [27]. Moreover, the most commonly used SEQ therapy uses tinidazole, whilst in some studies metronidazole has been used. A review of SEQ [28] indicated that metronidazole-based regimens achieved significantly lower eradication rates compared to tinidazole-based regimens. This difference may be attributed to the markedly longer half-life of tinidazole, raising concerns about the efficacy of metronidazole for *H. pylori* therapy. It is worth noting that previous pooled analyses and meta-analyses primarily focused on studies conducted in Italy [28,29,30]. Recent studies from various regions have not shown a clear advantage of SEQ over STT, instead demonstrating comparable eradication rates [31,32].

Previous meta-analyses have compared STT with SEQ [29,30,31]. In our preliminary search, we identified several randomized controlled trials (RCTs) which were not included in the previous meta-analyses, prompting us to conduct an updated systematic review of RCTs with expanded database coverage and refined search strategies [33].

Therefore, the primary objective of the current review update was to conduct a meta-analysis of RCTs comparing the efficacy of the SEQ regimen with STT for the eradication of *H. pylori* infection. The second objective was to compare the incidence of adverse effects (AEs) associated with both STT and SEQ *H. pylori* eradication therapies.

## 2. Results

### 2.1. Overview of the Search

We retrieved 7259 citations from the following electronic databases: Cochrane Central Register of Controlled Trials (CENTRAL), MEDLINE (Ovid), EMBASE and CINAHL (see the Methods section for keyword searches); we found 15 additional references through manual searches and from the International Workshop of the European Helicobacter and Microbiota Study Group (EHMSG), the American Digestive Disease Week (DDW) and the United European Gastroenterology Week (UEGW) Congresses, up to July 2021.

After the removal of duplicates, we initially screened 6091 citations resulting from the electronic searches. Based on consideration of their titles and abstracts (see the Methods section below for inclusion/exclusion criteria), we excluded 5906 citations, while 185 papers were targeted for full-article review, either because they were potentially relevant or because not enough information was reported in the title and abstract to make a final decision.

After a review of the full texts, we finally included 69 publications in the review. All of them were RCTs.

**Figure 1:**
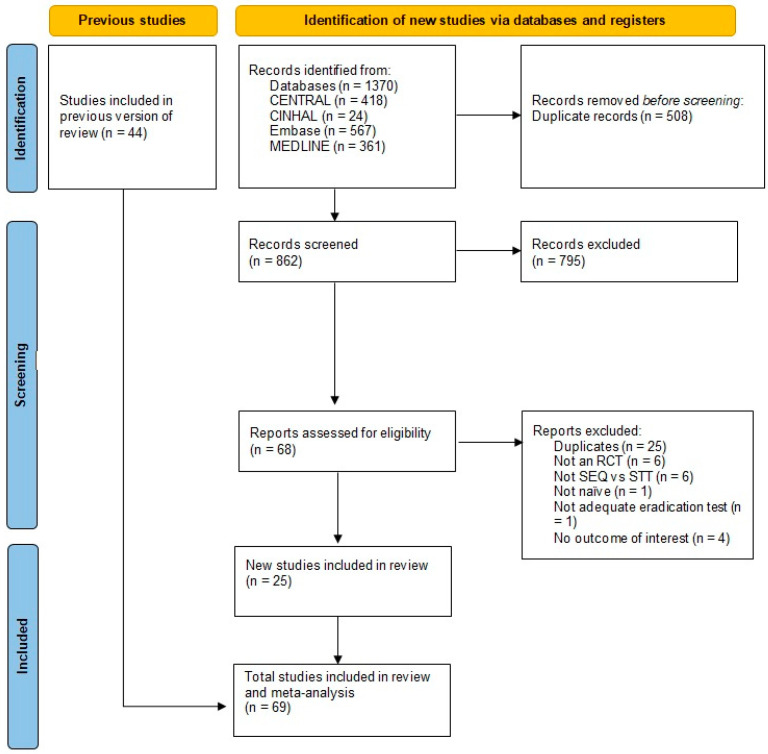
PRISMA flow diagram for updated systematic review. RCT: randomized clinical trial; SEQ: non-bismuth sequential therapy; STT: standard triple therapy; n: number of studies included.

The methodology employed for the identification and selection of studies, along with the number of studies identified at each stage, is reported in the PRISMA diagram (Figure 1) [34].

All included 69 RCTs had a standard parallel group design. See Table S1 for the full details of the characteristics of included studies.

The primary objective of almost all of the included studies was very similar and aimed to assess the efficacy of the 10-day SEQ versus STT. Two references reported different primary objectives: De Francesco, 2004b [35], who aimed to identify predictive factors for the outcome of *H. pylori* eradication using two therapeutic schemes (STT and SEQ), and Molina-Infante, 2010 [36], whose primary objective was to evaluate the cure rate of triple and sequential regimens containing clarithromycin or levofloxacin in a geographical area with a high failure rate of triple standard therapy.

For the purpose of synthesizing the evidence, we organized the included studies based on the specific endpoint under examination. This categorization involved assessing the overall eradication success with both SEQ and STT, as well as exploring various variables within subgroup analyses.

#### 2.1.1. Included Studies

Of the included studies, eleven were conducted in Italy [31,35,37,38,39,40,41,42,43,44,45], twelve in Korea [46,47,48,49,50,51,52,53,54,55,56,57], nine in China [58,59,60,61,62,63,64,65,66], two in India [67,68], two in Morocco [69,70], three in Iran [71,72,73], two in Puerto-Rico [74,75], five in Turkey [76,77,78,79,80], two in Thailand [81,82]), two in Taiwan [83,84], two in Saudi Arabia [85,86], and one each in Spain [36], Latin America [87], Poland [88], Belgium [89], Slovenia [90], Kenya [91], Brazil [92], Japan [93], Singapore [94], Mongolia [95]), Egypt [96], Romania [97], Portugal [98], Ireland [99] and Nepal [100].

Eight of the included studies were published before 2010.

Seven studies [59,62,65,86,88,89,91] published between 2010 and 2017 assessed the efficacy of 10-day SEQ versus STT in children.

Sixteen studies [35,36,39,40,42,46,48,63,64,67,71,79,81,82,87,95] assessed the efficacy of SEQ versus STT in either or both non-ulcer disease (NUD) and PUD participant groups. The success of eradicating the infection was documented separately for each group, and the findings were combined during the subgroup analysis corresponding to each group. The sample sizes in the included studies exhibited substantial variation, spanning from nine participants within both the STT and SEQ arms in Ali Habib HS, 2013 [86], to 650 participants within the SEQ arm and 650 participants in the STT arm in Liou, 2016 [83].

According to the specified eligibility criteria, all studies in the analysis involved a comparison between the 10-day SEQ and STT. STT encompassed various regimen durations (7, 10 and 14 days) and different antibiotic doses, including both high and standard doses. In contrast, SEQ employed different types of nitroimidazoles, such as metronidazole and tinidazole. Additionally, both regimens exhibited variations in the type and dosage (low, standard or high) of proton pump inhibitors, which included omeprazole, lansoprazole, pantoprazole, rabeprazole or esomeprazole. Four studies [31,52,53,97] used double-dose PPIs and two studies [45,73] used low-dose PPIs in both treatment arms.

*H. pylori* eradication proportion with SEQ ranged from 42% in Laving, 2013 [91], to 96% in the Italian study by Focareta, 2002 [37].

#### 2.1.2. Excluded Studies

The total number of studies excluded after the first screening was 5906. We then excluded 116 studies during the full-text review mainly because they did not fulfil the following criteria: not RCT studies, not comparing SEQ vs. STT, not treating naïve patients or not using a proper test to assess eradication.

### 2.2. Quality of the Included Studies

**Figure 2:**
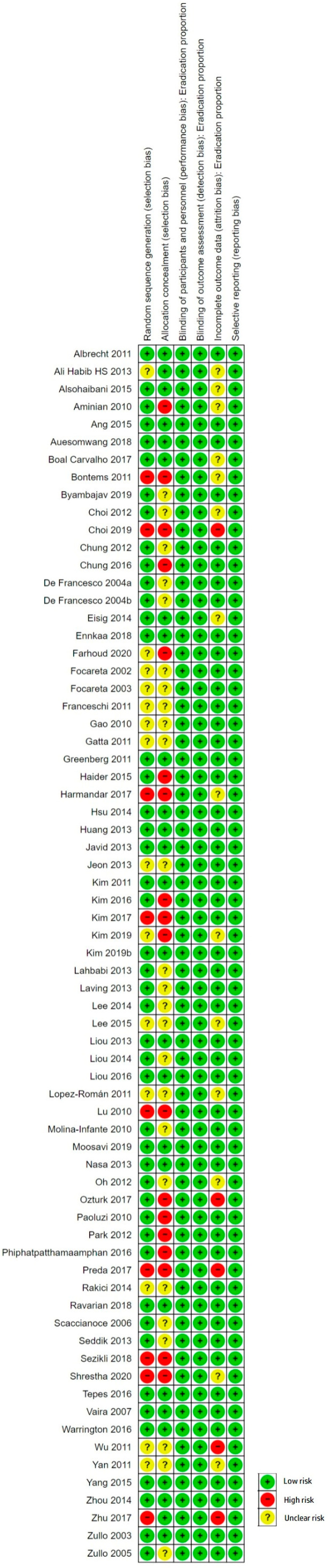
Risk of bias summary: review authors’ judgements about each risk of bias item (low risk, unclear risk and high risk) for each included study [31,35,36,37,38,39,40,42,43,44,46,47,48,49,50,51,52,53,54,55,56,57,58,59,60,61,62,63,64,66,67,68,69,70,71,72,73,74,75,76,77,78,79,80,81,82,83,84,85,86,87,88,89,90,91,93,94,95,96,97,98,99,100,101,102,103,104].

**Figure 3:**
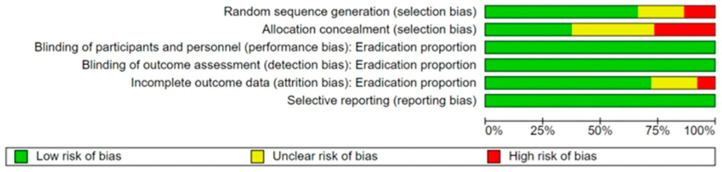
Risk of bias graph: review authors’ judgements about each risk of bias item presented as percentages across all included studies.

Potential biases were identified in all domains assessed; however, most of them were not influencing the primary outcome. Further details are reported in File S1. Figure 2 and Figure 3 summarize the risk of bias in the findings of the included studies.

### 2.3. Effects of Interventions

#### 2.3.1. Overall *H. pylori* Eradication

**Figure 4:**
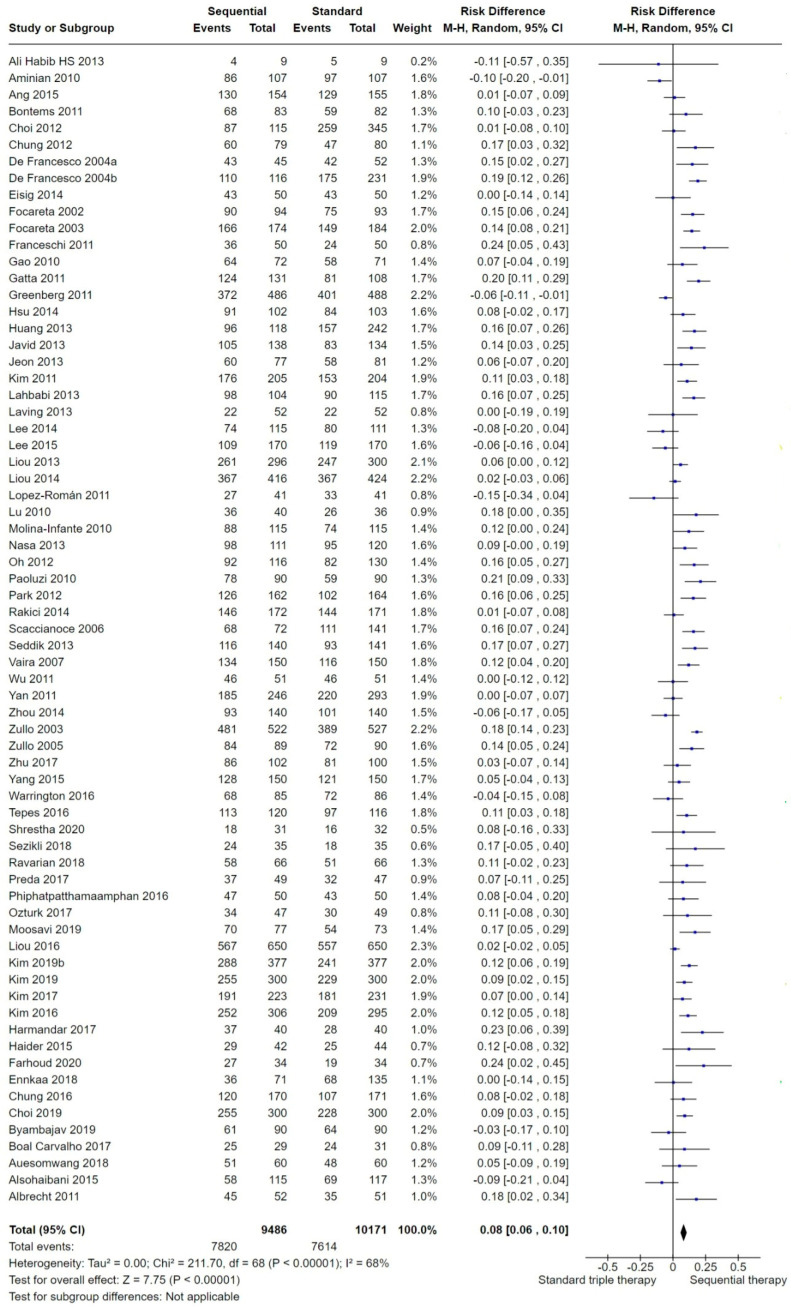
Forest plot of comparison: sequential therapy versus standard triple therapy. Eradication proportion. M-H: Mantel–Haenszel; CI: confidence interval [31,35,36,37,38,39,40,42,43,44,46,47,48,49,50,51,52,53,54,55,56,57,58,59,60,61,62,63,64,66,67,68,69,70,71,72,73,74,75,76,77,78,79,80,81,82,83,84,85,86,87,88,89,90,91,93,94,95,96,97,98,99,100,101,102,103,104].

We included 69 studies in the overall analysis comparing SEQ versus STT. In the overall analysis, when aggregating data and focusing solely on the standard triple therapy (STT) arm, several studies randomized participants into up to three different STT arms based on the prescribed therapy length (7, 10 and 14 days). To maintain randomization and ensure equal weighting among the included studies in the overall analysis of eradication, we presented the final overall proportion of participants cured with STT as a single figure. This was achieved by combining the number of participants cured in each of the STT arms to which they were randomized in the study, as detailed in the section addressing issues in the unit of analysis. Current meta-analysis showed that in the intention-to-treat (ITT) analysis, the overall eradication success was higher with SEQ compared to STT (*p* < 0.001; Figure 4). The risk difference (RD) for the overall ITT eradication of *H. pylori* was 0.08 (95% confidence interval (CI) 0.06 to 0.1; participants = 19,657; 69 studies) and the number needed to treat for an additional beneficial outcome (NNTB) was 14 with a 95% CI from 12 to 16. The results were highly heterogeneous (*I*^2^ = 68%).

Two studies [71,87] demonstrated a significantly higher efficacy with STT. Both of the studies assessed adults: Aminian, 2010 [71], a study from Iran, reported an ITT cure proportion of 91% and 80% with STT and SEQ, respectively. Greenberg, 2011 [87], a multicenter trial in Latin America, reported an ITT cure proportion of 82% and 76% with STT and SEQ, respectively. Nine other studies [64,74,75,80,85,86,87,95] showed better efficacy of STT compared to SEQ, although differences between therapies were not statistically significant.

One study (Laving, 2013 [91]) reported the same ITT eradication in both treatment arms but very different PP eradication. The reason is that the test for assessment of *H. pylori* eradication was not performed in several participants allocated to the SEQ treatment arm. The per protocol (PP) analysis reported that 22 of 26 participants were cured in the SEQ arm, while 22 of 45 were cured in the STT arm.

Thirty-four of the included studies did not demonstrate any clinical benefit for one regimen over the other.

The subgroup analyses reported below show the effect of different variables on the efficacy of both eradication treatments.

#### 2.3.2. Geographic Region

**Figure 5:**
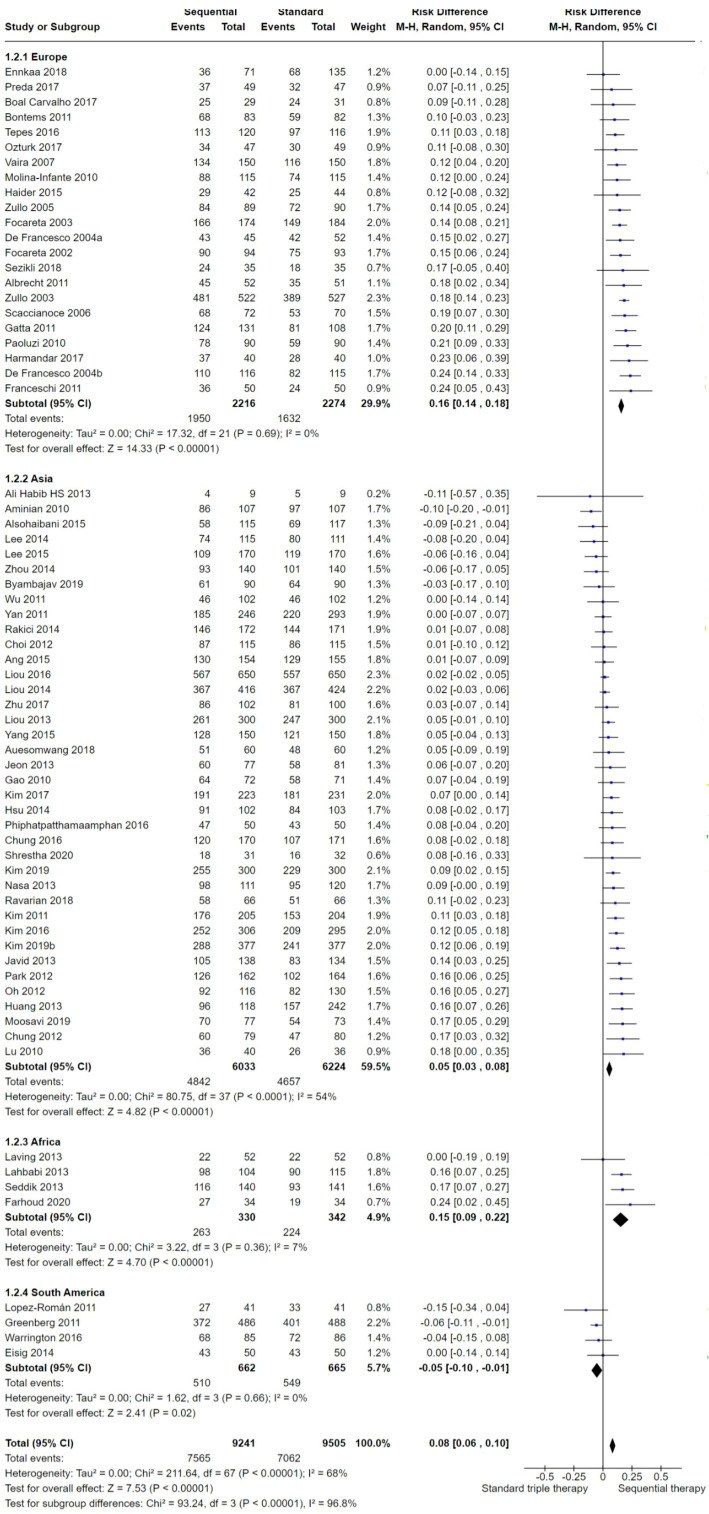
Forest plot of comparison: sequential therapy versus standard triple therapy. Geographic region. M-H: Mantel–Haenszel; CI: confidence interval. [31,35,36,37,38,39,40,41,42,43,44,46,47,48,49,50,51,52,53,54,55,56,57,58,59,60,61,62,63,64,65,66,67,68,69,70,71,72,73,74,75,76,77,78,79,80,81,82,83,84,85,86,87,88,89,90,91,93,95,96,97,98,99,100,102,103,104].

Over half (n = 38) of the included studies were conducted in Asia, over one-third (n = 22) were conducted in Europe and four took place each in South America and Africa (Figure 5).

Thirty-eight of the studies [46,47,48,49,50,51,52,54,55,56,57,58,59,60,61,62,63,65,67,68,71,72,73,81,82,83,84,85,86,93,95,100,104] were performed in Asia (mainly China and Korea but one in Japan, one in Singapore and one in India). Twenty-two were conducted in Europe [31,35,36,37,38,39,40,41,42,43,44,66,77,78,79,80,88,89,90,97,98,99,103]. The remaining studies were performed in South America [74,75,87,102], Africa [69,70,91,96], Saudi Arabia [86] or Turkey [76,80]. All of them were conducted between 2011 and 2021.

Studies published in Europe had the greatest RD for SEQ versus STT (RD 0.16, 95% CI 0.14 to 0.18; 4490 participants; 22 studies; *I*^2^ = 0%) when SEQ and STT were compared by subgroup analysis. Among the studies conducted in Europe (n = 22), most of them (n = 11) were conducted in Italy and the remaining in Spain, Belgium, Portugal, Poland and Slovenia. Seven of these studies [78,79,80,89,97,98,99] did not show significant differences between therapies, and participants receiving SEQ reported a greater cure proportion than those prescribed with STT. Also, the study by Molina-Infante, 2010 [36], reported differences between SEQ and STT at a borderline statistical level (RD 0.12, 95% CI 0.00 to 0.24). Studies conducted outside of Italy indicated a tendency toward lower efficacy with SEQ compared to STT than those studies conducted in Italy.

Studies conducted in Asia reported a reduced RD for SEQ versus STT (RD 0.05, 95% CI 0.03 to 0.08; 12,257 participants; 38 studies; *I*^2^ = 54%) than those in Europe, Africa or South America. Most of the studies were conducted in China or Korea. Twenty-five of them [47,50,52,53,54,58,60,61,63,64,65,66,68,72,81,82,83,84,85,86,93,94,95,105] did not show significant differences between SEQ and STT, and the results were heterogeneous. Among these 25 studies, 6 reported better efficacy with STT than with SEQ [52,53,64,85,86,95]. The previous tendency for better efficacy with SEQ shown in the European studies was reduced in the Asian studies.

Among the studies conducted in Africa, the risk difference for SEQ versus STT was 0.15 (95% CI 0.09 to 0.22; 672 participants; 4 studies; *I*^2^ = 7%). One study (Laving, 2013 [91]) did not show a significant difference between SEQ and STT. Note that four studies were included in this subgroup analysis and the reported CI was wide; however, patients displayed a higher benefit from SEQ than from STT.

The last region in this subgroup analysis is South America, with studies demonstrating a risk difference for SEQ versus STT of −0.05 (95% CI −0.10 to −0.01; 1327 participants; 4 studies; *I*^2^ = 0%), showing that STT was overall significantly better than SEQ. Three studies [74,75,102] did not show a significant difference between SEQ and STT. The remaining study (Greenberg, 2011 [87]) reported a greater cure proportion with STT than with SEQ, showing that participants in this subgroup could potentially benefit more from STT than from SEQ.

The subgroup analysis by geographic region is presented in Figure 5.

#### 2.3.3. Publication Date

**Figure 6:**
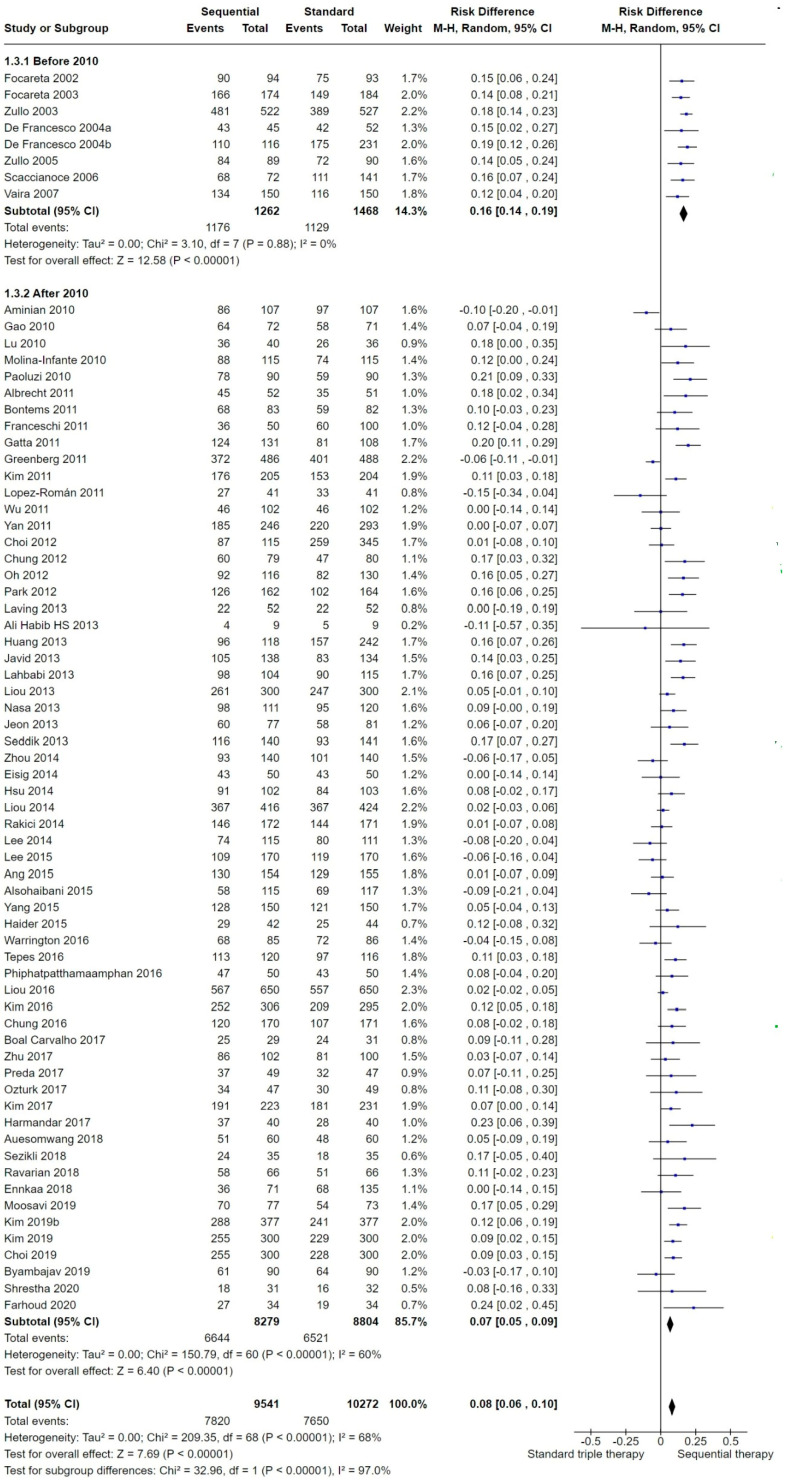
Forest plot of comparison: sequential therapy versus standard triple therapy. Publication date. M-H: Mantel–Haenszel; CI: confidence interval. [31,35,36,37,38,39,40,42,43,44,46,47,48,49,50,51,52,53,54,55,56,57,58,59,60,61,62,63,64,66,67,68,69,70,71,72,73,74,75,76,77,78,79,80,81,82,83,84,85,86,87,88,89,90,91,93,94,95,96,97,98,99,100,101,102,103,104].

Included studies were published between 2002 and 2021. Given the evolution in the *H. pylori* resistance to antibiotics, which has been reported as increasing over the years, we planned a subgroup analysis in order to explore heterogeneity with respect to the year the study was conducted/published. SEQ was reported to be significantly superior to STT in both the before and after 2010 subgroups and the treatment difference was supported by the test for subgroup differences (Chi^2^ = 32.96, df = 1 (*p* < 0.001), *I*^2^ = 97%) (Figure 6).

**Figure 7:**
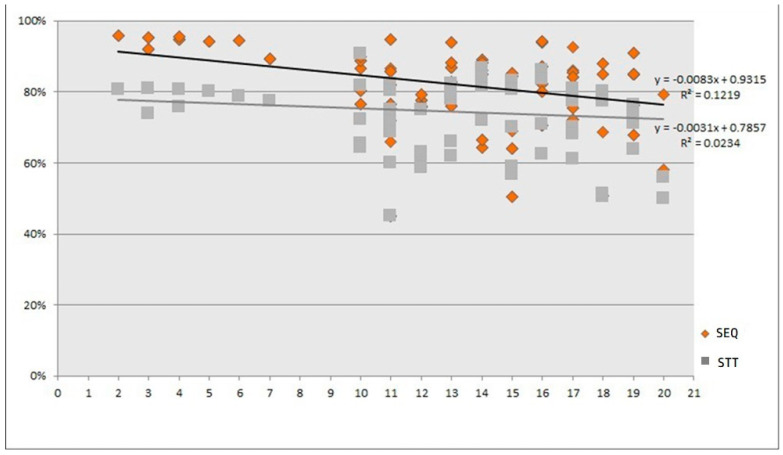
Weighted linear regression line in sequential therapy and standard triple therapy by year of publication. STT: standard triple therapy; SEQ: non-bismuth quadruple sequential therapy.

To evaluate the time trend and explore potential cut-off points for this tendency, a linear weighted regression model was generated (Figure 7). The regression was controlled by each study weight (measured using a random effects model) following the statistical assumptions of the rest of the meta-analysis. This model reported a tendency toward a decreased efficacy over the years in the overall mean eradication proportion for both therapies.

This exploratory model allowed us to identify a clear cut-off point for subgrouping. Before 2010, the number of included studies per year was small and offered equivalent results (note that all of the studies published before 2008 were of Italian origin); however, after 2010, the number of included trials per year increased, coming from other countries and regions, and started to offer more heterogeneous results. No studies published in 2008 or 2009 met the inclusion criteria of our review.

**Figure 8:**
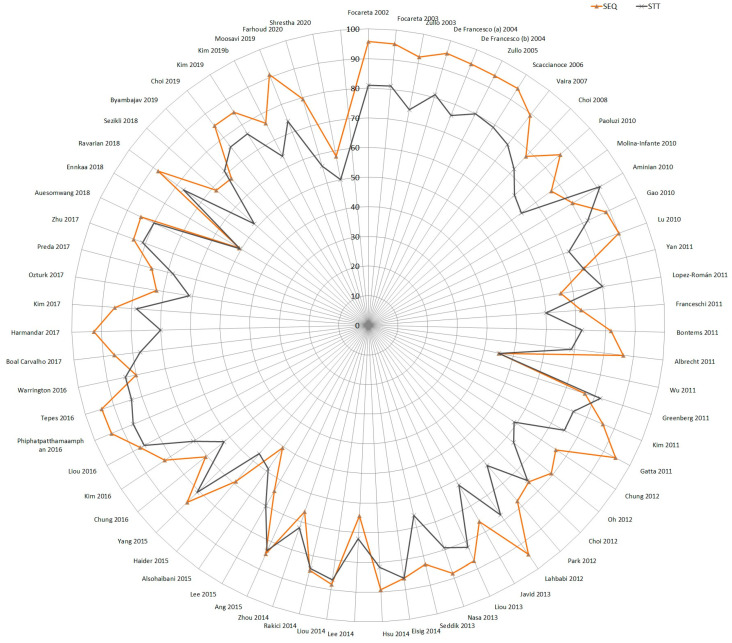
Radar chart depicting the eradication proportion for sequential therapy and standard triple therapy in each included study. STT: standard triple therapy; SEQ: non-bismuth quadruple sequential therapy. [28,31,35,36,37,38,39,40,42,44,46,47,48,49,50,51,52,53,54,55,56,57,58,59,60,61,62,63,64,66,67,68,69,70,71,72,73,74,75,76,77,78,79,80,81,82,83,84,85,87,88,89,90,91,93,94,95,96,97,98,99,100,101,102,103,104].

Furthermore, as shown in the radar chart of Figure 8, both STT and SEQ eradication rates appeared constant (or similar) between studies published before 2010, but after this year, eradication success was shown to be irregular over time, as represented by the various plots around the tendency lines between 2010 and 2021. The observed time lapses between 2008 and 2009 were therefore utilized as a cut-off point for the forest plot subgroup analyses. The forest plot (Figure 6) presented differences in the eradication proportions between SEQ and STT among the studies performed before and after the year 2010. The risk difference for SEQ versus STT for the studies published before 2010 was 0.16 (95% CI 0.14 to 0.19; 2730 participants; 8 studies; *I*^2^ = 0%), and the NNTB was 6 with a 95% CI from 5 to 7. The risk difference for SEQ versus STT for the studies published after 2010 was 0.07 (95% CI 0.05 to 0.09; participants = 17,083; studies = 61; *I*^2^ = 60%). The NNTB was 17 and the 95% CI was 13 to 20. Before 2010, studies reported higher eradication proportions and the RD was more than two times greater compared to studies published after 2010 (test for subgroup differences: Chi^2^ = 32.96, df = 1 (*p* < 0.001), *I*^2^ = 97.0%).

Two Italian studies [31,44] reported significantly larger risk differences for SEQ versus STT in the ‘after 2010′ subgroup. There was a decrease in SEQ eradication proportions below 90% starting in year 2010, except for four studies in which cure proportions were greater than or equal to 90% [31,59,69,90].

As previously noted in Figure 7, a decreased efficacy over the years was shown for both therapies; however, this trend was more pronounced for SEQ (−1.79% per year) than for STT (−0.9% per year), which matches the lower RD obtained in the ‘after 2010’ subgroup.

#### 2.3.4. Age of the Population

All but seven included studies were conducted in adults, with studies conducted in children [59,62,65,86,88,89,91] first published from 2010 onwards.

The pooled risk difference for the eradication of *H. pylori* with SEQ compared to STT in children was reported to be slightly higher than in adults (Figure S1). The risk difference in the children group was 0.11 (95% CI 0.05 to 0.17; participants = 1028; studies = 7; *I*^2^ = 14%), and for adults, the RD was 0.08 (95% CI 0.06 to 0.10; participants = 18,318; studies = 62; *I*^2^ = 70%). However, the test for subgroup differences was not significant (Chi^2^ = 0.76, df = 1 (*p* = 0.38), *I*^2^ = 0%) and differences between subgroups could not be clearly supported.

The NNTB in children was 10, with a 95% CI from 6 to 18, whereas in adults, the NNTB was 14, with a 95% CI from 12 to 16.

#### 2.3.5. Medical Condition: Non-Ulcer Disease (NUD) versus Peptic Ulcer Disease (PUD)

Sixteen studies [36,39,40,41,42,46,48,64,67,71,79,81,82,87,95] reported the baseline medical condition of the participants. The risk difference for SEQ versus STT in the PUD group was 0.07 (95% CI −0.01 to 0.15; participants = 1822; studies = 9; *I*^2^ = 81%), and in the NUD group, the RD was 0.07 (95% CI 0.01 to 0.14; participants = 2763; studies = 12; *I*^2^ = 81%). Differences between therapies were statistically not significant in the PUD group and were significant in the NUD group, both at a borderline level in favor of SEQ, according to the test for subgroup differences: Chi^2^ = 0.00, df = 1 (*p* = 0.97) and *I*^2^ = 0%) (Figure S2).

#### 2.3.6. Length of the Standard Triple Therapy (STT)

**Figure 9:**
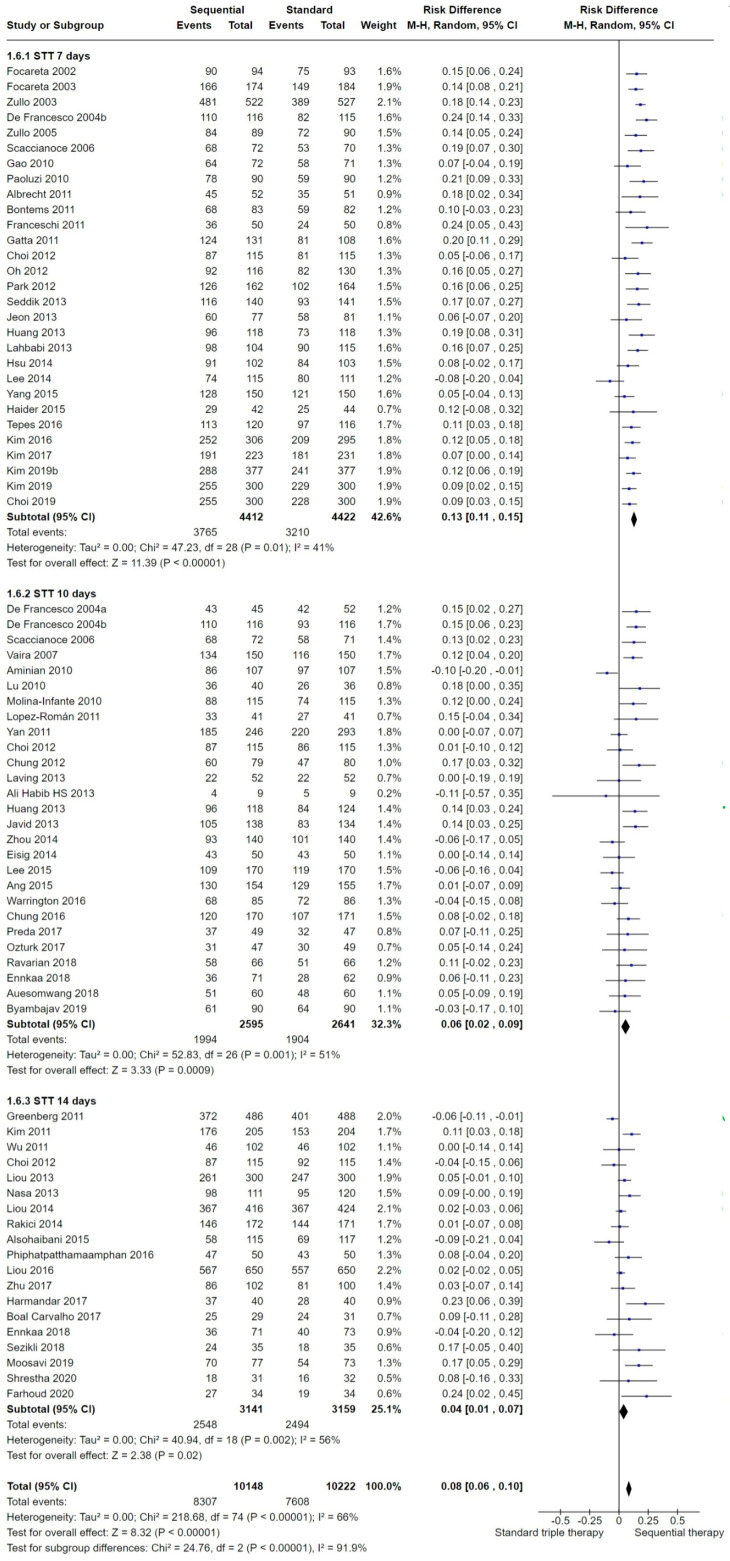
Forest plot of comparison: sequential therapy versus standard triple therapy length. M-H: Mantel–Haenszel; CI: confidence interval. [28,31,35,36,37,38,39,40,42,44,46,47,48,49,50,51,52,53,54,55,56,57,58,59,60,61,62,63,64,66,67,68,69,70,71,72,73,74,75,76,77,78,79,80,81,82,83,84,85,87,88,89,90,91,93,94,95,96,97,98,99,100,101,102,103,104].

This analysis compared 10-day SEQ versus 7-day (29 studies), 10-day (27 studies) and 14-day (19 studies) STT (Figure 9). SEQ was significantly better than 7-, 10- and 14-day STT. Note that some studies assessed different STT lengths and have been therefore included in the corresponding subgroups as appropriate [41,42,47,62,80].

In the subgroup analysis, the *H. pylori* eradication proportions among the different STT lengths were compared with 10-day SEQ. The risk difference in the 7-day STT group was 0.13 (95% CI from 0.11 to 0.15; participants = 8834; studies = 29; *I*^2^ = 41%). In the 10-day STT group, the risk difference was 0.06 (95% CI from 0.02 to 0.19; participants = 5236; studies = 27; *I*^2^ = 51%), and in the 14-day STT group, the RD was 0.04 (95% CI from 0.01 to 0.07; participants = 6300; studies = 19; *I*^2^ = 56%), showing a borderline statistical difference between therapies in favor of SEQ. The test for subgroup differences was significant (Chi^2^ = 24.76, df = 2 (*p* < 0.00001), *I*^2^ = 92%), supporting that there was a tendency to reduce the statistical differences between STT and SEQ treatments when longer STT regimens were prescribed.

The NNTB when STT lasted seven days was 8, with a 95% CI from 7 to 9, the NNTB when STT lasted 10 days was 22, with a 95% CI from 14 to 42, and the NNTB when STT lasted 14 days was 47, with a 95% CI from 24 to 504.

#### 2.3.7. Type of Nitroimidazole

We included 68 studies in this subgroup meta-analysis, where Liou 2014 [66], did not provide information on antibiotics nor on PPIs. Although we contacted the authors, the information was not supplied.

Forty-four and twenty-four studies used metronidazole and tinidazole, respectively, in patients treated with SEQ. Both subgroups showed better results with SEQ than with STT (Figure S3).

In the metronidazole group, the risk difference for SEQ versus STT was 0.07 (95% CI from 0.04 to 0.09; participants = 12,932; studies = 44; *I*^2^ = 60%). The NNTB was 16, with a 95% CI from 13 to 20. In the tinidazole group, the risk difference for SEQ versus STT was 0.12 (95% CI from 0.08 to 0.15; participants = 5574; studies = 24; *I*^2^ = 64%). The NNTB was 9, with a 95% CI from 7 to 10. In both subgroups, SEQ was shown to be statistically better than STT.

However, the differences between these two subgroups of participants treated with different nitroimidazole types were not significant for *H. pylori* eradication. Individual study risk differences did not particularly overlap, and heterogeneity was therefore substantial (test for subgroup differences: Chi^2^ = 5.79, df = 1 (*p* = 0.02), *I*^2^ = 82.7%).

#### 2.3.8. Acid Inhibition with Proton Pump Inhibitors (PPIs)

Both STT and SEQ regimens used different PPIs (omeprazole, lansoprazole, pantoprazole, rabeprazole or esomeprazole), as well as different PPI doses among the included studies. Subgroup analysis aimed to compare the efficacy of adjuvant medication within both treatment regimens. Acid inhibition was categorized based on the type and dose of the PPI, as outlined in the “Methods” section, following the equivalences generally accepted (omeprazole 20 mg = pantoprazole 40 mg, lansoprazole 30 mg, and rabeprazole and esomeprazole 20 mg).

We included 61 studies within this subgroup meta-analysis. Five studies [56,57,66,94,101,105] were excluded as they did not report data for PPIs (Figure S4). In Ang, 2015 [94], we contacted the first author for the PPI information, who reported that most of the participants were given omeprazole standard doses, although some of them had rabeprazole or esomeprazole. We therefore decided not to include these data in the subgroup analysis for consistency with the remaining included studies. We also excluded three studies using pediatric formulations by participants’ weight [59,62,65], as they cannot be pooled together with adult fixed-tablet doses.

Only two studies [45,73] evaluated low acid inhibition with lansoprazole 15 mg twice a day and pantoprazole 20 mg twice a day, respectively, yielding an RD for SEQ versus STT of 0.19 (95% CI from 0.09 to 0.29; 250 participants), showing higher efficacy in SEQ. The majority of studies (n = 55) evaluated standard doses of the PPI, showing a significant advantage in the use of SEQ versus STT (RD 0.08, 95% CI from 0.06 to 0.11; 14,754 participants). However, there was no significant increase in the efficacy of SEQ in the four studies [31,52,53,97] using high acid inhibition (RD 0.03, 95% CI from −0.12 to 0.18; 901 participants).

We found no differential effect based on levels of acid inhibition with PPIs (test for subgroup differences: Chi^2^ = 4.61, df = 2 (*p* = 0.10), *I*^2^ = 56.6%) (Figure S4).

#### 2.3.9. Bacterial Antibiotic Resistance

Most of the studies did not perform prior antibiotic susceptibility testing, and only 13 out of 69 (18.57%) studies reported eradication by bacterial antibiotic resistance (Figure S5).

We conducted subgroup meta-analyses, including the rates of *H. pylori* eradication in those participants with reported results, according to bacterial clarithromycin resistance, nitroimidazole resistance and dual resistance.

In the subgroup meta-analyses, participants with bacterial clarithromycin-resistance eradication were significantly better when treated with SEQ than with STT (63% vs. 40%, respectively). The RD among this subgroup of participants was 0.29 (95% CI from 0.14 to 0.44; participants = 384; studies = 13; *I*^2^ = 61%). The NNTB was 5, with a 95% CI from 3 to 7. Although there seemed to be a similar proportion in the nitroimidazole-resistance cure rates (83% versus 82%) as well as in the dual resistance group (62% versus 54%), these apparent advantages of SEQ did not reach statistical significance when compared to STT.

Differences between all subgroups were significant, although heterogeneity in the results was substantial (test for subgroup differences: Chi^2^ = 9.90, df = 2 (*p* = 0.007), *I*^2^ = 79.8%). Additionally, the RD for SEQ versus STT within the clarithromycin-resistance subgroup analysis was also greater compared to the RD of the overall subgroup meta-analysis (0.29 versus 0.12, respectively), meaning differences between treatment arms were even greater among those participants with primary resistances.

### 2.4. Safety Profile

Forty-four studies (64%) described common AEs such as abdominal pain, diarrhea, nausea, glossitis and vomiting, giving their incidence by treatment arms (Figure S6). The trial reports did not mention whether there were any serious AEs.

Within the SEQ arms, the incidence of AEs ranged from 2% in Aminian, 2010 [71], to 77% in Auesomwang, 2018 [82]. In the STT arm, the incidence ranged from 2% to 63% in the same aforementioned studies, respectively.

In the ITT analysis, the overall AE proportions showed no significant differences between SEQ and STT (26% versus 25%, respectively). The majority of studies were not able to demonstrate differences in the incidence rate of AEs between treatment groups. However, two studies [69,73] reported a higher incidence rate with STT; whereas two other studies [53,85] showed greater incidence with SEQ. The risk difference was RD 0.00 (95% CI from −0.01 to 0.02; participants = 12,681; studies = 44; *I*^2^ = 42%). The number needed to treat for an additional harmful outcome (NNTH) was 148.

A summary of findings (SoF) table (Table S2) was created using GRADEpro GDT for the main comparisons that could potentially affect the main outcome (eradication).

### 2.5. Compliance

Compliance rates were reported in 35 studies. However, compliance definitions varied across studies, being defined as “good compliance” in most of the studies if participants had taken between 90 and 95% of the prescribed pills. In one study (Greenberg, 2011 [87]), the authors did not specify a minimum intake and reported compliance rates at different levels: when participants had taken all pills (100%), nearly all (defined as more than 80%), most of the pills (between 50 and 80%), less than half of the pills (that is, less than 50%), undetermined (but not all) and none of the pills.

For instance, in the study by Park, 2012 [49], compliance proportions were lower than in the other studies in both treatment arms: 72% and 58% with SEQ and STT, respectively. In the study by Aminian, 2010 [71], compliance was reported as 100% in both treatment arms.

### 2.6. Sensitivity Analysis

#### 2.6.1. Risk of Bias

We conducted sensitivity analyses on the ‘Risk of bias’ items assessed during the review process in order to see whether our findings were robust. Table 1 summarizes the risk difference in the overall eradication proportion when studies categorized as ‘unclear’ or ‘high risk’ for each domain were excluded from the overall meta-analysis.

We compared the different RDs with the overall pooled RD of 0.08 with a 95% CI from 0.06 to 0.10 (Figure 4): when studies with poor allocation concealment were excluded, the absolute risk of one treatment over the other was reduced (*), whereas the absolute risk increased when we excluded the poorest quality studies on the blinding item (**).

Also, the differences between treatment arms remained significant in the overall analysis. The ‘Risk of bias’ items therefore do not appear to influence the overall results when we compare SEQ to STT.

#### 2.6.2. Year of Publication

Given the strong differences that we found regarding the year of publication, we repeated all subgroup analyses, separating publications by the year published (before or after 2010) (Table 2).

This sensitivity analysis, summarized in the table below, showed effect differences in the subgroup analyses.

In the subgroup analysis, when we eliminated the studies performed after 2010, the overall tendency was toward lower differences between therapies. These results remained statistically significant in all subgroup meta-analyses, except for the baseline medical condition, nitroimidazole resistance and dual antibiotic resistance.

In the subgroup analysis by baseline medical condition, the non-significant tendencies toward the superiority of SEQ compared to STT, in both NUD and PUD participants, found using all time-span studies (Figure S2), were reduced and nearly eliminated in studies performed after 2010.

For the length of the STT regimen, the previously reported benefit of SEQ when compared to 10-day STT (Figure 9) could not be demonstrated in the most recent studies (2010 onwards), in which the efficacy of SEQ showed a marginally significant statistical advantage, but it was potentially equivalent to that of 10-day STT at a clinical level.

#### 2.6.3. Length of STT

As previously mentioned, the length of the regimen is a major factor affecting the efficacy of antibiotic treatments, especially in the case of *H. pylori*. As shown in our length-dependent subgroup analysis, the differences between SEQ and STT are reduced the longer the STT regimen is prescribed. Since STT is usually recommended as a 10-day regimen, the same number of days of SEQ is given. For these reasons and to try and maintain fair comparisons, we confined our sensitivity analyses to those studies comparing arms lasting 10 days (Table 3).

## 3. Discussion

Several treatments have been suggested for *H. pylori* infection and have been discussed in the literature. Despite the large number of studies performed in the last two decades, no agreement in the optimal empiric first-line eradication regimen has yet been reached. However, in recent years, the efficacy of both a susceptibility-based triple therapy as well as the empirical bismuth quadruple therapy with metronidazole and tetracycline appear to provide encouraging and optimal results above the 90% threshold [19,106,107,108].

There could be many explanations, but mainly efficacy, cost and ease of administration of drugs, as well as bacterial antibiotic resistance, have been reported among current challenges that need to be overcome.

### 3.1. Summary of Main Results

Our main objective was to assess and compare the efficacy of 10-day SEQ versus STT from available published RCTs. The secondary objective was to compare the incidence of adverse events with both regimens.

The screening and full-text assessment of citations resulting from both the electronic and manual searches yielded 69 included RCTs. All studies addressed treatment and compared 10-day SEQ versus 7-, 10- or 14-day STT.

From the included studies, 13 (19%) were published as abstracts from congresses or conferences; the sensitivity analyses showed that no effect modification was associated with the format of the publication, nor with the quality of items assessed for all included studies. This ensures the robustness of the findings in this systematic review.

Among the other subgroup analyses, 25% of the studies were published in Italy (n = 11), and among those, eight were published before 2010. Many others were published after 2010 (n = 61), with very little evidence addressing experience with children (n = 7). Efficacy rates were only provided by pre-treatment antibiotic susceptibility in 13 studies, despite the fact that nearly all of the studies commented on the antimicrobial resistance.

Overall, the efficacy of 10-day SEQ was higher than treatment with 7-day, 10-day and 14-day STT; however, prescribing longer STT reported a reduced risk difference as compared to SEQ, showing only a marginally significant advantage of SEQ when compared to the 14-day STT regimen. Moreover, the alleged superiority of SEQ versus 10-day STT was reduced when only recent studies (after 2009) were evaluated.

### 3.2. Overall Efficacy of SEQ versus STT

Our efficacy endpoint of interest was the *H. pylori* ITT eradication proportion. From the 69 included studies covering 19,661 participants, the overall meta-analysis showed a significantly higher efficacy for 10-day SEQ over the 7-, 10 and 14-day STT. The traditional approach to *H. pylori* infection therapy differs from other infectious diseases. While most bacterial infectious diseases use antimicrobial susceptibility testing to find an appropriate therapy, achieving over 95% reliable cure with the first course, *H. pylori* therapy development faces challenges in attaining such high cure rates [109]. In the absence of routine antibiotic susceptibility testing, current guidelines, like the Maastricht VI/Florence consensus report [2], suggest empirical antimicrobial therapy with >90% efficacy. Any therapy with an intention-to-treat efficacy below 90% has been deemed as poor [17]. And thus, both SEQ and STT provide lower rates (83% versus 75%, respectively) than the optimal required eradication levels (≥90%).

Our findings also showed that the efficacy of both regimens is decreasing over time and are, at present, unacceptable.

The lack of optimal treatment effect has been mainly attributed to bacterial antibiotic resistance. The efficacy of SEQ was less affected by clarithromycin resistance (−19% eradication) than STT (−34% eradication), which might indicate a beneficial effect of using SEQ versus STT in those areas in which clarithromycin resistance is high (>15–20%). Nevertheless, it is essential to highlight that the efficacy of both treatment approaches remained below optimal levels in the presence of clarithromycin bacterial resistance.

Moreover, previous studies have linked the success or failure of antibiotic regimens to various factors, including the number of antibiotics used, poor compliance, type of underlying disease such as PUD or NUD, shorter versus longer STT duration (7 versus 10 versus 14 days), drug-related AEs, PPI type and dosage, previous stomach bacterial load, bacterial virulence (Cag A status), tobacco use, age of the population, geographical region or any other variable that could predict or influence the treatment outcome [110].

We therefore decided to review each of the above variables that were suggested to potentially affect the efficacy of the therapeutical regimen.

### 3.3. Subgroup Analyses: Variables Influencing Efficacy of Both Treatments

#### 3.3.1. Geographic Region

A previous review [32] showed that almost all studies comparing SEQ and STT therapies were performed in Italy, contributing to a lack of validation of findings in other settings. This limitation has been overcome in our present meta-analysis, with 11 studies performed in Italy and all of them showing a significant and clear advantage of SEQ over STT. The majority of studies from other European countries also identified this advantage, although with lower differences in eradication between arms.

The advantage of SEQ was also observed with lower risk differences in Asia and Africa; but STT offered higher eradication proportions versus SEQ in Latin America. As others have already noted, the geographical location could potentially serve as a surrogate factor for a specific pattern of efficacy or resistance rather than directly predicting the efficacy outcome [26,111].

#### 3.3.2. Publication Date

We noted a trend toward a lower efficacy for both STT and SEQ in studies published after the year 2010 (Figure 4 and Figure 5).

Published studies on the topic argue that antibiotic resistance might be one of the most relevant factors mediating the trend of decreased efficacy of treatments over time, and a growing increase in clarithromycin resistance could explain the lower efficacy for both regimens. It is important to mention that if we consider the most recent publications (2010 onwards), we found just marginal differences when comparing SEQ with STT when the latter was used for 10 or 14 days.

#### 3.3.3. Effect Modifiers over Time

Based on the outcomes of this meta-analysis, we could not determine the reasons behind the higher treatment efficacy observed in studies published before 2010 following SEQ (93%) compared with those published after 2010 (80%). This finding could depend on the modulating effect of either the geographic region or on some unevaluated variables associated with the publication date of the included studies, such as an increase in resistance proportions of the strains, migrant population, etc.

As mentioned, only Italian studies were published before the year 2010, and treatment success or failure was measured by these published studies only; factors other than the publication date related to the Italian setting may contribute to the observed change in efficacy over time. Another major effect modifier is the length of STT, which must clearly be taken into consideration in sensitivity analyses.

#### 3.3.4. Age of the Population

Seven RCTs assessed SEQ versus STT in children. Treatment with SEQ was more beneficial than with STT (78% versus 67%), but lower than in the adult population (82% versus 75%, respectively). Data from previous meta-analyses showed similar results [31,112,113], although as for adults, eradication rates with SEQ in children did not achieve the desired level of success.

#### 3.3.5. Medical Condition

The findings of our review suggest that the eradication proportion following SEQ was similar for NUD and PUD participants (84% and 83%, respectively), and that the previously reported differences for STT were not demonstrated in this review (76% versus 77%). Additionally, marginally significant differences were shown between treatment groups in PUD patients, and only a statistical advantage of SEQ in NUD participants was found compared to STT. However, a tendency toward an increased benefit of SEQ over STT was observed in both subgroups of participants, but this apparent advantage was not noted in studies after 2010.

Previous studies [30,35] have also reported the fact that eradication proportions in both PUD and NUD participants following SEQ were similar, suggesting that the SEQ scheme might overcome differences in participants’ baseline medical conditions in a similar manner, or that the underlying disease itself is not a moderator or a predictor of the treatment outcome. These same findings have been also recently confirmed by means of mixed effects logistic regression analysis, where the medical baseline condition (that is, NUD versus PUD), as independent factor, was not significantly associated with higher eradication rate in those receiving SEQ [19].

#### 3.3.6. STT Length

In order to support and reinforce the curative effect of STT, some studies focused on investigating treatment duration. It has been suggested that an extended treatment period, such as prolonging STT to 14 days, may yield heightened efficacy [19,114,115,116,117].

In our review, 10-day SEQ was more effective than 7-, 10- and 14-day STT. We found just marginal differences in efficacy between 10-day SEQ and STT lasting 14 days. Also, the sensitivity analysis supported the superiority of SEQ over 10-day STT in studies published from 2010 onwards, even though there was a tendency toward a reduced RD between therapies.

#### 3.3.7. Acid Inhibition with PPIs

The efficacy of SEQ was consistently superior to STT across various PPI doses. However, this advantage became less distinct and non-significant when employing high-potency inhibition, such as double-dose PPIs. Notably, both SEQ and STT exhibited a trend toward smaller differences in efficacy when the PPI dose was more potent (RD was 0.19 for low inhibition, 0.08 for standard and 0.03 for high).

When including only studies where SEQ and STT were both given for 10 days, the alleged benefit offered by SEQ was reduced to marginal significance in the studies using standard acid inhibition. In studies utilizing PPI for high acid inhibition, this advantage shifted, yielding a better outcome with STT (RD of −0.01). Consistent with this, recent reports highlight the advantage of increasing the potency of acid inhibition in STT. High doses of PPIs have been shown to have a significant impact on the STT cure rate [19].

#### 3.3.8. Bacterial Antibiotic Resistance

Eradication within antimicrobial-resistant strains was reported in only 13 studies. This constitutes a significant limitation of our review, stemming from the absence of reliable, consistent and up-to-date information on the prevalence of antibiotic susceptibility and resistance within the included RCTs.

Antimicrobial resistance is regarded as the primary factor accountable for the diminished efficacy of STT and the declining eradication rates observed over time for SEQ [22,118,119,120].

In our review, SEQ demonstrated significant superiority over STT exclusively among participants with bacterial resistance to clarithromycin. This advantage became even more pronounced when both treatments were administered for the same duration. STT seems, in any event, to be more affected by resistance to clarithromycin (−34% in efficacy) than SEQ (−19%).

The benefit of SEQ over STT was not demonstrated for nitroimidazole or dual-resistant strains. It is important to mention that efficacy for nitroimidazole-resistant strains seems to be higher than the overall analysis, both for SEQ (83% versus 82%) and for STT (82% versus 75%). This counterintuitive improvement can be attributed to the fluctuation in efficacy observed in studies reporting eradication based on antimicrobial resistance. If we consider only studies reporting efficacy due to antimicrobial resistance, the overall eradication is 73% for SEQ and 65% for STT.

Dual resistance had a strong impact on both SEQ and STT, which showed efficacies of 62% and 54%, respectively. This tendency toward the superiority (+8%) of SEQ treatment in dual-resistant strains was reversed when we looked at treatment arms lasting the same number of days (10-day STT), in which 10-day STT offered higher efficacy (+8%) than SEQ.

### 3.4. Safety

Safety was assessed through the incidence of AEs in the included studies. The main category reported was gastrointestinal distress, such as abdominal pain, diarrhea, nausea, glossitis and vomiting.

From the studies addressing tolerance and compliance, the overall incidence of AEs with SEQ and STT was reported to be similar (26% and 25%).

Our findings support data from previous meta-analyses [29,31,32], where AEs as well as compliance were found to be comparable between both regimens. Similar results were reported in a recent publication [121], as part of the Hp-EuReg, evaluating the frequency, type, intensity and duration of AEs and their impact on compliance among the most frequently used first-line treatments in Europe (including STT and SEQ).

### 3.5. Overall Completeness and Applicability of Evidence

The included RCTs notably lack a systematic assessment of antibiotic susceptibility or bacterial resistance. The RCTs failed to systematically report eradication across groups of participants with different underlying diseases (PUD and NUD, mainly). Moreover, there is a scarcity of studies focusing on children. Nearly 30% of the studies did not systematically report data on safety, compliance or withdrawals attributed to treatment side effects. These limitations compromise the comprehensiveness and, ultimately, the generalizability of the evidence to broader populations infected with *H. pylori*.

Despite these limitations, the substantial number of included studies proved sufficient to address the main objective and cover the relevant interventions, participant, and outcomes. The results were validated through prior research, significantly contributing to informing clinical practice and paving the way for further evidence-based research.

Factors influencing the relative efficacy between treatments, including factors like resistance, region, publication year, treatment duration, etc., should be thoroughly considered by clinicians when choosing between these two regimens. It is essential to underscore the need for caution in interpreting subgroup analyses; although a higher risk difference in certain analyses might suggest stronger support for SEQ, it does not necessarily imply an enhancement in SEQ’s efficacy beyond its overall efficacy, and therefore, it does not mean that SEQ should be the treatment of choice in that context. To illustrate this fact, despite the highest RD being observed for clarithromycin-resistant strains, the actual efficacy in that specific context for SEQ was significantly lower than the overall efficacy for SEQ. While SEQ may present notably improved results compared to STT, this treatment still falls short in terms of optimal outcomes, and if available, clinicians should consider pursuing alternative treatments, such as the empirical bismuth quadruple therapy [106,107].

### 3.6. Quality of the Evidence

The included studies were of mixed quality. Usually, randomization was not preserved at the allocation or concealment levels, and sequence generation was inadequate in 36% of the studies. Outcomes based on the length of STT or the rate of AEs were categorized as high quality; however, we downgraded the quality of the evidence for outcomes of the following factors: publication date (moderate quality), geographic region (low quality) and antibiotic resistance (very low quality). The results for these outcomes should therefore be interpreted cautiously.

### 3.7. Intention-to-Treat Reporting

All analyses were based on risk differences using the ITT approach. For the meta-analysis, ITT eradication was based on the study authors’ statements; that is, all patients after randomization were accounted for in the analysis [122]. For our review, complete outcome data were available in all included studies except for three. Firstly, in Lopez-Román, 2011 [74], Wu, 2011 [60], and Choi, 2019 [101], the number of participants randomly assigned to each of the treatment arms were not provided, so we had to estimate the ratio specifying the number of patients cured over the total number of participants randomized to the treatment arm from the percentage of patients cured. The estimated numbers did not always exactly match the percentages.

Secondly, we observed that although ITT analyses were employed following the given definition, obtaining data on the progression of participants through various phases of the trial proved challenging. In some instances, RCTs failed to provide reliable, comprehensive and consistent definitions of participation proportions within the study flow diagram. Consequently, the proportions of participants assigned to different treatment arms might be responsive to distinct participation definitions. Conversely, some trial authors reported proportions without explicitly specifying the particular participation definition to which they were referring.

### 3.8. Reporting of Baseline Characteristics by Treatment Arm versus Not Reporting Findings by Treatment Arm

In total, 53 studies (77%) failed to report he eradication according to medical condition after treatment with SEQ or STT. Certain studies did not specify the initial medical condition of the participants, while others reported the baseline number of patients with either NUD or PUD but did not furnish details on the *H. pylori* cure rate by treatment arm. Despite contacting the authors, the mentioned information could not be obtained.

### 3.9. Masking of Personnel and Participants

Most of the studies were not blinded (neither single- nor double-blinded) and this could be construed as considerably reducing their quality. However, as aforementioned in the current systematic review, it is generally accepted that *H. pylori* eradication is not affected by blinding, given that the placebo effect is unlikely to influence the tests conducted to confirm eradication or the bacteria itself, thus its impact on the results is considered minimal. Furthermore, unmasked studies are thought to give a better estimation of the efficacy in clinical practice, as it is feasible that the more complex SEQ regimen may affect compliance and therefore treatment success [32].

### 3.10. Sample Size

For the meta-analysis, larger sample sizes increase our confidence in the estimate. In our review, 31 studies (45%) had a sample size of fewer than 100 patients at randomization in each treatment arm. Post hoc sensitivity analyses did not show an improvement in the overall effect size of SEQ when sample sizes were doubled in each of the arms. This confirmed the robustness of the results of the meta-analysis.

### 3.11. Recommendations, Other Treatments for H. pylori Eradication and Further Research

STT was initially endorsed as a first-line therapy for the eradication of *H. pylori* in several countries [7], although nowadays its use is not recommended unless proved to be effective in some settings [8,20]. Conversely, numerous studies have reported higher efficacy for SEQ, particularly when compared to 7- and 10-day STT. SEQ has shown more promising results, especially among clarithromycin-resistant populations compared to STT, but its efficacy remains suboptimal.

STT can easily be converted into a non-bismuth ‘concomitant’ quadruple therapy by adding nitroimidazole to the regimen. A meta-analysis comparing concomitant and STT indicates that non-bismuth quadruple (concomitant) therapy is an effective, safe and well-tolerated alternative to STT, praised for its simplicity in comparison to SEQ. Notably, most studies evaluating the non-bismuth quadruple concomitant regimen have been conducted in middle- and high-income countries [21].

More recent studies have evaluated the use of non-bismuth quadruple therapies (both SEQ and concomitant regimens) in clinical settings with increased clarithromycin-resistance proportions, and although differences did not reach statistical significance, there was a tendency toward better efficacy with concomitant therapy [60,62,123,124]. When comparing SEQ and concomitant regimens with the same length of treatment and dosage, efficacy is reported to be higher with concomitant regimens than with SEQ [125,126].

The findings from our review clearly indicate that generally, SEQ proved to be a more effective strategy than STT before 2010 across the majority of evaluated settings. However, a more comprehensive assessment is warranted to delve into aspects such as the superior efficacy of 10-day SEQ compared to 14-day STT, the comparison between SEQ and non-bismuth concomitant quadruple therapy, and the efficacy of 14-day SEQ. For instance, in one of the included studies (Liou, 2013 [63]), 10-day SEQ and 14-day SEQ were compared with each other and with STT. The 14-day SEQ yielded higher efficacy than both 10-day SEQ and STT. SEQ treatment for 14 days eradicated more than 90% of *H. pylori* infections. The overall efficacy obtained with 10-day SEQ treatment in our meta-analysis was clearly suboptimal at only 82% overall. Moreover, as with the STT regimen, there was a trend toward a reduction in efficacy of SEQ over the years, which does not bode well for this strategy.

Recognizing the fairness or lack thereof in comparisons is crucial, and relying on outdated assessments (prior to 2010) or using suboptimal regimens as controls (such as the 7-day STT) to inform clinical decisions appears ethically questionable [127]. The tendency toward the evidence supporting improved STT regimens (high acid inhibition; longer treatment durations) [117] should set these improved regimens as the threshold for comparisons. In line with this principle, 10-day sequential therapy (SEQ) has faced challenges in consistently showcasing superior efficacy.

### 3.12. Potential Biases in the Review Process

The trials provided sufficient data on the efficacy in the various treatment arms, although certain studies, particularly those in abstract form, often presented percentages instead of the actual number of patients cured in each regimen. This necessitated basic calculations to estimate the count of patients with successfully eradicated infections. While eradication proportions were straightforwardly derived in these instances, it is essential to acknowledge the potential bias introduced by outcome reporting. The methodology used throughout this systematic review strictly followed Cochrane standards. Three review authors (JPG, BMB and OPN) conducted constant and comprehensive searches of journal and conference databases to ensure that we had identified all published and unpublished trials. It is worth noting that the electronic search was conducted in three stages throughout the review preparation years, resulting in additional efforts to eliminate duplicates and identify distinct published citations for the same study under different first authors’ names. While we do not advocate rerunning searches whenever feasible, as it is a practice generally accepted among review authors, we acknowledge its occasional necessity given the time required to complete the review. Language restrictions were not imposed, and efforts were made to contact authors for data clarification or additional information, although accessibility proved challenging for some within the time constraints.

### 3.13. Agreements and Disagreements with Other Studies or Reviews

Our systematic review supports previous findings supporting that SEQ is more beneficial than STT when given for 7, 10 or 14 days and where antimicrobial resistances are low.

The findings from previously published pooled data analyses [15,26,29,30,31,32,113,128] also found a significantly higher efficacy for 10-day SEQ over STT. Furthermore, substantially decreased eradication (lower than 80%) achieved by triple therapies has been reported in Europe [20,35,129,130], Asia [131], the United Sates [132] and Canada [133].

## 4. Methods

Only parallel group, randomized controlled trials were eligible for inclusion in this review. We included only those trials comparing 10-day SEQ versus STT for *H. pylori* eradication, as defined in the headings below. We excluded studies that were not assessing an *H. pylori* treatment or that focused on other gastrointestinal conditions. We excluded non-randomized studies, case reports, letters, editorials, commentaries and reviews. Abstracts and full-text forms were eligible for inclusion. There were no restrictions by date of publication or by language.

### 4.1. Selection Criteria

Randomized trials were eligible for inclusion if the study population included adults or children diagnosed as positive for *H. pylori* (with at least one confirmatory test) on the basis of rapid urease test (RUT), histology, polymerase chain reaction (PCR) or culture of an endoscopic biopsy sample, or by urea breath test (UBT) or monoclonal stool antigen test. Study participants had to be naïve to *H. pylori* eradication treatment.

We excluded trials in which participants were diagnosed as *H. pylori*-positive solely on the basis of serology, or who had previously been treated with an eradication therapy. Study participants could not present with serious comorbidities such as HIV infection, malignancy, etc.

### 4.2. Types of Interventions

The 10-day SEQ treatment comprised a PPI and amoxicillin 1 g twice daily, both taken orally for the first five days, followed by a PPI twice daily, clarithromycin 500 mg twice daily and a nitroimidazole (tinidazole or metronidazole at either 400 mg or 500 mg) twice daily, all taken orally for the following five days.

We included only trials assessing SEQ treatment lasting 10 days. Studies were subject to exclusion if there were any variations in the intervention schedule regarding the length of the SEQ treatment.

STT consisted of a PPI, clarithromycin 500 mg twice daily and amoxicillin 1 g twice daily, all taken orally and lasting at least seven days.

### 4.3. Types of Outcome Measures

We included all relevant trials, even if they did not report evidence of eradication of *H. pylori* as their primary outcome.

The primary outcome of interest was the reported efficacy, defined as the proportion/rate of *H. pylori* eradication/cure.

Trials were included if they reported the number of participants with *H. pylori* eradication. In cases where percentages were reported instead of actual numbers, we derived the proportion of participants cured from the ITT randomized sample size for each treatment arm. To be included, trials needed to confirm *H. pylori* eradication through methods such as RUT or histology of an endoscopic biopsy sample, UBT, or a monoclonal stool antigen test, at least four weeks after completing the treatment.

Trials relying solely on serology tests or culture assessments were excluded.

Reported incidence of AEs was also included. AE incidence was recorded as the number of participants reporting any type of AE; any gastrointestinal disturbance such as nausea or vomiting; any dermatological problem; any systemic effect (fever, headache or dizziness); or any serious AE.

A serious AE was defined as a significant and medically important event, such as death, life-threatening situations, hospitalization or permanent damage associated with a medical drug. We distinguished between a serious AE and a severe AE, i.e., an intense form of AE that usually incapacitates an individual’s normal life. Reported severe AEs were also collected.

Adherence to treatment was characterized by the extent to which participants conformed to the prescribed treatment guidelines, encompassing factors such as drug type, dosage and treatment duration.

Additionally, we compiled information on the documented proportion of participant withdrawals, elucidated as the number of individuals discontinuing treatment due to adverse events (AEs).

### 4.4. Search Methods for Identification of Studies

#### 4.4.1. Electronic Searches

We conducted bibliographical searches in the Cochrane Central Register of Controlled Trials (CENTRAL) through the Cochrane Library, MEDLINE, EMBASE and CINAHL electronic databases. Please refer to File S2 for detailed search strategies in each of the databases.

We combined search terms to capture two components of the study question: the disease (*H. pylori* infection) and the intervention of interest (the comparison of STT versus SEQ treatment). We used the following combination of terms (all fields): (Helicobacter OR pylori) AND sequential AND (triple OR “standard regimen” OR “standard therapy”).

We ran the electronic search up to July 2021.

#### 4.4.2. Other Sources

We performed additional manual searches of websites using the same syntax as above in order to retrieve additional publications not captured by the electronic searches. The manual search aimed to identify abstracts of RCTs that might not have been published in peer-reviewed journals but only as part of conference proceedings, specialized journals or international congresses such as the International Workshop of the EHMSG, the DDW and the UEGW.

We reviewed each of the abstracts identified as potentially eligible and included only those meeting the inclusion criteria.

We conducted detailed cross-referencing from the bibliographies of the included studies as well as from other systematic reviews in order to identify further relevant trials.

### 4.5. Data Collection and Analysis

Prior to the selection-of-studies phase, most duplicates were automatically removed when studies were imported to the citation manager. We removed the remaining duplicates manually during the first screening phase.

The process of study selection from the retrieved searches comprised two phases. Initially, we screened titles and abstracts (first screening phase) against the inclusion criteria to identify potentially relevant publications. Subsequently, we scrutinized the full papers (second screening phase) of studies identified as potentially eligible during the first screening phase.

For abstracts or articles lacking sufficient detail to meet the inclusion criteria, we reached out to the authors for additional information.

Based on the preferred reporting items for systematic reviews and meta-analyses (PRISMA) approach (www.prisma-statement.org) accessed on 25 October 2023 [34], we developed a schematic diagram to standardize the steps used for the identification and selection of studies. We specified the number of studies considered at each step and the reason for exclusion of each of the excluded studies.

Two review authors (OPN and BM) carried out both the first and second screenings independently, resolving any discrepancies by discussion and consulting a third review author (JPG) for unresolved disagreements.

#### Data Extraction and Management

During the protocol phase, we developed a pre-tested data extraction form to record data from the selected papers. We collected the following fields during the data extraction process:

- The first author’s name; year of publication; country;
- The format of publication (abstract versus journal article); age of the population (adult versus children);
- Medical condition (PUD or NUD or other);
- Number of participants in each treatment group;
- Name, dose and timing of antibiotic administration; length of STT;
- Eradication proportion per treatment regimen (ITT and PP); if only the PP sample was reported, we calculated the ITT sample on the basis of the randomization and dropout information;
- Definition of compliance and the level of compliance in the ITT sample;
- Details of the method of assessment of *H. pylori* infection both before and after treatment;
- Whether the antibiotic sensitivity and resistance were tested before and after eradication; if so, the primary and secondary antibiotic resistance;
- Incidence, type and severity of AEs;
- Study quality: generation of the treatment allocation, concealment of the treatment allocation at randomization, implementation of masking, completeness of follow-up and use of ITT analysis.

We contacted study authors for any missing data.

Two review authors (OPN and BM) carried out data extraction independently, resolving any discrepancies by discussion and consulting a third review author (JPG) for unresolved disagreements.

### 4.6. Quality of the Evidence

The assessment of the risk of bias of included studies is detailed in File S3.

We assessed six components of quality following the quality checklist recommended in the *Cochrane Handbook for Systematic Reviews and Interventions* [134]. We evaluated the quality based on the information provided in the published trials, mindful of the risk of overestimating intervention effects in RCTs with inadequate methodological quality [135]. We contacted the authors for any missing information.

Two review authors (OPN and BM) independently assessed the methodological quality of all of the included studies. As in previous phases, we sought the opinion of a third review author (JPG) in cases of disagreement.

### 4.7. Completeness of Follow-Up and Use of Intention-to-Treat (ITT) Analysis

We observed the percentage of participants with missing outcome data and/or excluded from the analysis in each arm of the trial. For the ITT analyses, we assumed that these participants had failed therapy. We stated whether the analysis included all randomized participants, i.e., whether an ITT approach was undertaken.

We recorded the authors’ definitions when they reported an ITT analysis. Due to the varied definitions of ITT used by authors, we favored the most widely accepted definition of the ITT approach. All participants were to be analyzed in the groups to which they were initially randomly assigned, irrespective of whether they met the entry criteria, the treatment they received or any subsequent withdrawal or deviation from the protocol [136].

We included comprehensive data for all randomized participants, considering studies reporting either ITT or PP analysis. In cases where a different ITT approach was used in the study or only PP analysis was reported, we proactively reached out to authors to obtain our preferred ITT analysis approach. These four quality components are part of the key methodological features that are important to the validity and interpretation of included trials as mentioned above [137]. We did not score the quality of the studies and did not exclude studies classified as ‘low quality’. We used the individual quality assessment items to explore heterogeneity. If we found significant heterogeneity between studies (details below), we explored it by using a subgroup analysis with pooled effect size estimates and discussed them when interpreting the results.

Regarding the measures of treatment effect, given that the outcome was common, that is, that ‘*H. pylori* eradication’ was usually expected after treatment, and that the treatment and follow-up themselves were fixed for each arm, the odds ratio (OR) would produce a biased effect estimate. We therefore expressed dichotomous outcomes of individual studies using the RD together with the 95% CI, taking ‘*H. pylori* eradication’ as the primary outcome. The RD describes the difference in the risk of observing an event in the SEQ treatment group versus the STT comparison group, for which a value of 0 indicates that the estimated effects are the same for both interventions [134].

We treated the SEQ arm as the intervention group and the STT arm as the control group.

Regarding any potential unit of analysis issues, we included only standard design, parallel, randomized controlled trials. Our interest was only in the direct comparison between the two treatment regimens (10-day SEQ and 7- to 14-day STT). We did not include multiple groups in a single pair-wise comparison so that the same participant was not used twice in the same analysis.

However, multigroup comparisons are usual across treatment arms in clinical trials. For instance, the ITT population could be randomized into three different treatment arms (or schedules): STT lasting 7 days, STT lasting 14 days and SEQ treatment lasting 10 days. In such cases, for the purpose of the overall analysis, we combined the different arms of the same treatment (i.e., 7-day STT and 14-day STT) by summarizing the number of participants in each arm. Afterwards, we undertook the corresponding subgroup meta-analyses using the separate arms for STT treatment duration.

To evaluate various treatment schemes within the same treatment arm, we employed standard single pair-wise comparisons, as outlined in the subgroup analyses section.

Concerning missing data, we proactively contacted authors to address any incomplete outcome data in the included studies. Participants with missing outcome data (attributed to dropouts or incomplete records) were considered to have failed in achieving eradication for the primary outcome.

### 4.8. Assessment of Heterogeneity

In order to identify the possible diversity in trial characteristics, we analyzed the clinical, methodological and statistical components.

We performed the Chi^2^ test for heterogeneity for each combined analysis, where *p* < 0.10 indicated significant heterogeneity between studies [138]. The *I*^2^ statistic was reported, which quantifies heterogeneity by calculating the percentage of total variation across studies that is due to heterogeneity (an approach that has been endorsed by Cochrane Collaboration). We defined significant heterogeneity as *I*^2^ > 25%, based on the judgement that *I*^2^ values below 25%, 50% and 75% represent low, moderate and high heterogeneity, respectively.

We used graphical methods (forest plots) to complete the Chi^2^ test assessment. When we identified heterogeneity, we investigated the source using additional techniques, such as subgroup analyses or funnel plots, to work out whether particular characteristics of studies were related to the sizes of the treatment effect, according to the *Cochrane Handbook for Systematic Reviews of Intervention* [134].

### 4.9. Assessment of Reporting Biases

To assess publication bias, we checked for funnel plot asymmetry by examining the relationship between the treatment effects and the standard error of the estimate.

We produced funnel plots for the principal outcome for each comparison (plots of RD against the standard error (log of RD)).

### 4.10. Data Synthesis

In order to collate, combine and summarize the information from the included studies, we decided to undertake a quantitative (meta-analytic) approach. If there were insufficient trials (two or fewer) reporting for the same comparison, then we would conduct a qualitative evaluation (narrative).

As the first step for the data synthesis, we present an initial overview of results referring generally to all included studies (Table S1). We give these overall findings in a descriptive fashion, in terms of geographic region, target populations, sample sizes, age of the population, medical condition at the baseline and treatment schedules assessed (description of studies).

The second step in the evidence synthesis consisted of summarizing the information related to the size of the effect for all studies, as well as for each different participant group, comparison or outcome measure undertaken. We also report results from subgroup analyses as well as sensitivity analyses.

We performed a meta-analysis combining the RDs for the individual studies with a global RD using a random effects method for dichotomous outcomes (Mantel-Haenszel). Additional sensitivity analyses were performed to check the robustness of the results [139,140]. We conducted pooled analyses using RevMan Web Version: 4.12.0.

We performed subgroup analyses to identify sources of heterogeneity and report summary estimates of the RD within subgroups of these identified sources.

There are several methods to calculate the NNTB and some have limitations [141,142,143]. Many published meta-analyses do not provide the results or the methods used. In this review, we calculated the NNTB for efficacy and the NNTH for adverse events by using the formula NNT = 1/|RD| [134], where |RD| stands for the absolute value of the risk difference. The NNTB was always reported among those statistically significant comparisons.

We performed pre-planned subgroup analyses, regardless of whether significant heterogeneity was present, for the following factors:

- Geographic region;
- Publication date;
- Age (children versus adults);
- Length of STT (7 versus 10 versus 14 days);
- Type of nitroimidazole (metronidazole versus tinidazole); resistance of each antibiotic;
- Dosing for PPI (SEQ treatment versus STT), where the PPI dosage was categorized in three categories as follows: (1) low-dose PPI ranging between 4.5 and 27 mg of omeprazole equivalents, two times per day (i.e., 20 mg of omeprazole equivalents, two times per day); (2) standard-dose PPI ranging between 32 and 40 mg of omeprazole equivalents, two times per day (i.e., 40 mg of omeprazole equivalents, two times per day) and (3) high-dose PPI ranging between 54 and 128 mg of omeprazole equivalents, two times per day (i.e., 80 mg of omeprazole equivalents, two times per day). These dosage categories were calculated based on the definitions of PPI dosage standardization reported by Graham et al. [144] and Kirchheiner et al [145].
- Type of disease at enrolment (PUD versus NUD).

Further methodological evaluation processes, such as GRADE, and corresponding sensitivity analyses are detailed in File S4.

## 5. Conclusions

### 5.1. Implications for Practice

Our review provides further robust assessments across a much broader range of participants comparing SEQ versus STT than in previously published reviews. The findings show a clear benefit of 10-day SEQ over 7-day STT in treatment-naïve *H. pylori*-infected patients overall. Although 10-day SEQ seemed to achieve higher eradication rates than 10-day STT, this benefit showed a tendency toward lower differences in the most recent studies (from 2010 and later) or when STT was prescribed for 14 days.

This review found that efficacy depended on several factors, including the geographic region of the study, bacterial resistance and the date of the study. For instance, we observed a higher efficacy of SEQ versus STT among patients with clarithromycin-resistant strains. The review indicates that before 2010, the cure rate for SEQ was significantly higher than for STT. We found a reduction in the cure rate over time of both STT and SEQ treatments, with a stronger reduction for SEQ. In fact, in the studies published after 2010, SEQ did not show a significantly higher efficacy than STT when the latter was given for at least 10 days.

However, the cure rate of both treatments was lower than we would expect. Neither SEQ nor STT were able to achieve optimal results, and therefore, the evidence collected and combined in this review does not support the use of SEQ treatment. In summary, at the present time, neither SEQ nor STT regimens can be considered valid alternatives for empiric therapy without susceptibility testing, as they do not achieve optimal efficacy for *H. pylori* eradication.

### 5.2. Implications for Research

Given the results of our meta-analysis, 10-day SEQ has inadequate efficacy to be recommended as an alternative first-line empiric therapy for *H. pylori* infection. More importantly, the efficacy obtained with other suggested treatments, such as non-bismuth quadruple concomitant regimen or bismuth quadruple therapy, should be explored further, especially in low-income countries where the burden of infection is greatest.

Another important aspect would be to evaluate *H. pylori* antibiotic susceptibility patterns and the effects on treatment success.

Safety, compliance and withdrawals due to adverse events were usually under-reported in the included studies and need to be considered more fully and systematically in future primary studies.

## Figures and Tables

**Table 1:** Sensitivity analysis: risk difference in the overall eradication proportion when studies categorized as ‘unclear’ or ‘high risk’ for each domain were excluded from the overall meta-analysis.

Risk of Bias Item	RD (95% CI) in Sensitivity Analysis	Impact on the Overall Eradication
Randomization (n = 2 excluded studies)	0.08 (0.06 to 0.11)	Differences between therapies are still significant
Allocation concealment (n = 43 excluded studies)	♦ when excluding “high” risk (n = 19): 0.07 (0.05 to 0.10) * ♦ when excluding “high” and “unclear” risk: 0.06 (0.03 to 0.10) *	Differences between therapies are still significant
Blinding (n = 60 excluded studies)	♦ when excluding “high risk” (n = 55): 0.08 (0.04 to 0.12) ♦ when excluding high and unclear risk (n = 60): 0.09 (0.03 to 0.14) **	Differences between therapies are still significant
Incomplete outcome data (n = 22 excluded studies)	0.10 (0.07 to 0.12) **	Differences between therapies are still significant
Publication format (n = 13 excluded studies)	0.08 (0.06 to 0.11)	Differences between therapies are still significant

RD: risk difference; CI: confidence interval; n: number of studies. * The absolute risk of one treatment over the other was reduced; ** the absolute risk of one treatment over the other increased.

**Table 2:** Sensitivity analysis: risk difference in the different subgroups of analysis when studies performed before 2010 were excluded from the meta-analysis.

Subgroups by Year of Publication (after 2010)	RD (95% CI) in Sensitivity Analyses	Impact on the Overall Eradication
Eradication proportion	0.07 (0.05 to 0.09)	Tendency toward lower/no differences between therapies
Geographic region (Europe)	0.14 (0.10 to 0.18) (Europe) 0.07 (0.05 to 0.10) (Total)	Tendency toward lower/no differences between therapies
Age of the population	0.07 (0.04 to 0.09) (Adults) 0.07 (0.05 to 0.09) (Total)	Tendency toward lower/no differences between therapies
Baseline medical condition—PUD participants	0.02 (−0.07 to 0.12)	Tendency toward lower/no differences between therapies
Baseline medical condition—NUD participants	0.03 (−0.04 to 0.09)	Tendency toward lower/no differences between therapies
Length of STT regimen—7 days	0.11 (0.09 to 0.14)	Tendency toward lower/no differences between therapies
Length of STT regimen—10 days	0.04 (0.00 to 0.07)	Tendency toward lower/no differences between therapies
Metronidazole type (tinidazole)	0.09 (0.05 to 0.14)	Tendency toward lower/no differences between therapies
PPI acid inhibition (standard dose)	0.07 (0.04 to 0.09)	Tendency toward lower/no differences between therapies
Bacterial antibiotic resistance (clarithromycin)	0.23 (0.07 to 0.39)	Tendency toward lower/no differences between therapies
Bacterial antibiotic resistance (nitroimidazole)	−0.02 (−0.07 to 0.04)	Tendency toward lower/no differences between therapies
Bacterial antibiotic resistance (dual)	0.10 (−0.00 to 0.20)	Tendency toward lower/no differences between therapies

RD: risk difference; CI: confidence interval; n: number of studies. PUD: peptic ulcer disease; NUD: non-ulcer disease; PPI: proton pump inhibitor.

**Table 3:** Sensitivity analysis: risk difference in the different subgroups of analysis when only studies including 10-day standard triple therapy where included the meta-analysis.

Subgroups by STT Length of 10 Days	RD (95% CI) in Sensitivity Analyses	Impact on the Overall Eradication
Baseline medical condition—PUD participants	0.02 (−0.10 to 0.13)	Tendency toward lower/no differences between therapies
Baseline medical condition—NUD participants	0.08 (−0.02 to 0.19)	Tendency toward higher differences between therapies
Clarithromycin resistance	0.56 (0.36 to 0.75)	Tendency toward higher differences between therapies
Nitroimidazole resistance	0.01 (−0.08 to 0.11)	Tendency toward lower/no differences between therapies
Dual resistance	−0.12 (−0.32 to 0.08)	Tendency shift toward higher efficacy with STT
PPI dose—standard acid inhibition	0.06 (0.01 to 0.10)	Tendency toward lower/no differences between therapies
PPI dose—high acid inhibition	−0.01 (−0.14 to 0.11)	Tendency shift toward lower/no differences between therapies
Geographic region—Latin America	−0.04 (−0.12 to 0.04)	Tendency toward lower/no differences between therapies
Geographic region—Africa	0.00 (−0.19 to 0.19)	Tendency toward lower/no differences between therapies
Geographic region—Asia	0.03 (−0.02 to 0.08)	Tendency toward lower/no differences between therapies
Nitroimidazole type—metronidazole	0.05 (−0.00 to 0.09)	Tendency toward lower/no differences between therapies

STT: standard triple therapy; RD: risk difference; CI: confidence interval; n: number of studies. PUD: peptic ulcer disease; NUD: non-ulcer disease; PPI: proton pump inhibitor. Dosing for PPI: standard-dose PPI ranging between 32 and 40 mg of omeprazole equivalents, two times per day; high-dose PPI ranging between 54 and 128 mg of omeprazole equivalents, two times per day.

## Supplementary Materials

The following supporting information can be downloaded at: https://www.mdpi.com/article/10.3390/antibiotics13020136/s1, Figure S1: Forest plot comparison: sequential therapy versus standard triple therapy. Age of the population; Figure S2: Forest plot comparison: sequential therapy versus standard triple therapy. Medical condition; Figure S3: Forest plot comparison: sequential therapy versus standard triple therapy. Nitroimidazole type; Figure S4: Forest plot comparison: sequential therapy versus standard triple therapy. Proton pump inhibitors for acid inhibition; Figure S5: Forest plot comparison: sequential therapy versus standard triple therapy. Bacterial antibiotic resistance; Figure S6: Forest plot comparison: sequential therapy versus standard triple therapy. Adverse events rate; Table S1: Characteristics of included studies; Table S2: Summary of findings table; File S1: Results: risk of bias of the included studies;. File S2: Detailed search strategies in each of the databases; File S3: Methods: risk of bias assessment; File S4: Quality of the body of evidence (GRADE methodology).
