# Supplementary material for: Sequential versus Standard Triple Therapy for First-Line Helicobacter pylori Eradication: An Update
Source: Antibiotics (Basel). 2024 Jan 30;13(2):136. doi: 10.3390/antibiotics13020136 (PMC10885881; doi:10.3390/antibiotics13020136)
Supplement: Supplementary file 1 [file antibiotics-13-00136-s001.zip › antibiotics-2800062-supplementary material.pdf]

Results

Study or Subgroup	Sequential Events	Sequential Total	Standard Events	Standard Total	Weight	Risk Difference M-H, Random, 95% CI
**1.4.1 Children**						
Ali Habib HS 2013	4	9	5	9	0.2%	-0.11 [-0.57 , 0.35]
Laving 2013	22	52	22	52	0.8%	0.00 [-0.19 , 0.19]
Zhu 2017	86	102	81	100	1.5%	0.03 [-0.07 , 0.14]
Bontems 2011	68	83	59	82	1.3%	0.10 [-0.03 , 0.23]
Huang 2013	96	118	157	242	1.7%	0.16 [0.07 , 0.26]
Lu 2010	36	40	26	36	0.9%	0.18 [0.00 , 0.35]
Albrecht 2011	45	52	35	51	1.0%	0.18 [0.02 , 0.34]
**Subtotal (95% CI)**		**456**		**572**	**7.4%**	**0.11 [0.05 , 0.17]**
Total events:	357		385			
Heterogeneity: Tau² = 0.00; Chi² = 6.97, df = 6 (P = 0.32); I² = 14%						
Test for overall effect: Z = 3.68 (P = 0.0002)						
**1.4.2 Adults**						
Lopez-Román 2011	27	41	33	41	0.8%	-0.15 [-0.34 , 0.04]
Aminian 2010	86	107	97	107	1.7%	-0.10 [-0.20 , -0.01]
Alsohaibani 2015	58	115	69	117	1.3%	-0.09 [-0.21 , 0.04]
Lee 2014	74	115	80	111	1.4%	-0.08 [-0.20 , 0.04]
Lee 2015	109	170	119	170	1.6%	-0.06 [-0.16 , 0.04]
Zhou 2014	93	140	101	140	1.5%	-0.06 [-0.17 , 0.05]
Greenberg 2011	372	486	401	488	2.2%	-0.06 [-0.11 , -0.01]
Warrington 2016	68	85	72	86	1.4%	-0.04 [-0.15 , 0.08]
Byambajav 2019	61	90	64	90	1.2%	-0.03 [-0.17 , 0.10]
Eisig 2014	43	50	43	50	1.2%	0.00 [-0.14 , 0.14]
Wu 2011	46	102	46	102	1.2%	0.00 [-0.14 , 0.14]
Yan 2011	185	246	220	293	1.9%	0.00 [-0.07 , 0.07]
Ennkaa 2018	36	71	68	135	1.1%	0.00 [-0.14 , 0.15]
Rakici 2014	146	172	144	171	1.9%	0.01 [-0.07 , 0.08]
Choi 2012	87	115	86	115	1.5%	0.01 [-0.10 , 0.12]
Ang 2015	130	154	129	155	1.8%	0.01 [-0.07 , 0.09]
Liou 2016	567	650	557	650	2.3%	0.02 [-0.02 , 0.05]
Liou 2014	367	416	367	424	2.2%	0.02 [-0.03 , 0.06]
Liou 2013	261	300	247	300	2.1%	0.05 [-0.01 , 0.10]
Yang 2015	128	150	121	150	1.8%	0.05 [-0.04 , 0.13]
Auesomwang 2018	51	60	48	60	1.2%	0.05 [-0.09 , 0.19]
Jeon 2013	60	77	58	81	1.2%	0.06 [-0.07 , 0.20]
Gao 2010	64	72	58	71	1.4%	0.07 [-0.04 , 0.19]
Kim 2017	191	223	181	231	1.9%	0.07 [0.00 , 0.14]
Preda 2017	37	49	32	47	0.9%	0.07 [-0.11 , 0.25]
Hsu 2014	91	102	84	103	1.6%	0.08 [-0.02 , 0.17]
Phiphatpatthamaamphan 2016	47	50	43	50	1.4%	0.08 [-0.04 , 0.20]
Chung 2016	120	170	107	171	1.6%	0.08 [-0.02 , 0.18]
Shrestha 2020	18	31	16	32	0.6%	0.08 [-0.16 , 0.33]
Kim 2019	255	300	229	300	2.0%	0.09 [0.02 , 0.15]
Boal Carvalho 2017	25	29	24	31	0.8%	0.09 [-0.11 , 0.28]
Choi 2019	255	300	228	300	2.0%	0.09 [0.03 , 0.15]
Nasa 2013	98	111	95	120	1.7%	0.09 [-0.00 , 0.19]
Tepes 2016	113	120	97	116	1.8%	0.11 [0.03 , 0.18]
Ravarian 2018	58	66	51	66	1.3%	0.11 [-0.02 , 0.23]
Kim 2011	176	205	153	204	1.9%	0.11 [0.03 , 0.18]
Ozturk 2017	34	47	30	49	0.8%	0.11 [-0.08 , 0.30]
Kim 2016	252	306	209	295	2.0%	0.12 [0.05 , 0.18]
Vaira 2007	134	150	116	150	1.8%	0.12 [0.04 , 0.20]
Molina-Infante 2010	88	115	74	115	1.4%	0.12 [0.00 , 0.24]
Haider 2015	29	42	25	44	0.7%	0.12 [-0.08 , 0.32]
Kim 2019b	288	377	241	377	2.0%	0.12 [0.06 , 0.19]
Javid 2013	105	138	83	134	1.5%	0.14 [0.03 , 0.25]
Zullo 2005	84	89	72	90	1.6%	0.14 [0.05 , 0.24]
Focareta 2003	166	174	149	184	2.0%	0.14 [0.08 , 0.21]
De Francesco 2004a	43	45	42	52	1.3%	0.15 [0.02 , 0.27]
Focareta 2002	90	94	75	93	1.7%	0.15 [0.06 , 0.24]
Park 2012	126	162	102	164	1.6%	0.16 [0.06 , 0.25]
Lahbabi 2013	98	104	90	115	1.7%	0.16 [0.07 , 0.25]
Oh 2012	92	116	82	130	1.5%	0.16 [0.05 , 0.27]
Seddik 2013	116	140	93	141	1.6%	0.17 [0.07 , 0.27]
Moosavi 2019	70	77	54	73	1.4%	0.17 [0.05 , 0.29]
Sezikli 2018	24	35	18	35	0.6%	0.17 [-0.05 , 0.40]
Chung 2012	60	79	47	80	1.1%	0.17 [0.03 , 0.32]
Zullo 2003	481	522	389	527	2.2%	0.18 [0.14 , 0.23]
Scaccianoce 2006	68	72	53	70	1.4%	0.19 [0.07 , 0.30]
Gatta 2011	124	131	81	108	1.7%	0.20 [0.11 , 0.29]
Paoluzi 2010	78	90	59	90	1.4%	0.21 [0.09 , 0.33]
Harmandar 2017	37	40	28	40	1.0%	0.23 [0.06 , 0.39]
De Francesco 2004b	110	116	82	115	1.7%	0.24 [0.14 , 0.33]
Farhoud 2020	27	34	19	34	0.7%	0.24 [0.02 , 0.45]
Franceschi 2011	36	50	24	50	0.8%	0.24 [0.05 , 0.43]
**Subtotal (95% CI)**		**9085**		**9233**	**92.6%**	**0.08 [0.06 , 0.10]**
Total events:	7463		6905			
Heterogeneity: Tau² = 0.00; Chi² = 203.22, df = 61 (P < 0.00001); I² = 70%						
Test for overall effect: Z = 7.17 (P < 0.00001)						
**Total (95% CI)**		**9541**		**9805**	**100.0%**	**0.08 [0.06 , 0.10]**
Total events:	7820		7290			
Heterogeneity: Tau² = 0.00; Chi² = 211.83, df = 68 (P < 0.00001); I² = 68%						
Test for overall effect: Z = 7.71 (P < 0.00001)						
Test for subgroup differences: Chi² = 0.76, df = 1 (P = 0.38), I² = 0%						

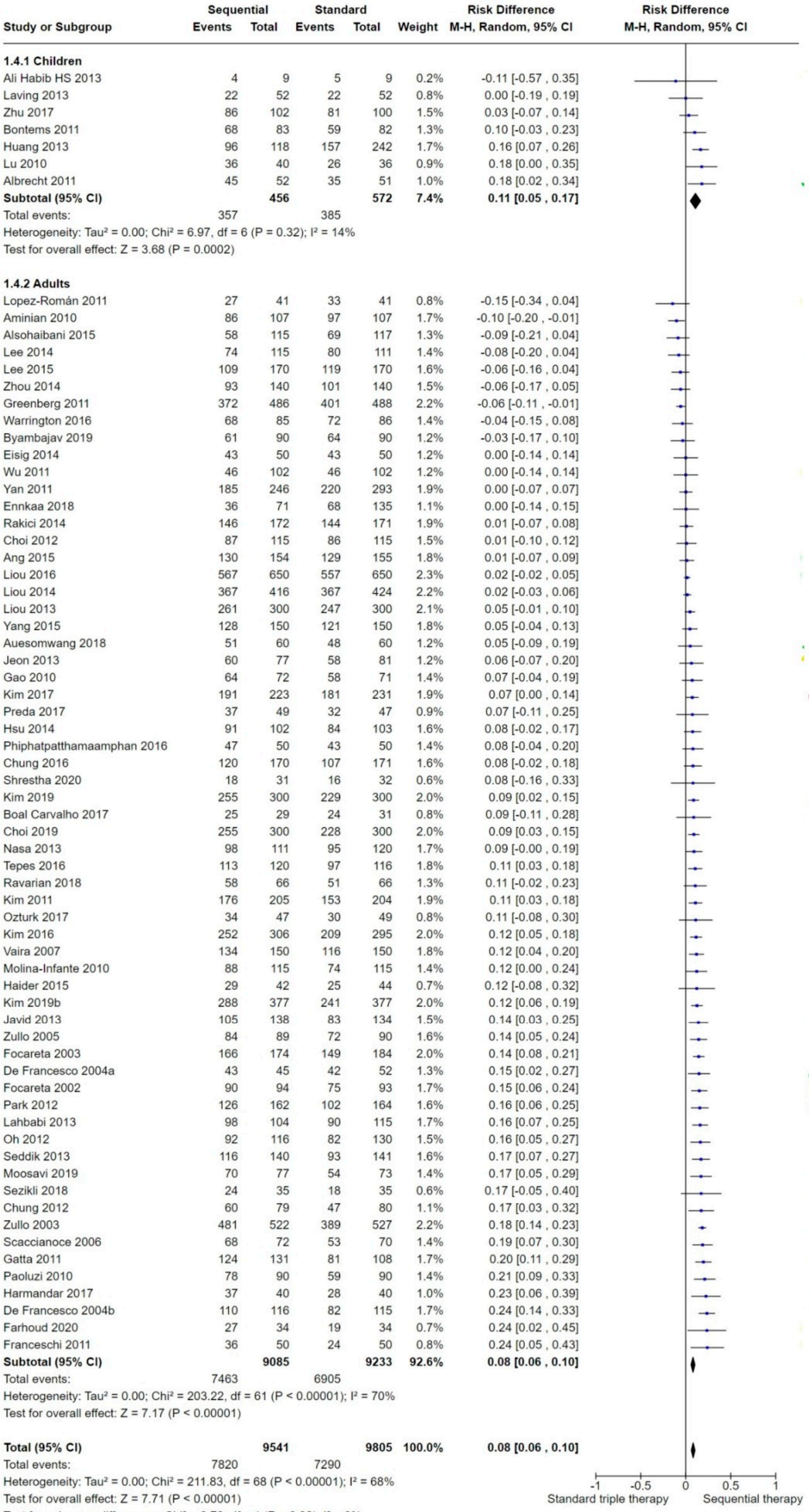

Figure S1. Forest plot comparison: sequential therapy versus standard triple therapy. Age of the population. M-H: Mantel–Haenszel; CI: confidence interval. Risk of bias legend: A: random sequence generation (selection bias); B: allocation concealment (selection bias); C: selective reporting(reporting bias)..

Study or Subgroup	Sequential Events	Sequential Total	Standard Events	Standard Total	Weight	Risk Difference M-H, Random, 95% CI
**1.5.1 PUD only**						
Zullo 2003	124	128	101	135	5.5%	0.22 [0.14 , 0.30]
De Francesco 2004b	36	37	72	82	5.3%	0.09 [0.01 , 0.18]
Zullo 2005	84	89	72	90	5.2%	0.14 [0.05 , 0.24]
Molina-Infante 2010	16	20	13	18	2.2%	0.08 [-0.19 , 0.35]
Kim 2011	56	62	53	72	4.5%	0.17 [0.04 , 0.29]
Chung 2012	47	80	60	79	4.2%	-0.17 [-0.32 , -0.03]
Javid 2013	105	138	83	134	4.9%	0.14 [0.03 , 0.25]
Liou 2013	180	199	161	179	5.9%	0.01 [-0.05 , 0.07]
Zhou 2014	93	140	101	140	4.9%	-0.06 [-0.17 , 0.05]
**Subtotal (95% CI)**		**893**		**929**	**42.6%**	**0.07 [-0.01 , 0.15]**
Total events:	741		716			
Heterogeneity: Tau² = 0.01; Chi² = 41.68, df = 8 (P < 0.00001); I² = 81%						
Test for overall effect: Z = 1.83 (P = 0.07)						
**1.5.2 NUD only**						
Zullo 2003	357	394	288	392	6.0%	0.17 [0.12 , 0.22]
De Francesco 2004b	74	79	103	149	5.3%	0.25 [0.15 , 0.34]
Scaccianoce 2006	68	72	111	141	5.4%	0.16 [0.07 , 0.24]
Aminian 2010	86	107	97	107	5.2%	-0.10 [-0.20 , -0.01]
Molina-Infante 2010	48	67	43	72	3.9%	0.12 [-0.04 , 0.28]
Greenberg 2011	91	127	104	125	5.0%	-0.12 [-0.22 , -0.01]
Kim 2011	120	143	100	132	5.2%	0.08 [-0.01 , 0.18]
Liou 2013	78	86	82	100	5.1%	0.09 [-0.01 , 0.18]
Phiphatpatthamaamphan 2016	47	50	43	50	4.7%	0.08 [-0.04 , 0.20]
Sezikli 2018	24	35	18	35	2.8%	0.17 [-0.05 , 0.40]
Auesomwang 2018	51	60	48	60	4.3%	0.05 [-0.09 , 0.19]
Byambajav 2019	61	90	64	90	4.4%	-0.03 [-0.17 , 0.10]
**Subtotal (95% CI)**		**1310**		**1453**	**57.4%**	**0.07 [0.01 , 0.14]**
Total events:	1105		1101			
Heterogeneity: Tau² = 0.01; Chi² = 59.04, df = 11 (P < 0.00001); I² = 81%						
Test for overall effect: Z = 2.15 (P = 0.03)						
**Total (95% CI)**		**2203**		**2382**	**100.0%**	**0.07 [0.02 , 0.12]**
Total events:	1846		1817			
Heterogeneity: Tau² = 0.01; Chi² = 101.28, df = 20 (P < 0.00001); I² = 80%						
Test for overall effect: Z = 2.92 (P = 0.003)						
Test for subgroup differences: Chi² = 0.00, df = 1 (P = 0.97), I² = 0%						

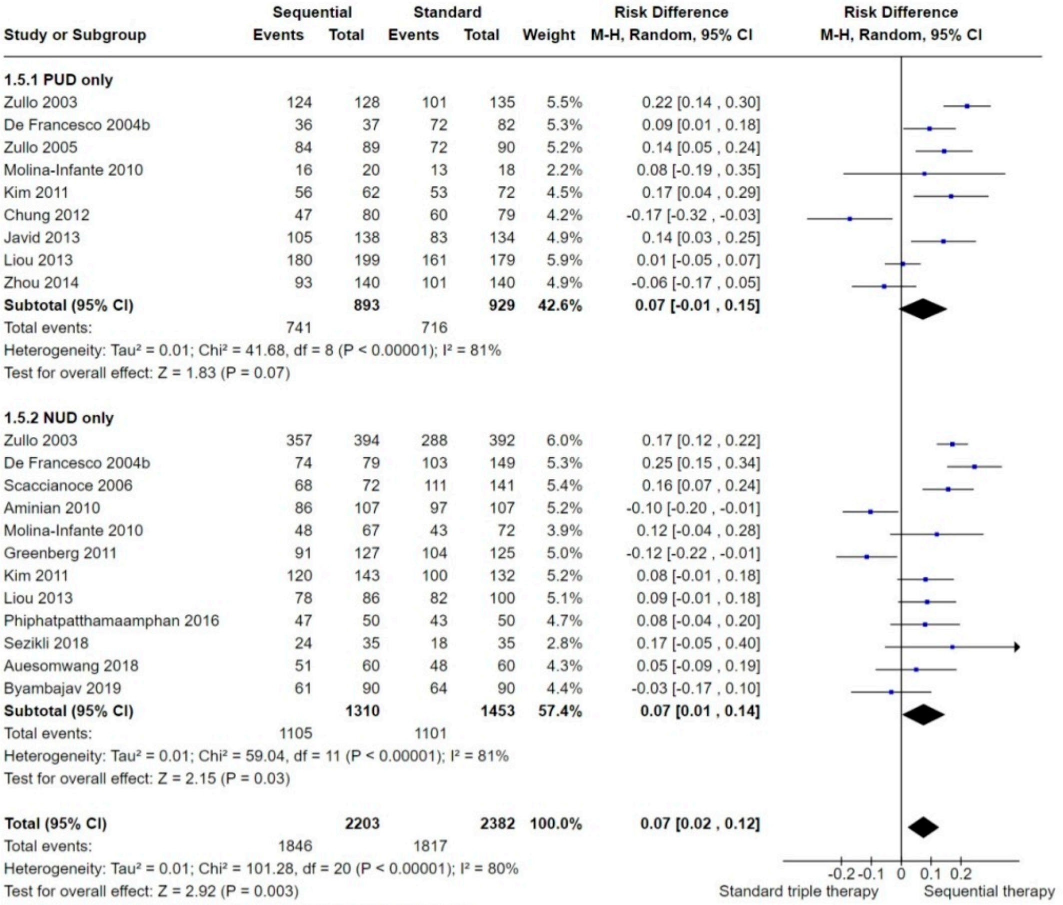

Figure S2. Forest plot comparison: sequential therapy versus standard triple therapy. Medical condition. M-H: Mantel–Haenszel; CI: confidence interval.

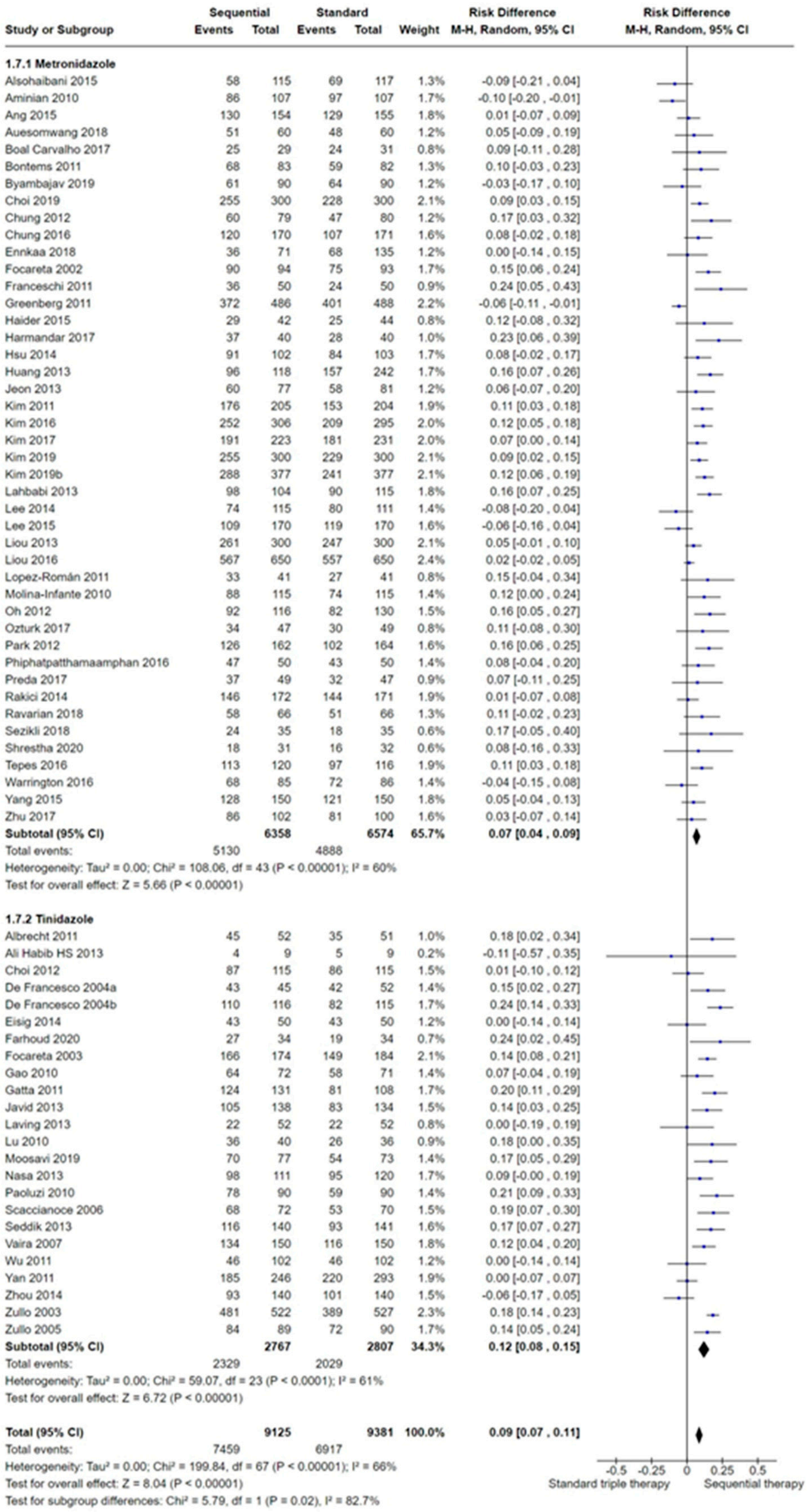

Study or Subgroup	Sequential Events	Sequential Total	Standard Events	Standard Total	Weight	Risk Difference M-H, Random, 95% CI
**1.7.1 Metronidazole**						
Alsohaibani 2015	58	115	69	117	1.3%	-0.09 [-0.21 , 0.04]
Aminian 2010	86	107	97	107	1.7%	-0.10 [-0.20 , -0.01]
Ang 2015	130	154	129	155	1.8%	0.01 [-0.07 , 0.09]
Auesomwang 2018	51	60	48	60	1.2%	0.05 [-0.09 , 0.19]
Boal Carvalho 2017	25	29	24	31	0.8%	0.09 [-0.11 , 0.28]
Bontems 2011	68	83	59	82	1.3%	0.10 [-0.03 , 0.23]
Byambajav 2019	61	90	64	90	1.2%	-0.03 [-0.17 , 0.10]
Choi 2019	255	300	228	300	2.1%	0.09 [0.03 , 0.15]
Chung 2012	60	79	47	80	1.2%	0.17 [0.03 , 0.32]
Chung 2016	120	170	107	171	1.6%	0.08 [-0.02 , 0.18]
Ennkaa 2018	36	71	68	135	1.2%	0.00 [-0.14 , 0.15]
Focareta 2002	90	94	75	93	1.7%	0.15 [0.06 , 0.24]
Franceschi 2011	36	50	24	50	0.9%	0.24 [0.05 , 0.43]
Greenberg 2011	372	486	401	488	2.2%	-0.06 [-0.11 , -0.01]
Haider 2015	29	42	25	44	0.8%	0.12 [-0.08 , 0.32]
Harmandar 2017	37	40	28	40	1.0%	0.23 [0.06 , 0.39]
Hsu 2014	91	102	84	103	1.7%	0.08 [-0.02 , 0.17]
Huang 2013	96	118	157	242	1.7%	0.16 [0.07 , 0.26]
Jeon 2013	60	77	58	81	1.2%	0.06 [-0.07 , 0.20]
Kim 2011	176	205	153	204	1.9%	0.11 [0.03 , 0.18]
Kim 2016	252	306	209	295	2.0%	0.12 [0.05 , 0.18]
Kim 2017	191	223	181	231	2.0%	0.07 [0.00 , 0.14]
Kim 2019	255	300	229	300	2.1%	0.09 [0.02 , 0.15]
Kim 2019b	288	377	241	377	2.1%	0.12 [0.06 , 0.19]
Lahbabi 2013	98	104	90	115	1.8%	0.16 [0.07 , 0.25]
Lee 2014	74	115	80	111	1.4%	-0.08 [-0.20 , 0.04]
Lee 2015	109	170	119	170	1.6%	-0.06 [-0.16 , 0.04]
Liou 2013	261	300	247	300	2.1%	0.05 [-0.01 , 0.10]
Liou 2016	567	650	557	650	2.4%	0.02 [-0.02 , 0.05]
Lopez-Román 2011	33	41	27	41	0.8%	0.15 [-0.04 , 0.34]
Molina-Infante 2010	88	115	74	115	1.4%	0.12 [0.00 , 0.24]
Oh 2012	92	116	82	130	1.5%	0.16 [0.05 , 0.27]
Ozturk 2017	34	47	30	49	0.8%	0.11 [-0.08 , 0.30]
Park 2012	126	162	102	164	1.6%	0.16 [0.06 , 0.25]
Phiphatpatthamaamphan 2016	47	50	43	50	1.4%	0.08 [-0.04 , 0.20]
Preda 2017	37	49	32	47	0.9%	0.07 [-0.11 , 0.25]
Rakici 2014	146	172	144	171	1.9%	0.01 [-0.07 , 0.08]
Ravarian 2018	58	66	51	66	1.3%	0.11 [-0.02 , 0.23]
Sezikli 2018	24	35	18	35	0.6%	0.17 [-0.05 , 0.40]
Shrestha 2020	18	31	16	32	0.6%	0.08 [-0.16 , 0.33]
Tepes 2016	113	120	97	116	1.9%	0.11 [0.03 , 0.18]
Warrington 2016	68	85	72	86	1.4%	-0.04 [-0.15 , 0.08]
Yang 2015	128	150	121	150	1.8%	0.05 [-0.04 , 0.13]
Zhu 2017	86	102	81	100	1.6%	0.03 [-0.07 , 0.14]
**Subtotal (95% CI)**		**6358**		**6574**	**65.7%**	**0.07 [0.04 , 0.09]**
Total events:	5130		4888			
Heterogeneity: Tau^2 = 0.00; Chi^2 = 108.06, df = 43 (P < 0.00001); I^2 = 60%						
Test for overall effect: Z = 5.66 (P < 0.00001)						
**1.7.2 Tinidazole**						
Albrecht 2011	45	52	35	51	1.0%	0.18 [0.02 , 0.34]
Ali Habib HS 2013	4	9	5	9	0.2%	-0.11 [-0.57 , 0.35]
Choi 2012	87	115	86	115	1.5%	0.01 [-0.10 , 0.12]
De Francesco 2004a	43	45	42	52	1.4%	0.15 [0.02 , 0.27]
De Francesco 2004b	110	116	82	115	1.7%	0.24 [0.14 , 0.33]
Eisig 2014	43	50	43	50	1.2%	0.00 [-0.14 , 0.14]
Farhoud 2020	27	34	19	34	0.7%	0.24 [0.02 , 0.45]
Focareta 2003	166	174	149	184	2.1%	0.14 [0.08 , 0.21]
Gao 2010	64	72	58	71	1.4%	0.07 [-0.04 , 0.19]
Gatta 2011	124	131	81	108	1.7%	0.20 [0.11 , 0.29]
Javid 2013	105	138	83	134	1.5%	0.14 [0.03 , 0.25]
Laving 2013	22	52	22	52	0.8%	0.00 [-0.19 , 0.19]
Lu 2010	36	40	26	36	0.9%	0.18 [0.00 , 0.35]
Moosavi 2019	70	77	54	73	1.4%	0.17 [0.05 , 0.29]
Nasa 2013	98	111	95	120	1.7%	0.09 [-0.00 , 0.19]
Paoluzi 2010	78	90	59	90	1.4%	0.21 [0.09 , 0.33]
Scaccianoce 2006	68	72	53	70	1.5%	0.19 [0.07 , 0.30]
Seddik 2013	116	140	93	141	1.6%	0.17 [0.07 , 0.27]
Vaira 2007	134	150	116	150	1.8%	0.12 [0.04 , 0.20]
Wu 2011	46	102	46	102	1.2%	0.00 [-0.14 , 0.14]
Yan 2011	185	246	220	293	1.9%	0.00 [-0.07 , 0.07]
Zhou 2014	93	140	101	140	1.5%	-0.06 [-0.17 , 0.05]
Zullo 2003	481	522	389	527	2.3%	0.18 [0.14 , 0.23]
Zullo 2005	84	89	72	90	1.7%	0.14 [0.05 , 0.24]
**Subtotal (95% CI)**		**2767**		**2807**	**34.3%**	**0.12 [0.08 , 0.15]**
Total events:	2329		2029			
Heterogeneity: Tau^2 = 0.00; Chi^2 = 59.07, df = 23 (P < 0.0001); I^2 = 61%						
Test for overall effect: Z = 6.72 (P < 0.00001)						
**Total (95% CI)**		**9125**		**9381**	**100.0%**	**0.09 [0.07 , 0.11]**
Total events:	7459		6917			
Heterogeneity: Tau^2 = 0.00; Chi^2 = 199.84, df = 67 (P < 0.00001); I^2 = 66%						
Test for overall effect: Z = 8.04 (P < 0.00001)						
Test for subgroup differences: Chi^2 = 5.79, df = 1 (P = 0.02), I^2 = 82.7%						

Figure S3. Forest plot comparison: sequential therapy versus standard triple therapy. Nitroimidazole type. M-H: Mantel–Haenszel; CI: confidence interval.

Study or Subgroup	Sequential Events	Sequential Total	Standard Events	Standard Total	Weight	Risk Difference M-H, Random, 95% CI	Risk Difference M-H, Random, 95% CI
**1.8.1 Low acid inhibition**							
Franceschi 2011	36	50	24	50	1.0%	0.24 [0.05 , 0.43]	
Moosavi 2019	70	77	54	73	1.6%	0.17 [0.05 , 0.29]	
**Subtotal (95% CI)**		**127**		**123**	**2.6%**	**0.19 [0.09 , 0.29]**	
Total events:	106		78				
Heterogeneity: Tau² = 0.00; Chi² = 0.41, df = 1 (P = 0.52); I² = 0%							
Test for overall effect: Z = 3.70 (P = 0.0002)							
**1.8.2 Standard acid inhibition**							
Albrecht 2011	45	52	35	51	1.2%	0.18 [0.02 , 0.34]	
Ali Habib HS 2013	4	9	5	9	0.2%	-0.11 [-0.57 , 0.35]	
Alsohaibani 2015	58	115	69	117	1.5%	-0.09 [-0.21 , 0.04]	
Aminian 2010	86	107	97	107	1.9%	-0.10 [-0.20 , -0.01]	
Auesomwang 2018	51	60	48	60	1.4%	0.05 [-0.09 , 0.19]	
Boal Carvalho 2017	25	29	24	31	1.0%	0.09 [-0.11 , 0.28]	
Bontems 2011	68	83	59	82	1.5%	0.10 [-0.03 , 0.23]	
Byambajav 2019	61	90	64	90	1.5%	-0.03 [-0.17 , 0.10]	
Choi 2012	87	115	86	115	1.7%	0.01 [-0.10 , 0.12]	
Chung 2012	60	79	47	80	1.4%	0.17 [0.03 , 0.32]	
Chung 2016	120	170	107	171	1.8%	0.08 [-0.02 , 0.18]	
De Francesco 2004a	43	45	42	52	1.6%	0.15 [0.02 , 0.27]	
De Francesco 2004b	110	116	82	115	1.9%	0.24 [0.14 , 0.33]	
Eisig 2014	43	50	43	50	1.4%	0.00 [-0.14 , 0.14]	
Ennkaa 2018	36	71	68	135	1.4%	0.00 [-0.14 , 0.15]	
Farhoud 2020	27	34	19	34	0.8%	0.24 [0.02 , 0.45]	
Focareta 2002	90	94	75	93	2.0%	0.15 [0.06 , 0.24]	
Focareta 2003	166	174	149	184	2.3%	0.14 [0.08 , 0.21]	
Gao 2010	64	72	58	71	1.7%	0.07 [-0.04 , 0.19]	
Greenberg 2011	372	486	401	488	2.4%	-0.06 [-0.11 , -0.01]	
Haider 2015	29	42	25	44	0.9%	0.12 [-0.08 , 0.32]	
Harmandar 2017	37	40	28	40	1.2%	0.23 [0.06 , 0.39]	
Hsu 2014	91	102	84	103	1.9%	0.08 [-0.02 , 0.17]	
Javid 2013	105	138	83	134	1.7%	0.14 [0.03 , 0.25]	
Jeon 2013	60	77	58	81	1.5%	0.06 [-0.07 , 0.20]	
Kim 2011	176	205	153	204	2.1%	0.11 [0.03 , 0.18]	
Kim 2016	252	306	209	295	2.2%	0.12 [0.05 , 0.18]	
Kim 2019b	288	377	241	377	2.3%	0.12 [0.06 , 0.19]	
Lahbabi 2013	98	104	90	115	2.0%	0.16 [0.07 , 0.25]	
Laving 2013	22	52	22	52	1.0%	0.00 [-0.19 , 0.19]	
Liou 2013	261	300	247	300	2.3%	0.05 [-0.01 , 0.10]	
Liou 2016	567	650	557	650	2.5%	0.02 [-0.02 , 0.05]	
Lopez-Román 2011	27	41	33	41	1.0%	-0.15 [-0.34 , 0.04]	
Molina-Infante 2010	88	115	74	115	1.6%	0.12 [0.00 , 0.24]	
Nasa 2013	98	111	95	120	1.9%	0.09 [-0.00 , 0.19]	
Oh 2012	92	116	82	130	1.7%	0.16 [0.05 , 0.27]	
Ozturk 2017	34	47	30	49	1.0%	0.11 [-0.08 , 0.30]	
Paoluzi 2010	78	90	59	90	1.6%	0.21 [0.09 , 0.33]	
Park 2012	126	162	102	164	1.9%	0.16 [0.06 , 0.25]	
Phiphatpatthamaamphan 2016	47	50	43	50	1.6%	0.08 [-0.04 , 0.20]	
Rakici 2014	146	172	144	171	2.1%	0.01 [-0.07 , 0.08]	
Ravarian 2018	58	66	51	66	1.5%	0.11 [-0.02 , 0.23]	
Scaccianoce 2006	68	72	53	70	1.7%	0.19 [0.07 , 0.30]	
Seddik 2013	116	140	93	141	1.8%	0.17 [0.07 , 0.27]	
Sezikli 2018	24	35	18	35	0.8%	0.17 [-0.05 , 0.40]	
Shrestha 2020	18	31	16	32	0.7%	0.08 [-0.16 , 0.33]	
Tepes 2016	113	120	97	116	2.1%	0.11 [0.03 , 0.18]	
Vaira 2007	134	150	116	150	2.0%	0.12 [0.04 , 0.20]	
Warrington 2016	68	85	72	86	1.7%	-0.04 [-0.15 , 0.08]	
Wu 2011	46	102	46	102	1.4%	0.00 [-0.14 , 0.14]	
Yan 2011	185	246	220	293	2.2%	0.00 [-0.07 , 0.07]	
Yang 2015	128	150	121	150	2.0%	0.05 [-0.04 , 0.13]	
Zhou 2014	93	140	101	140	1.7%	-0.06 [-0.17 , 0.05]	
Zullo 2003	481	522	389	527	2.5%	0.18 [0.14 , 0.23]	
Zullo 2005	84	89	72	90	1.9%	0.14 [0.05 , 0.24]	
**Subtotal (95% CI)**		**7296**		**7458**	**90.9%**	**0.08 [0.06 , 0.11]**	
Total events:	5954		5502				
Heterogeneity: Tau² = 0.00; Chi² = 171.39, df = 54 (P < 0.00001); I² = 68%							
Test for overall effect: Z = 6.77 (P < 0.00001)							
**1.8.3 High acid inhibition**							
Gatta 2011	124	131	81	108	2.0%	0.20 [0.11 , 0.29]	
Lee 2014	74	115	80	111	1.6%	-0.08 [-0.20 , 0.04]	
Lee 2015	109	170	119	170	1.8%	-0.06 [-0.16 , 0.04]	
Preda 2017	37	49	32	47	1.1%	0.07 [-0.11 , 0.25]	
**Subtotal (95% CI)**		**465**		**436**	**6.5%**	**0.03 [-0.12 , 0.18]**	
Total events:	344		312				
Heterogeneity: Tau² = 0.02; Chi² = 20.06, df = 3 (P = 0.0002); I² = 85%							
Test for overall effect: Z = 0.44 (P = 0.66)							
**Total (95% CI)**		**7888**		**8017**	**100.0%**	**0.08 [0.06 , 0.11]**	
Total events:	6404		5892				
Heterogeneity: Tau² = 0.01; Chi² = 197.03, df = 60 (P < 0.00001); I² = 70%							
Test for overall effect: Z = 6.89 (P < 0.00001)							
Test for subgroup differences: Chi² = 4.57, df = 2 (P = 0.10), I² = 56.2%							

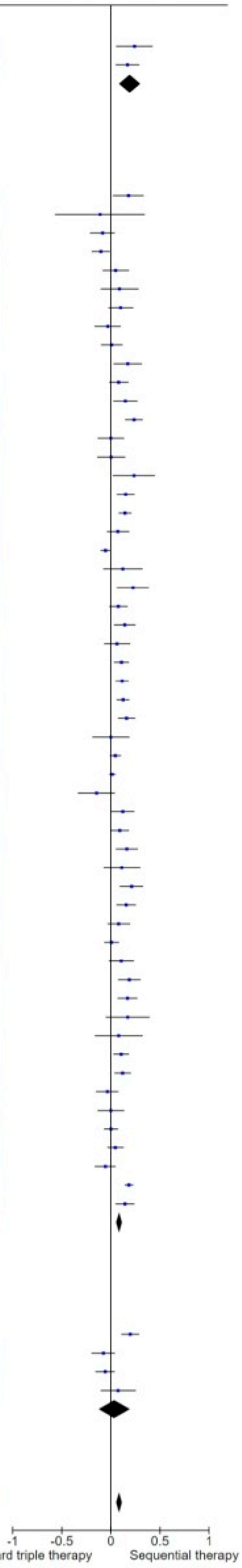

Figure S4. Forest plot comparison: sequential therapy versus standard triple therapy. Proton pump inhibitors for acid inhibition. M-H: Mantel–Haenszel; CI: confidence interval. Dosing for proton pump inhibitors (PPIs): low-dose PPI ranging between 4.5 and 27 mg of omeprazole equivalents, two times per day; standard-dose PPI ranging between 32 and 40 mg of omeprazole equivalents, two times per day; high-dose PPI ranging between 54 and 128 mg of omeprazole equivalents, two times per day.

Study or Subgroup	Sequential Events	Sequential Total	Standard Events	Standard Total	Weight	Risk Difference M-H, Random, 95% CI
**1.9.1 Clarithromycin resistance**						
Alsohaibani 2015	1	10	1	3	1.3%	-0.23 [-0.80 , 0.33]
Auesomwang 2018	6	6	1	3	1.6%	0.67 [0.16 , 1.18]
Bontems 2011	9	16	8	11	2.5%	-0.16 [-0.52 , 0.19]
Gatta 2011	22	24	12	28	4.0%	0.49 [0.27 , 0.70]
Hsu 2014	2	3	2	4	0.9%	0.17 [-0.56 , 0.89]
Liou 2013	10	17	11	20	2.9%	0.04 [-0.28 , 0.36]
Liou 2016	28	45	16	36	4.0%	0.18 [-0.04 , 0.39]
Phiphatpatthamaamphan 2016	3	3	2	4	1.4%	0.50 [-0.05 , 1.05]
Tepes 2016	6	18	4	12	2.6%	0.00 [-0.34 , 0.34]
Vaira 2007	8	9	6	21	3.2%	0.60 [0.32 , 0.89]
Yang 2015	13	25	5	26	3.6%	0.33 [0.08 , 0.58]
Zhou 2014	8	9	7	16	2.9%	0.45 [0.13 , 0.77]
Zullo 2003	7	9	1	6	2.2%	0.61 [0.21 , 1.01]
**Subtotal (95% CI)**		**194**		**190**	**33.1%**	**0.29 [0.14 , 0.44]**
Total events:	123		76			
Heterogeneity: Tau² = 0.04; Chi² = 30.83, df = 12 (P = 0.002); I² = 61%						
Test for overall effect: Z = 3.73 (P = 0.0002)						
**1.9.2 Nitroimidazole resistance**						
Alsohaibani 2015	8	17	8	12	2.5%	-0.20 [-0.55 , 0.16]
Bontems 2011	14	16	12	15	3.5%	0.07 [-0.18 , 0.33]
Hsu 2014	10	11	7	8	3.2%	0.03 [-0.25 , 0.32]
Liou 2013	32	44	41	46	4.7%	-0.16 [-0.32 , -0.00]
Liou 2016	83	101	94	115	5.4%	0.00 [-0.10 , 0.11]
Phiphatpatthamaamphan 2016	9	9	8	9	3.5%	0.11 [-0.15 , 0.37]
Tepes 2016	28	32	16	21	4.0%	0.11 [-0.10 , 0.33]
Vaira 2007	34	35	20	22	5.1%	0.06 [-0.07 , 0.19]
Yang 2015	40	55	38	50	4.6%	-0.03 [-0.20 , 0.13]
Zhou 2014	41	47	39	43	5.1%	-0.03 [-0.16 , 0.09]
Zullo 2003	34	36	26	37	4.7%	0.24 [0.08 , 0.41]
**Subtotal (95% CI)**		**403**		**378**	**46.4%**	**0.02 [-0.05 , 0.09]**
Total events:	333		309			
Heterogeneity: Tau² = 0.00; Chi² = 16.55, df = 10 (P = 0.08); I² = 40%						
Test for overall effect: Z = 0.62 (P = 0.53)						
**1.9.3 Dual resistance**						
Bontems 2011	43	46	37	46	5.0%	0.13 [-0.00 , 0.27]
Hsu 2014	1	2	2	3	0.6%	-0.17 [-1.04 , 0.71]
Liou 2013	3	7	2	4	1.2%	-0.07 [-0.68 , 0.54]
Liou 2016	9	18	7	21	3.0%	0.17 [-0.14 , 0.47]
Vaira 2007	0	4	2	7	2.1%	-0.29 [-0.70 , 0.13]
Yang 2015	4	14	1	11	3.1%	0.19 [-0.10 , 0.49]
Zhou 2014	17	37	18	34	3.8%	-0.07 [-0.30 , 0.16]
Zullo 2003	8	10	2	5	1.6%	0.40 [-0.10 , 0.90]
**Subtotal (95% CI)**		**138**		**131**	**20.5%**	**0.08 [-0.04 , 0.19]**
Total events:	85		71			
Heterogeneity: Tau² = 0.00; Chi² = 8.23, df = 7 (P = 0.31); I² = 15%						
Test for overall effect: Z = 1.32 (P = 0.19)						
**Total (95% CI)**		**735**		**699**	**100.0%**	**0.12 [0.05 , 0.20]**
Total events:	541		456			
Heterogeneity: Tau² = 0.02; Chi² = 84.56, df = 31 (P < 0.00001); I² = 63%						
Test for overall effect: Z = 3.21 (P = 0.001)						
Test for subgroup differences: Chi² = 9.90, df = 2 (P = 0.007), I² = 79.8%						

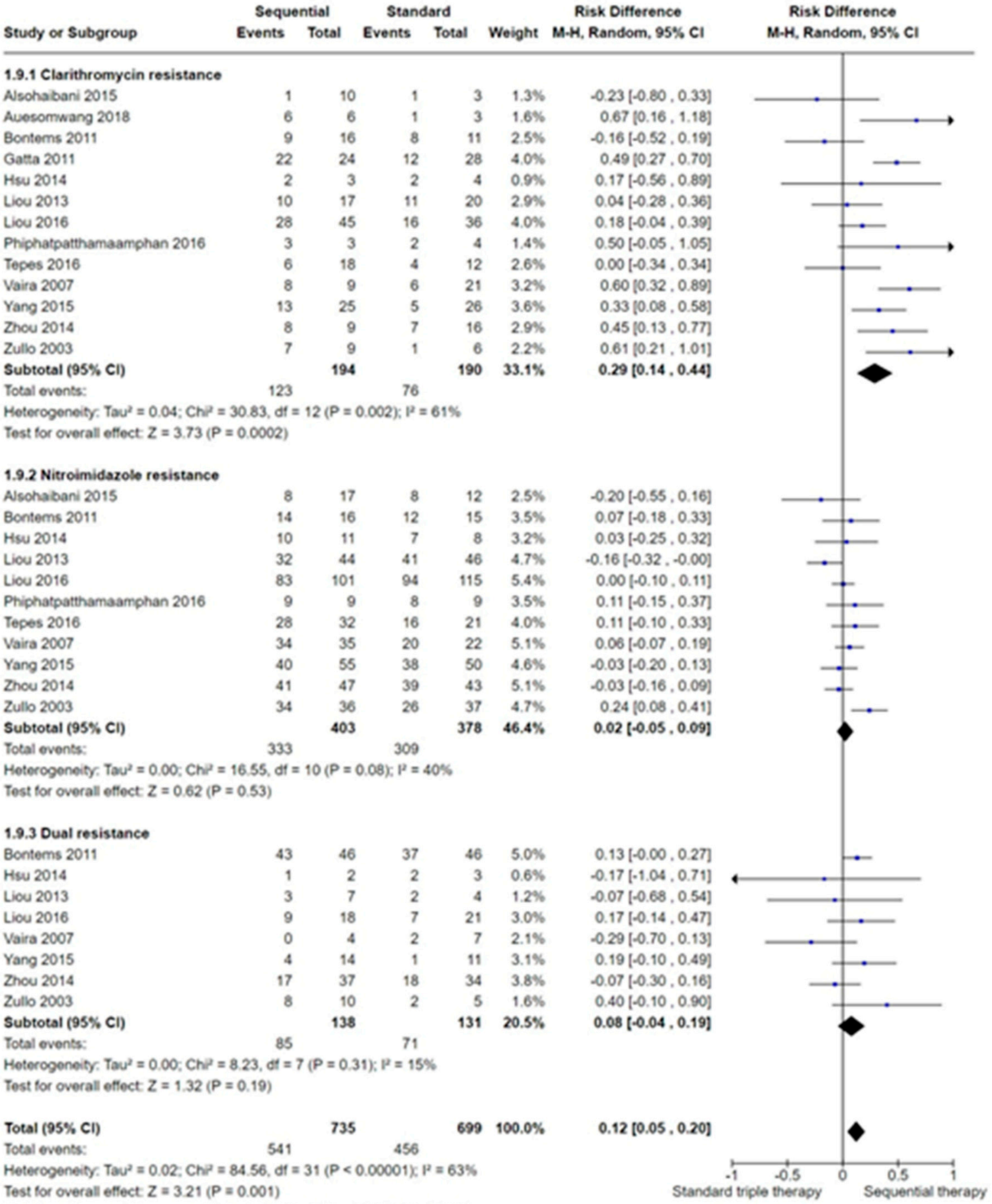

Figure S5. Forest plot comparison: sequential therapy versus standard triple therapy. Bacterial antibiotic resistance. M-H: Mantel–Haenszel; CI: confidence interval.

Study or Subgroup	Sequential Events	Sequential Total	Standard Events	Standard Total	Weight	Risk Difference M-H, Fixed, 95% CI
Moosavi 2019	23	77	43	73	1.2%	-0.29 [-0.44 , -0.14]
Lahbabi 2013	10	104	32	115	1.7%	-0.18 [-0.28 , -0.08]
Sezikli 2018	8	35	14	35	0.6%	-0.17 [-0.39 , 0.04]
Liou 2013	142	300	164	300	4.8%	-0.07 [-0.15 , 0.01]
Zhu 2017	17	102	23	100	1.6%	-0.06 [-0.17 , 0.05]
Yang 2015	40	149	49	148	2.4%	-0.06 [-0.17 , 0.04]
Liou 2016	245	642	271	642	10.3%	-0.04 [-0.09 , 0.01]
Chung 2012	21	80	23	79	1.3%	-0.03 [-0.17 , 0.11]
Chung 2016	55	170	60	171	2.7%	-0.03 [-0.13 , 0.07]
Lee 2014	34	115	35	111	1.8%	-0.02 [-0.14 , 0.10]
Zullo 2003	36	522	45	527	8.4%	-0.02 [-0.05 , 0.02]
Greenberg 2011	33	486	41	488	7.8%	-0.02 [-0.05 , 0.02]
Zullo 2005	9	89	10	90	1.4%	-0.01 [-0.10 , 0.08]
Javid 2013	22	124	23	123	2.0%	-0.01 [-0.11 , 0.09]
Preda 2017	6	49	6	47	0.8%	-0.01 [-0.14 , 0.13]
Scaccianoce 2006	8	72	16	141	1.5%	-0.00 [-0.09 , 0.09]
Aminian 2010	2	107	2	107	1.7%	0.00 [-0.04 , 0.04]
Molina-Infante 2010	29	115	29	115	1.8%	0.00 [-0.11 , 0.11]
Vaira 2007	25	150	25	150	2.4%	0.00 [-0.08 , 0.08]
Hsu 2014	9	102	9	103	1.6%	0.00 [-0.08 , 0.08]
Seddik 2013	36	129	36	131	2.1%	0.00 [-0.10 , 0.11]
Lu 2010	7	40	6	36	0.6%	0.01 [-0.16 , 0.18]
Warrington 2016	36	88	35	88	1.4%	0.01 [-0.13 , 0.16]
Boal Carvalho 2017	6	29	6	31	0.5%	0.01 [-0.19 , 0.22]
Albrecht 2011	10	52	9	51	0.8%	0.02 [-0.13 , 0.17]
Kim 2016	132	295	122	284	4.6%	0.02 [-0.06 , 0.10]
Choi 2012	15	115	37	345	2.8%	0.02 [-0.05 , 0.09]
Franceschi 2011	12	50	21	100	1.1%	0.03 [-0.11 , 0.17]
Park 2012	40	162	35	164	2.6%	0.03 [-0.06 , 0.12]
Oh 2012	32	116	31	130	2.0%	0.04 [-0.07 , 0.15]
Gao 2010	14	72	11	71	1.1%	0.04 [-0.08 , 0.16]
Kim 2019b	65	193	49	166	2.9%	0.04 [-0.05 , 0.14]
Ennkaa 2018	31	72	52	134	1.5%	0.04 [-0.10 , 0.18]
Tepes 2016	24	117	17	110	1.8%	0.05 [-0.05 , 0.15]
Kim 2011	36	205	24	204	3.3%	0.06 [-0.01 , 0.13]
Ozturk 2017	22	47	20	49	0.8%	0.06 [-0.14 , 0.26]
Ravarian 2018	13	66	9	66	1.1%	0.06 [-0.07 , 0.19]
Bontems 2011	37	83	31	82	1.3%	0.07 [-0.08 , 0.22]
Nasa 2013	26	111	17	120	1.8%	0.09 [-0.01 , 0.19]
Byambajav 2019	20	90	11	88	1.4%	0.10 [-0.01 , 0.21]
Paoluzi 2010	34	90	25	90	1.4%	0.10 [-0.04 , 0.24]
Lee 2015	86	152	70	160	2.5%	0.13 [0.02 , 0.24]
Auesomwang 2018	46	60	38	60	1.0%	0.13 [-0.03 , 0.30]
Alsohaibani 2015	35	115	17	117	1.9%	0.16 [0.05 , 0.26]
**Total (95% CI)**		**6139**		**6542**	**100.0%**	**0.00 [-0.01 , 0.02]**
Total events:	1589		1649			

Heterogeneity: Chi² = 73.88, df = 43 (P = 0.002); I² = 42%
Test for overall effect: Z = 0.09 (P = 0.93)
Test for subgroup differences: Not applicable

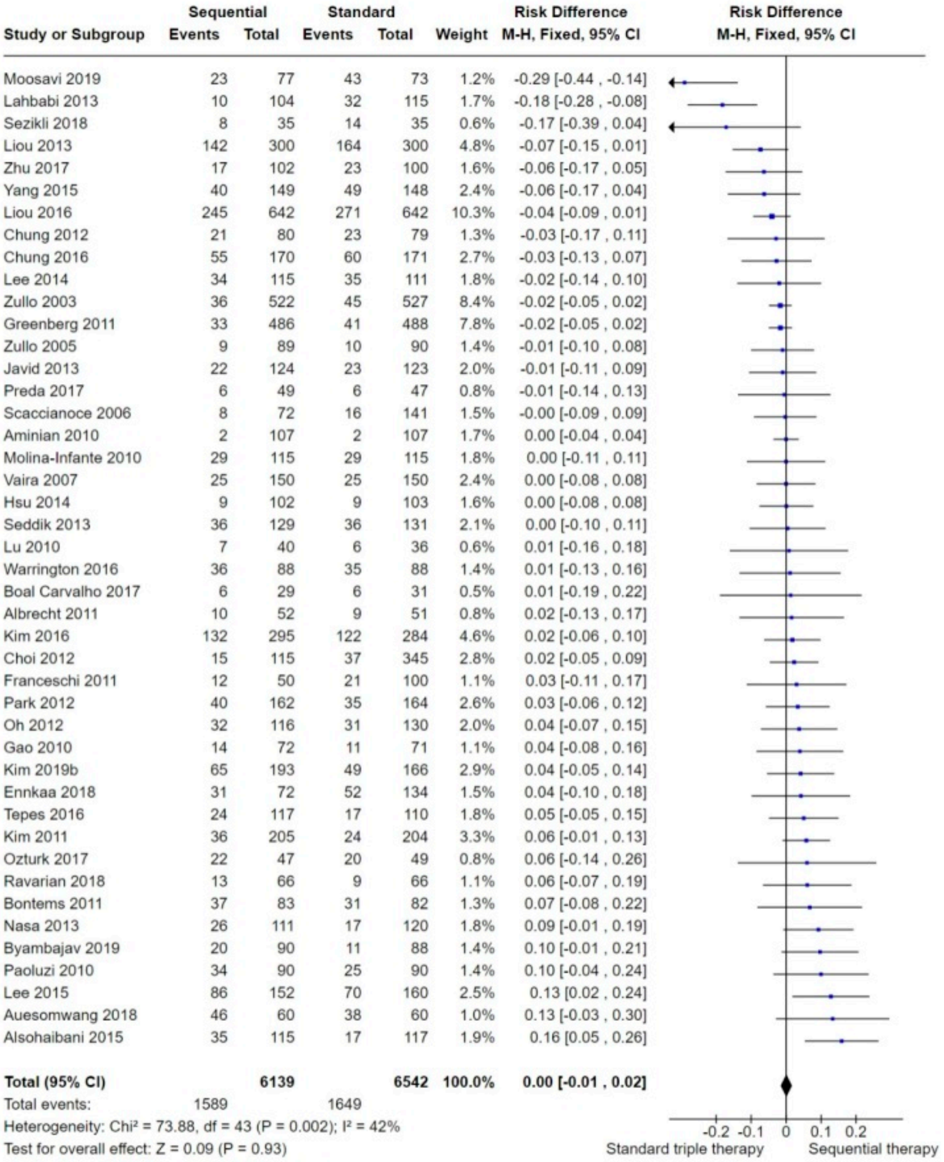

Figure S6. Forest plot comparison: sequential therapy versus standard triple therapy. Adverse events rate**.** M-H: Mantel–Haenszel; CI: confidence interval.

Table S1. Characteristics of included studies.

First author	Year	Country	Age (children/ adults)	*H. pylori* diagnostic method	STT regimen (name; dose and timing of antibiotic administratio n)	Lengt h of STT	ITT eradicat ion rate n/N (%) (95% CI) for STT regime n	PP eradicat ion rate n/N (%) (95% CI) for STT regime n	Incidence of AEs n/N (%) for STT regimen	Complia nce for STT regimen (%)	SEQ regimen (name; dose and timing of antibiotic administratio n)	ITT eradicat ion rate n/N (%) (95% CI) for SEQ regime n	PP eradicat ion rate n/N (%) (95% CI) for SEQ regime n	Inciden ce of AEs n/N (%) for SEQ regime n	Complia nce for STT regimen (%)	Method and time of assessment of *H. pylori* after treatment	PPI dose (standard, low or high)	Nitroimid azole type
Albrecht [88]	2011	Poland	Children	UBT, histopathology and RUT	O 20 mg, C 500 mg and A 1000 mg b.i.d + placebo 3d	7 days	35/51 (68.6) (54 to 80)	NA	9/51 (17.6)	> 95	O 20 mg and A 1000 mg b.i.d for 5 days + O 20 mg, C 500 mg, and T 500 mg b.i.d for 5 days	45/52 (86.5) (74 to 94)	NA	10/52 (19.2)	> 95	UBT 6-8 weeks	standard	T
Ali Habib HS [86]	2013	Saudi Arabia	Children	Histopathology	R 20 mg, C 250 mg and A 500 mg b.i.d	10 days	5/9 (55.6)	5/9 (55.6)	NA	> 95	R 20 mg and A 500 mg b.i.d for 5 days + R 20 mg, C 250 mg and T 500 mg b.i.d for 5 days	4/9 (44.4)	4/7 (57.1)	NA	77	UBT 6 weeks	standard	T
Alsohaibani [85]	2015	Saudi Arabia	Adults	UBT, histopathology and RUT	E 20 mg, C 500 mg and A 1000 mg b.i.d	14 days	58/115 (50.4)	58/93 (62.4)	35/115 (30.4)	NA	E 20 mg, and A 1000mg b.i.d for 5 days + O 20 mg, C 500 mg and M 500 mg b.i.d for 5 days	69/117 (59)	69/102 (67.6)	17/117 (14.5)	NA	UBT 6 weeks	standard	M
Aminian [71]	2010	Iran	Adults	Histopathology	O 20 mg, C 500 mg and A 1000 mg b.i.d	10 days	97/107 (90.7)	97/107 (90.7)	2/107 (1.9)	100	O 20 mg and A 1000mg b.i.d for 5 days + O 20 mg, C 500 mg	86/107 (80.4)	86/106 (81.1)	2/107 (1.9)	100	SAT 8 weeks	standard	M

											and M 500 mg b.i.d for 5 days							
Ang [94]	2015	Singapore	Adults	UBT, histopathology and RUT	PPI, C 500 mg and A 1000 mg b.i.d	10 days	129/155 (83.2) (76.6 to 88.3)	129/139 (92.8) (87.3 to 96.1)	NA	99.3	PPI and A 1000mg b.i.d for 5 days + PPI, C 500 mg and M 500 mg b.i.d for 5 days	130/154 (84.4) (77.9 to 89.3)	128/136 (94.1) (88.8 to 97.0)	NA	94.4	UBT 4 weeks	standard	M
Auesomwang [82]	2018	Thailand	Adults	RUT	L 60 mg, C 500 mg and A 1000 mg b.i.d	10 days	48/60 (80)	48/59 (81.4)	38/60 (63.3)	98.3	L 60 mg and A 1000 mg b.i.d for 5 days + L 30 mg, C 500 mg and M 400 mg b.i.d for 5 days	51/60 (85)	51/54 (94.4)	46/60 (79.3)	93.1	UBT 4 weeks	standard	M
Boal Carvalho [98]	2017	Portugal	Adults	Histopathology	P 40 mg, C 500 mg and A 1000 mg b.i.d	14 days	24/31 (77.4)	23/27 (85.2)	30/31 (96.8)	96.8	P 40 mg and A 1000 mg b.i.d for 5 days + O 20 mg, C 500 mg and M 500 mg b.i.d for 5 days	25/29 (86.2)	23/27 (85.2)	6/29 (20.7)	93.1	UBT 4-6 weeks	standard	M
Bontems [89]	2011	Belgium, France and Italy	Children	Histopathology	O 20 mg, C 500 mg and A 1000 mg b.i.d	7 days	59/82 (71.9)	59/73 (80.8)	NA	NA	O 20 mg and A 100 0mg b.i.d for 5 days + O 20 mg, C 500 mg and M 500 mg b.i.d for 5 days	68/83 (81.9)	68/77 (88.3)	NA	NA	UBT 8 weeks	standard	M
Byambajav [95]	2019	Mongolia	Adults	SAT, histopathology and RUT	P 40 mg, C 500 mg and A 1000 mg b.i.d	10 days	64/90 (71.1)	64/88 (72.7)	11/88 (12.5)	99	P 40 mg and A 1000 mg b.i.d for 5 days + P 40 mg, C 500 mg	61/90 (67.8)	61/89 (97.6)	20/90 (22.2)	98	SAT 4 weeks	standard	M

											and M 500 mg b.i.d for 5 days							
Choi [47]	2012	Korea	Adults	NA	R 20 mg, C 500 mg and A 1000 mg b.i.d	7 days; 10 days; 14 days	7-day STT: 81/115 (70.4) 10-day STT: 86/115 (74.7) 14-day STT: 92/115 (80)	7-day STT: 81/107 (75.7) 10-day STT: 86/115 (81.9) 14-day STT: 92/109 (84.4)	7-day STT: 11/115 10-day STT: 14/115 14-day STT: 12/115	> 95	R 20 mg and A 1000mg b.i.d for 5 days + R 20 mg, C 250 mg and T 500 mg b.i.d for 5 days	87/115 (75.6)	87/106 (82)	15/115	> 95	UBT 4 weeks	standard	T
Choi [101]	2019	NA	NA	Histopathology	PPI, C and A b.i.d	7 days	228/300 (76.2)	NA	NA	NA	R and A b.i.d for 5 days + PPI, C and M for 5 days	255/300 (85.1)	NA	NA	NA	UBT 6 weeks	standard	M
Chung [48]	2012	Korea	Adults	NA	L 30 mg, C 500 mg and A 1000 mg b.i.d	10 days	47/80 (58.7) (47.9 to 69.5)	46/68 (67.6) (56.5 to 78.7)	21/80 (26.3)	96.2	L 30 mg and A 1000 mg b.i.d for 5 days + E 30 mg, C 500 mg and M 500 mg b.i.d for 5 days	60/79 (76) (66.5 to 85.3)	59/68 (86.8) (78.7 to 94.8)	23/79 (29.1)	96.2	UBT 4-6 weeks	standard	M
Chung [54]	2016	Korea	Adults	Histopathology	P 40 mg, C 500 mg and A 1000 mg b.i.d	10 days	107/171 (62.6)	106/128 (82.8)	60/171 (35.1)	96.5	P 40 mg and A 1000mg b.i.d for 5 days + P 40 mg, C 500 mg and M 500 mg b.i.d for 5 days	120/170 (70.6)	119/133 (89.5)	55/170 (32.4)	99.4	UBT 4 weeks	standard	M
De Francesco a [41]	2004	Italy	Adults	NA	R 20 mg, C 500 mg and A 1000 mg	10 days	42/52 (80.7)	42/51 (82.3)	NA	NA	R 20 mg and A 1000 mg b.i.d for 5 days + R 20	43/45 (95.5)	43/44 (97.7)	NA	NA	UBT 6-8 weeks	standard	T

											mg, C 250 mg and T 500 mg b.i.d for 5 days							
De Francesco b [35]	2004	Italy	Adults	NA	R 20 mg, C 500 mg and A 1000 mg	7 days; 10 days,	7-day STT: 82/115 (71.3) 10-day STT: 93/116 (80.1)	7-day STT: 82/114 (71.9) 10-day STT: 93/113 (82.3)	7-day STT: (6) 10-day STT: (7.7)	NA	R 20 mg and A 1000mg b.i.d for 5 days + R 20 mg, C 250 mg and T 500 mg b.i.d for 5 days	110/116 (94.8)	110/115 (65.6)	(10.3)	NA	NA	standard	T
Eisig [102]	2014	Brazil	Adults	Histopathology and RUT	L 30 mg, C 500 mg and A 1000 mg b.i.d	10 days	43/50 (86)	43/49 (87.7)	NA	NA	L 30 mg and A 1000mg b.i.d for 5 days + E 30 mg, C 500 mg and T 500 mg b.i.d for 5 days	43/50 (86)	43/48 (89.6)	NA	NA	UBT 4 weeks	standard	T
Ennkaa [80]	2018	Turkey	Adults	RUT	P 40 mg, C 500 mg and A 1000 mg b.i.d	10 days; 14 days	10-day STT: 28/62 (45.0) (32.6 to 57.4) 14-day STT: 40/73 (54.8) (43.6 to 66.4)	10-day STT: 28/43 (65.1) (50.7 to 79.3) 14-day STT: 40/58 (69.0) (57.1 to 80.9)	10-day STT: 23/62 (37.1)	NA	P 40 mg and A 1000 mg b.i.d for 5 days + P 40 mg, C 500 mg and M 500 mg b.i.d for 5 days	36/71 (50.7) (39.1 to 62.3)	36/51 (70.6) (58.1 to 83.1)	31/71 (43.7)	NA	SAT 4 weeks	standard	T
Farhoud [96]	2020	Egypt	Adults	RUT and UBT	L 30 mg, C 500 mg and A 1000 mg b.i.d	14 days	19/34 (55.9)	19/30 (63.3)	NA	reported as excellent	L 30 mg and A 1000 mg b.i.d for 5 days + L 15 mg, C 500 mg and T 500 mg b.i.d for 5 days	27/34 (79.4)	27/30 (90.0)	NA	reported as excellent	UBT 6 weeks	standard	T

Focareta [37]	2002	Italy	Adults	RUT	O 20 mg, C 500 mg and A 1000 mg b.i.d	7 days	75/93 (80.6)	75/93 (80.6)	NA	NA	O 20 mg and A 1000 mg b.i.d for 5 days + O 20 mg, C 500 mg and M 500 mg b.i.d for 5 days	90/94 (95.7)	90/94 (95.7)	NA	NA	SAT and UBT 6 weeks	standard	M
Focareta [38]	2003	Italy	Adults	Histopathology and UBT	E 20 mg, C 500 mg and A 1000 mg b.i.d	7 days	149/184 (80.9)	NA	NA	NA	E 20 mg and A 1000 mg b.i.d for 5 days + O 20 mg, C 500 mg and T 500 mg b.i.d for 5 days	166/174 (95.4)	NA	NA	NA	SAT and UBT 6 weeks	standard	T
Franceschi [45]	2011	Italy	Adults	UBT	7-day STT: L 15 mg, C 500 mg and A 1000 mg b.i.d High-dose 7-day STT: L 15 mg, C 500 mg and A 1000 mg and t.i.d	7 days	7-day STT: 24/50 (48) High-dose7-day STT: 36/50 (72)	7-day STT: 25/50 (50) High-dose7-day STT: 37/50 (74)	7-day STT: 10/50 (20) High-dose 7-day STT: 11/50 (22)	NA	L 15 mg and A 1000 mg b.i.d for 5 days + L 15 mg, C 500 mg and T 500 mg b.i.d for 5 days	36/50 (72)	37/50 (74)	12/50 (24)	NA	UBT 6 weeks	low	T
Gao [58]	2010	China	Adults	NA	O 20 mg, C 500 mg and A 1000 mg b.i.d	7 days	58/71 (80.56)	45/53 (84.91)	11/71 (15.49)	> 95%	O 20 mg and A 1000 mg b.i.d for 5 days + O 20 mg, C 500 mg and T 500 mg b.i.d for 5 days	64/72 (88.89)	55/61 (90.16)	14/72 (19.44)	> 95%	UBT, histology and RUT 4-6 weeks	standard	T
Gatta [31]	2011	Italy	Adults	RUT, histopathology and UBT	E 40 mg, C 500 mg and A 1000 mg b.i.d	7 days	28/108 (26)	12/28 (44.4) (27.6 to 62.7)	NA	NA	E 40 mg and A 100 0mg b.i.d for 5 days + O 20 mg, C 500 mg and T 500 mg	24/131 (18)	22/24 (90.9) (72.2 to 97.5)	NA	NA	UBT 4 weeks	high	T

											b.i.d for 5 days							
Greenberg [87]	2011	Chile, Colombia, Costa Rica, Mexico, Nicaragua and Honduras	Adults	UBT	L 30 mg, C 500 mg and A 1000 mgb.i.d	14 days	401/488 (82.2) (78.5 to 85.5)	401/475 (84.4)	41/ 475 (9)	87.1	L 30 mg and A 1000 mg b.i.d for 5 days + L 15 mg, C 500 mg and T 500 mg b.i.d for 5 days	372/488 (76.5) (72.5 to 80.2)	372/488 (76.2)	33/470 (7)	81.1	UBT<br>NA	standard	T
Haider [99]	2015	Ireland	Adults	UBT	O 20 mg, C 500 mg and A 1000 mg b.i.d	7 days	25/44 (56.8)	25/41 (61.0)	NA	97.7	O 20 mg and A 1000mg b.i.d for 5 days + O 20 mg, C 500 mg and M 500 mg b.i.d for 5 days	29/42 (69.0)	29/42 (69.0)	NA	100	UBT<br>8 weeks	standard	M
Harmandar [77]	2017	Turkey	Adults	Histopathology	P 40 mg, C 500 mg and A 1000 mg b.i.d	14 days	28/40 (70.0)	NA	NA	NA	P 40 mg and A 1000 mg b.i.d for 5 days + P 40 mg, C 500 mg and M 500 mg b.i.d for 5 days	37/40 (92.5)	NA	NA	NA	UBT<br>4 weeks	standard	M
Hsu [93]	2014	Japan	Adults	RUT, histopathology or culture	P 40 mg, C 500 mg and A 1000 mg b.i.d	7 days	84/103 (81.6) (74.1 to 89.0)	83/101 (82.2) (74.8 to 89.6)	9/103 (8.7) (3.3 to 14.2)	99	P 40 mg and A 1000 mg b.i.d for 5 days + P 40 mg, C 500 mg and M 500 mg b.i.d for 5 days	91/102 (89.2) (83.2 to 95.2)	90/100 (90.0) (84.1 to 95.9)	9/102 (8.8) (3.3 to 14.3)	98	RUT, histopathology, culture and UBT<br>6 weeks	standard	M
Huang [62]	2013	China	Children	RUT, SAT, culture and histology	O 0.8 – 1.0 mg/kg/d + C 20 mg/kg/d + A 30 mg/kg/d	7 days; 10 days	7-day STT: 73/118 (61.9%)	7-day STT: 73/103 (70.8%)	7-day STT: 24/103 (23.3%)	7-day STT: NA	O 0.8 – 1.0 mg/kg/d + A 30 mg/kg/ (for 5 days)	96/118 (81.4%) (74.4 to 84.4)	96/107 (89.7%) (83.9 to 95.5)	32/107 (29.9%)	NA	STA<br>4 weeks	standard	M

							(53.1 to 70.7)<br><br>10-day STT: 84/124 (67.7%) (59.5 to 75.9)	(62.1 to 79.7)<br><br>10-day STT: 84/108 (77.8%) (70.0 to 85.6)	10-day STT: 37/108 (34.3%)	10-day STT: NA	and O 0.8 – 1.0 mg/kg/d + C 20 mg/kg/d + M 20 mg/kg/d (for 5 days)							
Javid [67]	2013	India	Adults	RUT and histology	P 40 mg, C 500 mg and A 1000 mg b.i.d	10 days	83/134 (61.9)	83/123 (67.4)	23/123 (18.7)	>95%	P 40 mg and A 1000 mg b.i.d for 5 days + P 40 mg, C 500 mg and T 500 mg b.i.d for 5 days	105/138 (76)	105/124 (84.6)	22/124 (17.7)	100	Histology and RUT 4 weeks	standard	T
Jeon [146]	2013	Korea	Adults	NA	O 20 mg, C 500 mg and A 1000 mg b.i.d	7 days	58/81 (71.6)	58/76 (76.6)	NA	NA	O 20 mg and A 1000 mg b.i.d for 5 days + P 40 mg, C 500 mg and M 500 mg b.i.d for 5 days	60/77 (77.9)	60/70 (85.7)	NA	NA	NA 8 weeks	standard	M
Kim [46]	2011	Korea	Adults	UBT, RUT and histology	P 40 mg, C 500 mg and A 1000 mg b.i.d	14 days	153/205 (75)	153/180 (85)	24/180 (13.3)	97.2	P 40 mg and A 1000 mg b.i.d for 5 days + P 40 mg, C 500 mg and M 500 mg b.i.d for 5 days	176/205 (85.9)	175/190 (92.6)	36/190 (18.9)	96.8	UBT, RUT and histology 4 weeks	standard	M
Kim [55]	2016	Korea	Adults	RUT and histology	L 30 mg, C 500 mg and A 1000 mg b.i.d	7 days	209/295 (70.8)	206/268 (76.9)	122/295 (43.0)	98.5	L 30 mg and A 100 0mg b.i.d for 5 days + L 30 mg, C 500 mg and M 500	252/306 (82.4)	247/278 (88.8)	132/306 (44.4)	97.2	UBT 4 weeks	standard	M

											mg b.i.d for 5 days							
Kim [104]	2017	NA	Adults	Histology	PPI, C and A b.i.d	7 days	181/231 (78.3)	NA	NA	NA	R and A b.i.d for 5 days + R , C and M b.i.d for 5 days	191/223 (85.7)	NA	NA	NA	UBT 6 weeks	NA	M
Kim [56]	2019	Korea	Adults	Histology	PPI, C and A b.i.d	7 days	229/300 (76.2)	NA	NA	NA	R and A b.i.d for 5 days + R , C and M b.i.d for 5 days	255/300 (85.1)	NA	NA	NA	UBT 6 weeks	NA	M
Kim b [57]	2019	Korea	Adults	UBT, RUT and histology	L 30 mg, C 500 mg, A 1000 mg, b.i.d	7 days	241/377 (63.9)	215/301 (71.4)	215/301 (71.4)	91	L 30 mg and A 1000 mg b.i.d for 5 days + L 30 mg, C 500 mg and M 500 mg b.i.d for 5 days	288/377 (76.4)	244/287 (85)	65/193 (33.4)	88	UBT 4-6 weeks	standard	M
Lahbabi [69]	2013	Morocco	Adults	Histology and/or STA	O 20 mg, C 500 mg and A 1000 mg b.i.d	7 days	90/115 (78.2)	90/113 (79.6)	32/115 (827.8)	92.2	O 20 mg and A 1000 mg b.i.d for 5 days + P 40 mg, C 500 mg and M 500 mg b.i.d for 5 days	98/104 (94.2)	98/102 (96.1)	10/104 (9.6)	96.1	UBT 12 weeks	standard	M
Laving [91]	2013	Kenya	Children	Histology and/or STA	P 40 mg, C 500 mg and A 1000 mg b.i.d	10 days	22/52 (42.3)	22/45 (48.8)	NA	NA	O 1.0 mg/kg/d + A 50 mg/kg/ for 5 days and O 0.8 – 1.0 mg/kg/d + C 20 mg/kg/d + T mg/kg/d for 5 days	22/52 (42.3)	22/26 (84.6)	NA	NA	Histology or SAT 6 weeks	standard	T

Lee [52]	2014	Korea	Adults	UBT and/or RUT	E 40 mg, C 500 mg and A 1000 mg b.i.d	7 days	74/115 (64.3)	74/108 (68.5)	34/115 (29.5)	NA	E 40 mg and A 1000 mg b.i.d for 5 days + P 40 mg, C 500 mg and M 500 mg b.i.d for 5 days	80/111 (72.1)	80/102 (78.4)	35/111 (31.5)	NA	Histology, UBT and/or RUT	high	M
Lee [147]	2015		Adults	Histology	R 40 mg, C 500 mg and A 1000 mg b.i.d	7 days	109/170 (64)	109/143 (76.2)	86/152 (50.6)	NA	R 40 mg and A 1000 mg b.i.d for 5 days + R 40 mg, C 500 mg and M 500 mg b.i.d for 5 days	119/170 (70)	119/141 (84)	70/160 (41.2)	NA	UBT 6 weeks	high	M
Liou [63]	2013	China	Adults	Serology, RUT, histology, culture and UBT (at least 2 positive)	L 30 mg, C 500 mg and A 1000 mg b.i.d	14 days	247/300 (82.3) (78.0 to 86.6)	243/279 (87.1) (83.2 to 91.0)	164/298 (55)	87	L 30 mg and A 1000 mg b.i.d for 5 days + L 30 mg, C 500 mg and M 500 mg b.i.d for 5 days	261/300 (87.0) (83.2 to 90.8)	258/285 (90.5) (87.1 to 93.9)	142/294 (48)/	91	UBT 6 weeks	standard	M
Liou [66]	2014	China	Adults	Serology, RUT, histology, culture and UBT (at least 2 positive)	NA	14 days	367/424 (86.6)	367/407 (90.2)	NA	NA	NA	367/416 (88.2)	367/401 (91.5)	NA	NA	NA	NA	NA
Liou [83]	2016	Taiwan	Adults	Serology, RUT, histology, culture and UBT	L 30 mg, C 500 mg and A 1000 mg b.i.d	14 days	557/650 (85.7)	548/602 (91)	271/642 (42.2)	94	L 30 mg and A 1000 mg b.i.d for 5 days + L 30 mg, C 500 mg and M 500 mg b.i.d for 5 days	567/650 (87.2)	556/607 (91.6)	245/642 (38.2)	96	UBT 6 weeks	standard	M

Lopz-Román [74]	2011	Puerto Rico	Adults	RUT and histology	O 20 mg, C 500 mg and A 1000 mg b.i.d	10 days	33/41 (80)	NA (84.2)	NA	NA	O 20 mg and A 1000 mg b.i.d for 5 days + P 40 mg, C 500 mg and M 500 mg b.i.d for 5 days	27/41 (65.9	NA (71.1)	NA	NA	UBT 8 weeks	standard	M
Lu [59]	2010	China	Children	NA	O 0.8 – 1.0 mg/kg/d + C 20 mg/kg/d + A 30 mg/kg/d	10 days	26/36 (72.2)	26/33 (78.8)	6/36 (17)	NA	O 1.0 mg/kg/d + A 50 mg/kg/ (for 5 days) and O 0.8 – 1.0 mg/kg/d + C 20 mg/kg/d + T 15 mg/kg/d (for 5 days)	36/40 (90)	36/38 (94.7)	7/40 (18)	NA	UBT, blood test and RUT 4 weeks	standard	T
Molina-Infante [36]	2010	Spain	Adults	UBT, RUT and histology	O 20 mg, C 500 mg and A 1000 mg b.i.d	10 days	74/115 (64%; (55 – 73%)	74/113 (66%; (57 – 74%)	29/115 (25)	97	O 20 mg and A 1000 mg b.i.d for 5 days + P 40 mg, C 500 mg and M 500 mg b.i.d for 5 days	88/115 (76%; (69 – 85%)	88/110 (80%; (3 – 88%)	29/110 (25%)	99	UBT and histology 8 weeks	standard	M
Moosavi [73]	2019	Iran	Adults	RUT and histology	P 40 mg, C 500 mg and A 1000 mg b.i.d	14 days	54/73 (74.0))	52/64 (81.3)	Minor AEs: 36/73 (49.3) Major AEs: 7/73 (9.6)	NA	P 40 mg and A 1000 mg b.i.d for 5 days + P 40 mg, C 500 mg and T 500 mg b.i.d for 5 days	70/77 (90.9)	70/73 (95.9)	Minor AEs: 20/77 (26) Major AEs: 3/77 (3.9)	NA	UBT 4 weeks	low	T
Nasa [68]	2013	India	Adults	RUT and histology	P 40 mg, C 500 mg and A 1000 mg b.i.d	14 days	95/120 (79.1) (1.1 – 85.4)	98/120 (81.6) (73.9 – 87.8)	17/120 (14.6	NA	P 40 mg and A 1000 mg b.i.d for 5 days + P 40 mg, C 500 mg	98/111 (88.2) (80.9 – 93.0)	103/111 (92.8) (85.8 – 96.1)	26/111 (23.5	NA	RUT 4 weeks	standard	T

											and T 500 mg b.i.d for 5 days							
Oh [51]	2012	Korea	Adults	SAT and histology	R 20 mg, C 500 mg and A 1000 mg b.i.d	7 days	82/130 (63.0)	92/116 (79.3)	32/116 (27.5)	NA	R 20 mg and A 1000 mg b.i.d for 5 days + R 20 mg, C 500 mg and M 500 mg b.i.d for 5 days	82/127 (64.5)	91/111 (81.9)	31/130 (23.8)	NA	UBT 4 weeks	standard	M
Ozturk [78]	2012	Turkey	Adults	RUT and histology	O 20 mg, C 500 mg and A 1000 mg b.i.d	10 days	30/49 (61.2)	30/40 (75)	20/49 (40.8)	NA	O 20 mg and A 1000 mg b.i.d for 5 days + O 20 mg, C 500 mg and M 500 mg b.i.d for 5 days	34/47 (72.3)	30/40 (73.9)	22/47 (46.8)	NA	UBT 6-8 weeks	standard	M
Paoluzi [44]	2010	Italy	Adults	NA	E 20 mg, C 500 mg, A 1000 mg, b.i.d	7 days	59/90 (66)	59/78 (75)	25/90 (42)	NA	E 20 mg and A 1000 mg b.i.d for 5 days + E 20 mg, C 500 mg and T 500 mg b.i.d for 5 days	78/90 (86)	78/88 (88)	34/90 (54)	NA	Histology, SAT, UBT and/or RUT 8 weeks	standard	T
Park [49]	2012	Korea	Adults	UBT	R 20 mg, C 500 mg and A 1000 mg b.i.d	7 days	62.2 (54.8 to 69.6)	76.0 (68.5 to 83.5)	NA	87.9	R 20 mg and A 1000 mg b.i.d for 5 days + R 20 mg, C 500 mg and M 500 mg b.i.d for 5 days	77.8 (71.4 to 84.2)	87.9 (82.3 to 93.5)	NA	76.0	UBT 4 weeks	standard	M
Phiphatpatthamaamphan [81]	2016	Thailand	Adults	RUT	R 20 mg, C 1000 mg (long acting) and A 500 mg q.i.d	14 days	43/50 (86.0)	47/48 (97.9)	NA	NA	R 20 mg and A 1000 mg b.i.d for 5 days + R 20	47/50 (94.0)	43/49 (87.8)	NA	NA	UBT 4 weeks	standard	M

											mg, C 1000 mg (long acting) and M 500 mg q.i.d for 5 days							
Preda [97]	2017	Romania	Adults	SAT	E 80 mg, C 500 mg and A 1000 mg b.i.d	10 days	32/47 (68.1)	32/34 (94.0)	6/47	94	E 80 mg and A 1000 mg b.i.d for 5 days + E 80 mg, C 500 mg and M 500 mg b.i.d for 5 days	37/49 (75.5)	37/39 (95.0)	6/49	100	SAT 4 weeks	high	T
Rakici [76]	2014	Turkey	Adults	SAT and Histology	L 30 mg, C 500 mg and A 1000 mg b.i.d	14 days	144/171 (84.2)	144/169 (85.2)	NA	NA	L 30 mg and A 1000 mg b.i.d for 5 days + L 30 mg, C 500 mg and M 500 mg b.i.d for 5 days	146/172 (84.9)	146/170 (85.8)	NA	NA	SAT 4-6 weeks	standard	M
Ravarian [72]	2018	Iran	Adults	RUT and histology	O 20 mg, C 500 mg and A 1000 mg b.i.d	10 days	51/66 (78.0)	NA	9/66 (13.6)	NA	O 20 mg and A 1000 mg b.i.d for 5 days + O 20 mg, C 500 mg and M 500 mg b.i.d for 5 days	58/66 (87.8)	NA	13/66 (19.7)	NA	UBT 4-6 weeks	standard	M
Scaccianoce [42]	2006	Italy	Adults	RUT and histology	E 20 mg, C 500 mg and A 1000 mg b.i.d	7 days 10 days	7-day STT: 53/70 (75.7) (66 to 86) 10-day STT: 58/71 (81.7)	7-day STT: 53/68 (77.9) (68 to 88) 10-day STT: 58/69 (84.1)	NA	>95	E 20 mg and A 1000 mg b.i.d for 5 days + E 20 mg, C 500 mg and T 500 mg b.i.d for 5 days	68/72 (94.4) (89 to 100)	68/70 (97.1) (93 to 100)	NA	>95	UBT 4-6 weeks	standard	T

							(73 to 91)	(75 to 93)									
Seddik [70]	2013	Morocco	Adults	Histology	O 20 mg, C 500 mg and A 1000 mg b.i.d	7 days	93/141 (66)	93/131 (71)	36/131 (27.5)	NA	O 20 mg and A 1000 mg b.i.d for 5 days + O 20 mg, C 500 mg and M 500 mg b.i.d for 5 days	116/140 (82.8)	116/129 (90)	36/129 (27.9)	NA	UBT 4-6 weeks	standard	M
Sezikli [79]	2018	Turkey	Adults	RUT and histology	R 20 mg, C 500 mg and A 1000 mg b.i.d	14 days	18/35 (51.4)	18/35 (51.4)	14/35 (40)	NA	R 20 mg and A 1000 mg b.i.d for 5 days + R 20 mg, C 500 mg and M 500 mg b.i.d for 5 days	24/35 (68.6)	24/35 (68.6)	8/35 (22.9)	NA	UBT 4-6 weeks	standard	M
Shrestha [100]	2020	Nepal	Adults	SAT and histology	E 20 mg, C 500 mg and A 1000 mg b.i.d	14 days	18/31 (58)	NA	NA	58	E 20 mg and A 1000 mg b.i.d for 5 days + E 20 mg, C 500 mg and M 400 mg b.i.d for 5 days	16/32 (86.5)	NA	NA	49	SAT 5 weeks	standard	M
Tepes [90]	2016	Slovenia	Adults	UBT, RUT and histology	E 20 mg, C 500 mg and A 1000 mg b.i.d	7 days	97/116 (83.6)	97/110 (88.2)	17/110 (15.5)	NA	E 20 mg and A 1000 mg b.i.d for 5 days + E 20 mg, C 500 mg and M 400 mg b.i.d for 5 days	113/120 (94.2)	113/117 (96.6)	24/117 (20.5)	NA	UBT 4 weeks	standard	M
Vaira [43]	2007	Italy	Adults	NA	P 40 mg, C 500 mg and A 1000 mg b.i.d	10 days	116/150 (77)	116/146 (79)	25/146 (17.1)	>90	P 40 mg and A 1000 mg b.i.d for 5 days + P 40 mg, C 500 mg	134/150 (89)	133/143 (93)	25/143 (17.5)	>90	UBT 4 -8 weeks	standard	T

											and T 500 mg b.i.d for 5 days							
Warrington [75]	2016	Puerto Rico	Adults	RUT and histology	O 20 mg, C 500 mg and A 1000 mg b.i.d	10 days	72/86 (83.7)	70/83 (84.3)	NA	94.3	O 20 mg and A 1000 mg b.i.d for 5 days + O 20 mg, C 500 mg and M 500 mg b.i.d for 5 days	68/85 (80.0)	66/81 (81.5)	NA	94.3	UBT 6 weeks	standard	M
Wu [60]	2011	China	Adults	NA	E 20 mg, C 500 mg and A 1000 mg b.i.d	14 days	46/51 (90.4)	NA	NA	NA	E 20 mg and A 1000 mg b.i.d for 5 days + P 40 mg, C 500 mg and T 500 mg b.i.d for 5 days	46/51 (90.2)	NA	NA	NA	UBT or endoscopy 4 weeks	standard	T
Yan [61]	2011	China	Adults	RUT and histology	E 20 mg, C 500 mg and A 1000 mg b.i.d	10 days	185 / 246 (75.2)	NA	NA	NA	E 20 mg and A 1000 mg b.i.d for 5 days + E 20 mg, C 500 mg and T 500 mg b.i.d for 5 days	220 / 293 (75.1)	NA	NA	NA	UBT or histology 4-12 weeks	standard	T
Yang [84]	2015	Taiwan	Adults	UBT, RUT and histology	R 20 mg, C 500 mg and A 1000 mg b.i.d	7 days	121/150 (80.7)	121/149 (81.2)	49/148 (33.2)	94.9	R 20 mg and A 1000 mg b.i.d for 5 days + R 40 mg, C 500 mg and M 500 mg b.i.d for 5 days	128/150 (85.3)	128/148 (86.5)	40/149 (26.8)	87.8	UBT 4-8 weeks	standard	M
Zhou [64]	2014	China	Adults	RUT	E 20 mg, C 500 mg and A 1000 mg b.i.d	10 days	93/140 (66.4)	93/128 (72.7)	NA	NA	E 20 mg and A 1000 mg b.i.d for 5 days + E 20	101/140 (72.1)	101/132 (76.5)	NA	NA	UBT or histology 4-12 weeks	standard	T

											mg, C 500 mg and T 500 mg b.i.d for 5 days							
Zhu [65]	2017	China	Children	UBT	O, C and A	14 days	81/100 (81.0)	NA	23/100 (23.0)	NA	O and A for 5 days + O, C, A and M for 5 days	86/102 (84.3)	NA	17/102 (16.7)	NA	UBT NA	NA	M
Zullo [39]	2003	Italy	Adults	Histology	R 20 mg, C 500 mg and A 1000 mg b.i.d	7 days	389/527 (74)(70 – 77.6)	389/507 (77)(73–80.4)	45/527 (9)	93	R 20 mg and A 100 0mg b.i.d for 5 days + R 40 mg, C 500 mg and T 500 mg b.i.d for 5 days	481/522 (92) (89.9 to 94.5)	481/506 (95) (93.2 to 97)	36/522 (7)	90	UBT 6 weeks	standard	T
Zullo [40]	2005	Italy	Adults	RUT and histology	R 20 mg, C 500 mg and A 1000 mg b.i.d	7 days	72/90 (80)(72 to 88)	72/87 (82.8) (75 to 91)	10 / 90 (11.5)	>95	R 20 mg and A 1000 mg b.i.d for 5 days + R 40 mg, C 500 mg and T 500 mg b.i.d for 5 days	84/89 (94.4) (90 to 99)	84/87 (96.6) (93 to 100)	9/89 (10.3)	>95	RUT and histology 6 weeks	standard	T

H. pylori: Helicobacter pylori; STT: standard triple therapy; SEQ: non-bismuth quadruple sequential therapy; CI: confidence interval; IIT: intention to treat; PP per protocol; AEs: adverse events rate; PPI proton pump inhibitor.

Dosing for PPI: low-dose PPI ranging between 4.5 and 27 mg of omeprazole equivalents, two times per day; standard-dose PPI ranging between 32 and 40 mg of omeprazole equivalents, two times per day; high-dose PPI ranging between 54 and 128 mg of omeprazole equivalents, two times per day.

UBT: urea breath test; RUT: rapid urease test; SAT: stool antigen test.

O: omeprazole; P: pantoprazole; E: esomeprazole; L: lansoprazole; R: rabeprazole; A: amoxicillin; C: clarithromycin; M: metronidazole; T: tinidazole; b.i.d: two times a day; q.i.d: four times a day.

NA: not available.

Table S2. Summary of findings.

Outcomes	Illustrative comparative risks* (95% CI) — Assumed risk: Standard triple therapy	Corresponding risk: 10-day sequential regimen	Relative effect (95% CI)	No. of Participants (studies)	Quality of the evidence (GRADE)	Comments
**Is 10-day SEQ efficacy superior to STT?**						
**Patient or population:** participants with *Helicobacter pylori* infection<br>**Settings:** participants naïve to eradication treatment<br>**Intervention:** 10-day sequential regimen<br>**Comparison:** standard triple therapy						
**Eradication proportion**	**Study population**		RD 0.08, 95% CI From 0.06 to 0.10	19,661 (69 studies)	⊕⊕⊕⊝ **moderate** [1]	The results were highly heterogeneous (I^2 = 68%), and 37 studies did not demonstrate differences between therapies.
	**748 per 1000**	**824 per 1000** (816 to 832)				
	**Moderate**					
	**748 per 1000**	**824 per 1000** (816 to 832)				
**Geographic region**	**Study population**		RD 0.08, 95% CI From 0.06 to 0.10	18,746 (68 studies)	⊕⊕⊝⊝ **low**[1,2,3]	The results were highly heterogeneous (I^2 = 68%) with significant differences between subgroups.<br>The Latin American subgroup showed no consistent results with the remaining subgroups and there was a tendency toward better efficacy with STT than with SEQ in all four included studies, although three studies did not demonstrate differences between therapies.
	**742 per 1000**	**818 per 1000** (810 to 826)				
	**Moderate**					
	**742 per 1000**	**839 per 1000** (810 to 826)				
**Publication date**	**Study population**		RD 0.08, 95% CI From 0.06 to 0.10	19,813 (69 studies)	⊕⊕⊕⊝ **moderate** 1,2,3	The results were more heterogeneous (I^2 = 60%) in the "after 2010" subgroup.
	**744 per 1000**	**819 per 1000** (811 to 827)				
	**Moderate**					
	**744 per 1000**	**819 per 1000** (811 to 827)				
**STT length**	**7 days**		RD 0.13, 95% CI from 0.11 to 0.15	8,834 (29 studies)	⊕⊕⊕⊕ **high**[4]	Eight out of twenty-nine studies did not demonstrate differences when 7-day STT was compared to 10-day SEQ. The results for this comparison were consistent (I^2 = 41%).
	**Study population**					
	**725 per 1000**	**870 per 1000** (848 to 892)				
	**Moderate**					
	**720 per 1000**	**864 per 1000** (842 to 886)				
	**10 days**		RD 0.06, 95% CI from 0.02 to 0.09	5,236 (27 studies)	⊕⊕⊕⊕ **high**[1,4]	In this subgroup, 10-day SEQ was better than 10-day STT; however, heterogeneity between studies was greater (I^2 =51%) than in the 7- day STT subgroup analysis. Six studies out of twenty-seven demonstrated that 10-day STT was superior to 10-day SEQ. Eighteen studies could not demonstrate differences between therapies.
	**Study population**					
	**720 per 1000**	**768 per 1000** (751 to 784)				
	**Moderate**					
	**720 per 1000**	**750 per 1000** (744 to 823)				
	**14 days**		RD 0.04, 95% CI	6,300 (19 studies)	⊕⊕⊕⊕	14-day STT was marginally not superior to 10- day SEQ.
	**Study population**					

	**789 per 1000**	**811 per 1000** (795 to 825)	from 0.01 to 0.07		**high**[1,4]	
	**Moderate**					
	**779 per 1000**	**819 per 1000** (803 to 835)				
**Bacterial antibiotic resistance**	**Study population**		RD 0.12, 95% CI from 0.05 to 0.20	1,434 (13 studies)	⊕⊖⊖⊖ **very low**[1,2,3,5,6]	SEQ was superior to STT in those patients with primary clarithromycin resistant strains only.
	**652 per 1000**	**736 per 1000** (703 to 768)				
	**Moderate**					
	**550 per 1000**	**660 per 1000** (572 to 748)				
**Adverse events rate**	**Study population**		RD 0.00, 95% CI from -0.01 to 0.02	12,681 (44 studies)	⊕⊕⊕⊕ **high**[2,7]	No differences were reported between treatment arms.
	**258 per 1000**	**258 per 1000** (247 to 269)				
	**Moderate**					
	**187 per 1000**	**191 per 1000** (168 to 206)				

*The basis for the **assumed risk** (e.g. the median control group risk across studies) is provided in footnotes. The **corresponding risk** (and its 95% confidence interval) is based on the assumed risk in the comparison group and the **relative effect** of the intervention (and its 95% CI).
STT: standard triple therapy; **SEQ**: non-bismuth quadruple sequential therapy.
CI: confidence interval; **RD:** risk difference

GRADE: working group grades of evidence:
High quality: further research is very unlikely to change our confidence in the estimate of effect.
Moderate quality: further research is likely to have an important impact on our confidence in the estimate of effect and may change the estimate.
Low quality: further research is very likely to have an important impact on our confidence in the estimate of effect and is likely to change the estimate.
Very low quality: we are very uncertain about the estimate.

1: there is moderate to substantial unexplained heterogeneity.
2: wide confidence intervals in some subgroups.
3: small number of studies in some subgroups.
4: different STT lengths (different total doses) modify RDs.
5: lack of reporting in most of the studies
6: metronidazole resistance is dose-dependent.
7: longer treatments (higher total dose) led to higher rates of AEs.

File S1: Results: risk of bias in included studies.

In the overall comparison 'Eradication proportion of SEQ versus STT', 20 studies [39,43,46,57,62,64,67,68,72,73,75,80,82-84,87,88,90,93,94] were categorized as ‘low risk of bias’ in all domains of the checklist assessing the quality of the methodology (Figure 2).

Nine studies [59,65,77,79,89,97,100,101,104] were categorized as ‘high risk’ in the item relating to randomization only. From those, eight [59,77,79,89,97,100,101,104] were rated also as 'high risk' in the item relating to allocation.

Nineteen studies [44,49,54-56,59,71,77-79,81,86,89,96,97,99-101,104] were likewise rated as having poor allocation concealment and were flagged as 'high risk'.

A lack of comprehensive reporting of outcomes, as well as scarcity of information related to the assessed quality items within the aforementioned studies, made both selection and performance biases a threat to the validity of the review (Figure 2 and Figure 3).

However, regardless of the potential biases, the subgroup analyses confirmed a significant gain in the overall ITT eradication proportion with 10-day SEQ compared to STT. Many studies (60%) were reported to be ‘truly randomised’ (as defined in assessment of risk of bias in included studies) and therefore were unlikely to have been subjected to selection bias due to a lack of randomization through sequence generation.

Performance bias due to a lack of blinding of study participants and personnel could be the domain influencing the review’s findings, since over 50% of studies were recorded as not blinded. However, as stated in the assessment of risk of bias in included studies, all studies were classified as 'low risk', given the importance of this finding in the context of *H. pylori* eradication is low, as likewise addressed in the Discussion section.

Allocation

Nineteen studies [44,49,54-56,59,71,77-79,81,86,89,96,97,99-101,105] were rated as having poor allocation concealment and were flagged as 'high risk'.

Twenty-six (37%) studies reported that allocation was concealed and the remaining ones did not report any information on the allocation of the sequence generation and were therefore flagged as unclear (Figure 2).

In order to generate an unpredictable and unbiased sequence, 24 (35%) studies reported ‘adequate’ concealment of the allocation sequence, mainly using opaque sealed envelopes and by involving personnel in the enrolment phase that were unaware of the upcoming assignment of participants to treatments.

Albrecht, 2011, reported that the intervention sets were prepared by the hospital’s pharmacy and by independent personnel not involved in the study. Similarly, in Kim, 2011, only the independent staff could manage a matching list between study identification number and hospital number, and the data were only revealed to other investigators once recruitment and data collection were completed.

Blinding

We recorded 54 (78%) studies not preserving masking, as authors reported either that the trial was not blinded, the design of the study was open label or there was no information regarding this domain. In five studies [46,63,69,84,95], the authors stated that only the investigators (but not the participants) were blinded to the treatment allocation, in which case, we reported the studies as single-blinded (Figure 2). All of these studies were, however, classified as 'low risk' (as explained in blinding (performance bias and detection bias)).

The studies by Vaira, 2007 [43], and Albrecht, 2011 [88], were reported to use a ‘double-blind’ design with placebo during three days after completion of STT. With only two studies reported as double-blinded, we could not conduct the planned subgroup meta-analysis indicated in the protocol. The eradication proportions were 89% and 86% in the SEQ therapy arms and 77% and 69% in the STT therapy arms in Vaira, 2007 [43], and Albrecht, 2011 [88], respectively.

It should nonetheless be noted that the number of studies that were not blinded were due to the design of the SEQ regimen, where usually two drugs were used in the initial phase and three drugs during the second phase of treatment (as PP). Due to the manner in which the drugs were administered, participants could not be easily blinded to their assigned treatment.

Incomplete outcome data

Primary outcomes were correctly and consistently reported in all studies (Figure 3) as PP selection criteria. Attrition bias was reported in five studies [60,65,78,97,101].

Information related to the medical condition at baseline, sex ratio, average age of the population, PP sample size, incidence of AEs or antibiotic resistance were scarcely described in the reports of abstracts of congresses.

In Laving, 2013 [91], data regarding eradication were reported as the number of participants eradicated separately by stool antigen negative and histology negative results. Also, in this same study, the authors did not provide the eradication proportions by ITT analysis, but they could, however, be calculated. In the study by Choi, 2019 [101], the primary outcome was reported as a percentage and has to be also calculated. Additionally, in the later study, the eradication rate in the PP analysis, the compliance rate and adverse effects rate was not reported homogeneously in all treatment arms.

We noted no differences in the number of excluded participants or dropouts between treatment arms across the included studies.

Selective reporting

All studies reported, by treatment arm, the data of the primary outcome.

Thirteen (19%) studies reported *H. pylori* eradication proportions for those patients with bacterial antibiotic resistance: thirteen studies in patients with clarithromycin bacterial resistance; eleven studies in patients with nitroimidazole bacterial resistance; and eight studies in patients with dual bacterial resistance. A selective reporting bias was likely to be associated with this outcome.

Other potential sources of bias

Fifty-seven (83%) studies were in complete article form, indicating no bias was likely due to the publication status.

Studies were of mixed quality. Eradication was evaluated in subgroup analyses and the evidence was further assessed using GRADE. We included these subgroups in which eradication was found to be significantly different among groups or where subgroups were thought to influence *H. pylori* treatment efficacy in the summary of findings Table 1.

We downgraded the quality of the RCT evidence for the following outcomes: publication date (moderate quality), geographic region (low quality) and antibiotic resistance (very low quality). The analyses based on STT length and the adverse event rate were rated as high quality.

File S2: Detailed search strategies in each of the databases.

Search strategies

EBM Reviews - Cochrane Central Register of Controlled Trials search strategy
Via OVID platform

1. Helicobacter pylori/
2. pylori.mp. [mp=title, original title, abstract, mesh headings, heading words, keyword]
3. Helicobacter Infections/
4. or/1-3
5. ((triple or standard) adj2 (regimen or therapy or treatment)).tw.
6. (sequential adj2 (regimen or therapy or treatment)).tw.
7. PPI.mp.
8. Proton Pump Inhibitors/
9. (Clarithromycin or biaxin or Claripen or Claridar or clarith or Crixan or Clacid or Fromilid or infex or klaricid or Klabax or Klacid or Vikrol).mp.
10. (amoxicillin or amoxycillin or actimoxi or almodan or amix or amox or amopen or amoram or amoxicot or amoxil or amrit or biomox or clamoxyl or dispermox or galenamox or larotid or moxatag or moxilin or p-hydroxyampicillin or penamox or polymox or respillin or rimoxallin or senox or sumox or Tormoxin or trimox or utimox or wymox or zoxycil).mp.
11. (Alphamox or Amocla or Amoksibos or Amoxiclav Sandoz or Amoxidal or Amoxin or Amoksiklav or Amoxibiotic or Amoxicilina or ApoAmoxi or Augmentin or Bactox or Betalaktam or Cilamox or Curam or Dedoxil or Duomox or E-Mox or Enhancin or Gimalxina or Geramox or Hiconcil or Isimoxin or Klavox or Lamoxy or Moxilen or Moxypen or Moxyvit or Nobactam or Novamoxin or Ospamox or Panklav or Pamoxicillin or Panamox or Samthongcillin or Sinacilin or Tolodina or Yucla or Zerrsox or Zimox).mp.
12. nitroimidazoles/ or metronidazole/ or tinidazole/
13. nitroimidazole*.tw.
14. (Metronidazole or nabact or clont or danizol or edg dentalgel or elyzol or flagyl or gineflavir or metrocream or metrodzhil or metrogel or metrolotion or metrolyl or metronizole or metrotop or metrovex or metrozol or metryl or noritate or norzol or nydamax or obagi or protostat or rozex or satric or trichopol or tricom or trivazol or vandazole or vitazol or zadstat or zidoval).mp.
15. (Tinidazole or bioshik or fasigin or fasigyn* or tindamax or tricolam).tw.
16. or/5-15
17. 4 and 16

MEDLINE search strategy
Via OVID platform

1. randomized controlled trial.pt.
2. controlled clinical trial.pt.
3. randomized.ab.
4. placebo.ab.
5. clinical trials as topic.sh.
6. randomly.ab.
7. trial.ti.
8. or/1-7
9. exp animals/ not humans.sh.
10. 8 not 9
11. Helicobacter pylori/
12. pylori.mp. [mp=title, original title, abstract, name of substance word, subject heading word, unique identifier]
13. Helicobacter Infections/
14. or/11-13
15. ((triple or standard) adj2 (regimen or therapy or treatment)).tw.

16. (sequential adj2 (regimen or therapy or treatment)).tw.
17. PPI.mp.
18. Proton Pump Inhibitors/
19. (Clarithromycin or biaxin or Claripen or Claridar or clarith or Crixan or Clacid or Fromilid or infex or klaricid or Klabax or Klacid or
Vikrol).mp.
20. (amoxicillin or amoxycillin or actimoxi or almodan or amix or amox or amopen or amoram or amoxicot or amoxil or amrit or biomox
or clamoxyl or dispermox or galenamox or larotid or moxatag or moxilin or p-hydroxyampicillin or penamox or polymox or respillin or
rimoxallin or senox or sumox or Tormoxin or trimox or utimox or wymox or zoxycil).mp.
21. (Alphamox or Amocla or Amoksibos or Amoxiclav Sandoz or Amoxidal or Amoxin or Amoksiklav or Amoxibiotic or Amoxicilina or ApoAmoxi or Augmentin or Bactox or Betalaktam or Cilamox or Curam or Dedoxil or Duomox or E-Mox or Enhancin or Gimalxina or Geramox
or Hiconcil or Isimoxin or Klavox or Lamoxy or Moxilen or Moxypen or Moxyvit or Nobactam or Novamoxin or Ospamox or Panklav or
Pamoxicillin or Panamox or Samthongcillin or Sinacilin or Tolodina or Yucla or Zerrsox or Zimox).mp.
22. nitroimidazoles/ or metronidazole/ or tinidazole/
23. nitroimidazole*.tw.
24. (Metronidazole or nabact or clont or danizol or edg dentalgel or elyzol or flagyl or gineflavir or metrocream or metrodzhil or metrogel
or metrolotion or metrolyl or metronizole or metrotop or metrovex or metrozol or metryl or noritate or norzol or nydamax or obagi or
protostat or rozex or satric or trichopol or tricom or trivazol or vandazole or vitazol or zadstat or zidoval).mp.
25. (Tinidazole or bioshik or fasigin or fasigyn* or tindamax or tricolam).mp.
26. or/15-25
27. 14 and 26
28. 10 and 27

EMBASE search strategy
Via OVID platform
1. Clinical trial/
2. Randomized controlled trial/
3. Randomization/
4. Single-Blind Method/
5. Double-Blind Method/
6. Cross-Over Studies/
7. Random Allocation/
8. Placebo/
9. Randomi?ed controlled trial$.tw.
10. Rct.tw.
11. Random allocation.tw.
12. Randomly allocated.tw.
13. Allocated randomly.tw.
14. (allocated adj2 random).tw.
15. Single blind$.tw.
16. Double blind$.tw.
17. ((treble or triple) adj blind$).tw.
18. Placebo$.tw.
19. Prospective study/
20. or/1-19
21. Case study/
22. Case report.tw.
23. Abstract report/ or letter/
24. or/21-23
25. 20 not 24
26. Helicobacter pylori/

27. pylori.mp. [mp=title, abstract, subject headings, heading word, drug trade name, original title, device manufacturer, drug
manufacturer]
28. Helicobacter Infections/
29. or/26-28
30. ((triple or standard) adj2 (regimen or therapy or treatment)).tw.
31. (sequential adj2 (regimen or therapy or treatment)).tw.
32. PPI.mp.
33. Proton Pump Inhibitors/
34. (Clarithromycin or biaxin or Claripen or Claridar or clarith or Crixan or Clacid or Fromilid or infex or klaricid or Klabax or Klacid or
Vikrol).mp.
35. (amoxicillin or amoxycillin or actimoxi or almodan or amix or amox or amopen or amoram or amoxicot or amoxil or amrit or biomox
or clamoxyl or dispermox or galenamox or larotid or moxatag or moxilin or p-hydroxyampicillin or penamox or polymox or respillin or
rimoxallin or senox or sumox or Tormoxin or trimox or utimox or wymox or zoxycil).mp.
36. (Alphamox or Amocla or Amoksibos or Amoxiclav Sandoz or Amoxidal or Amoxin or Amoksiklav or Amoxibiotic or Amoxicilina or ApoAmoxi or Augmentin or Bactox or Betalaktam or Cilamox or Curam or Dedoxil or Duomox or E-Mox or Enhancin or Gimalxina or Geramox
or Hiconcil or Isimoxin or Klavox or Lamoxy or Moxilen or Moxypen or Moxyvit or Nobactam or Novamoxin or Ospamox or Panklav or
Pamoxicillin or Panamox or Samthongcillin or Sinacilin or Tolodina or Yucla or Zerrsox or Zimox).mp.
37. nitroimidazoles/ or metronidazole/ or tinidazole/
38. nitroimidazole*.tw.
39. (Metronidazole or nabact or clont or danizol or edg dentalgel or elyzol or flagyl or gineflavir or metrocream or metrodzhil or metrogel
or metrolotion or metrolyl or metronizole or metrotop or metrovex or metrozol or metryl or noritate or norzol or nydamax or obagi or
protostat or rozex or satric or trichopol or tricom or trivazol or vandazole or vitazol or zadstat or zidoval).mp.
40. (Tinidazole or bioshik or fasigin or fasigyn* or tindamax or tricolam).tw.
41. or/30-40
42. 29 and 41
43. 25 and 42

CINAHL search strategy
Via OVID platform
S12 (S1 and S11)
S11 S2 or S3 or S4 or S5 or S6 or S7 or S8 or S9 or S10
S10 Tinidazole
S9 Metronidazole
S8 nitroimidazole*
S7 amoxicillin
S6 Clarithromycin
S5 Proton Pump Inhibitors
S4 PPI
S3 sequential and ((regimen or therapy or treatment))
S2 ((triple or standard)) and ((regimen or therapy or treatment))
S1 Helicobacter pylori

File S3: Methods: risk of bias assessment

Items evaluated in the Risk of Bias assessment

- Random sequence generation;
- Allocation concealment;
- Blinding of participants and personnel;
- Blinding of outcome assessment;
- Incomplete outcome data (attrition bias);
- Selective outcome reporting (reporting bias).

Generation of the treatment allocation

We considered a study to be a RCT if it was explicitly described as 'randomised'. This should include the use of words such as 'randomly', 'random' or 'randomisation'. We then rated the randomized trial as truly random, pseudo-random, non-random, not stated or unclear.

We defined a trial as 'truly random' if the allocation sequence was computer-generated or generated by a random number table, coin toss, shuffles or throwing dice. The person involved in the recruitment of participants should not be the one performing the procedure.

If the selection was based on patient hospital numbers, birth dates, visit dates, alternate allocation or other methods not involving a defined random mechanism but likely to produce an unpredictable sequence of numbers, we considered the trial to be 'pseudo random'.

We excluded studies in which the selection was based on participant or clinical preference, or any selection mechanism that could not be described as random. We also excluded studies that did not state whether the treatment was randomly allocated.

We classified studies which were identified as randomized trials, but which did not describe how the treatment allocation was generated, as having an 'unclear' generation of treatment allocation.

Concealment of the treatment allocation at randomization

A study was classified as concealed, unconcealed or unclear in the following situations [148].

We rated a study 'concealed' if the trial investigators were unaware of the allocation of each participant before they were entered into the trial. Adequate methods included central telephone randomization schemes, pharmacy-based schemes, sequentially numbered, opaque, sealed envelopes, sealed envelopes from a closed bag or the use of numbered or coded bottles or containers.

We rated the allocation as 'unconcealed' when trial investigators were aware of the allocation of each participant before they entered the trial. For example, when it was based on participant data, such as the date of birth or hospital case note number, visit dates, sealed envelopes that were not opaque or a random number table that was not concealed from the investigator.

If authors did not report or provide a description of an allocation concealment approach that allowed for classification as concealed or not concealed, then we categorized the study as 'unclear allocation concealment'.

Implementation of masking

A trial could be considered double-blinded, single-blinded, not blinded or unclear and was to be classified within a 'Risk of bias' table into one of three categories: low risk, unclear risk and high risk [134].

We judged a study as 'not blinded' if the authors defined it as an open-label study, or no information was provided.

If a trial was simply described as 'single-blind', we recorded the degree of masking as not explicitly reported for clinician and outcome assessor, while participants were presumed to be blinded.

If a trial was reported as 'double-blind', we understood masking was performed at all levels. Double-blinding, however, was unlikely, as the type of treatment

administration could not easily allow for the simultaneous blinding of the clinician, the pharmacist, the participant and ultimately the outcome assessor.

In the context of studies evaluating *H. pylori* eradication treatments, the item blinding of participants and personnel was classified as 'low risk' in all included studies (independently if they were open-labeled, single- or double-blinded), as the implementation of masking at this level has no effect on the result of the eradication treatment (i.e., either success or failure).

On the other hand, the item blinding of outcome assessment was classified as 'low risk' in all included studies as well, given it was assumed that blinding during the evaluation (reading) of the outcome result (of the diagnostic method) would not alter the result of the test (i.e., *H. pylori* positive or negative). In the current therapeutic context, the result of the diagnostic method used to confirm *H. pylori* eradication is usually assessed either by a machine (in this case, for instance, the urea breath test and the stool antigen test) or by a pathologist (i.e., the study of a gastric biopsy through histology) who is usually unaware of the treatment assignment or any other test result.

File S4. Quality of the body of evidence (GRADE methodology)

We assessed the quality of the body of the evidence using GRADE methodology in those subgroup analyses where we found statistically significant differences between treatments for the main outcome.

We have incorporated these outcomes into the 'Summary of findings table' (Table S2) for the SEQ versus STT comparison. We present GRADE quality assessments ranging from 'very low' to 'high' quality evidence, alongside the effect estimates and decisions made relating to the downgrading (or upgrading) of evidence.

The GRADE approach uses five considerations, study limitations, consistency of effect, imprecision, indirectness and publication bias, to assess the quality of the body of evidence for each outcome. The evidence was downgraded from 'high quality' by one level for serious (or by two levels for very serious) limitations, depending on assessments of risk of bias, indirectness of evidence, serious inconsistency, imprecision of effect estimates or potential publication bias.

Sensitivity analysis

No arbitrary inclusion or exclusion criteria were established for the search strategy. If during the review process we identified sensitivity issues (missing data or individual peculiarities of the studies), we repeated the meta-analysis to test for differences. We conducted sensitivity analyses to test the robustness of the review, using a random effects model instead of a fixed-effect model; excluding trials with no or unclear allocation concealment; and excluding trials where the method of randomization was unclear.

Summary of findings and assessment of the certainty of the evidence

We used the GRADE approach which evaluates the following four levels, study limitations (risk of bias), inconsistency, imprecision, indirectness and publication bias (or other concerns) to assess the certainty of the evidence [149]. Two review authors (OPN and BM) independently made judgements regarding the certainty of evidence. A third review author (JPG) checked these judgements, and disagreements were resolved by consensus.

A summary of findings (SoF) table (Table S2) was created using GRADEpro GDT for the main comparisons that could potentially affect the main outcome (eradication): overall eradication rate, geographic region, publication date, STT different durations, bacterial antibiotic resistance and AEs.

The certainty of the body of the evidence was downgraded using the recommendations described in the *Cochrane Handbook for Systematic Reviews of Interventions*. The GRADE approach interprets the four aforementioned levels of certainty as follows:

- High certainty: we are very confident that the true effect lies close to that of the estimate of the effect.
- Moderate certainty: we are moderately confident in the effect estimate; the true effect is likely to be close to the estimate of the effect, but there is a possibility that it is substantially different.
- Low certainty: our confidence in the effect estimate is limited; the true effect may be substantially different from the estimate of the effect.
- Very low certainty: we have very little confidence in the effect estimate; the true effect is likely to be substantially different from the estimate of effect.

The decisions taken to downgrade the certainty of evidence in each outcome evaluated were detailed in the footnotes of the SoF table (Table S1).
